# Tumor-propagating side population cells are a dynamic subpopulation in undifferentiated pleomorphic sarcoma

**DOI:** 10.1172/jci.insight.148768

**Published:** 2021-11-22

**Authors:** Yuning Jackie Tang, Vijitha Puviindran, Yu Xiang, Yasuhito Yahara, Hongyuan Zhang, Puviindran Nadesan, Yarui Diao, David G. Kirsch, Benjamin A. Alman

**Affiliations:** 1 Department of Laboratory Medicine and Pathobiology, University of Toronto, Toronto, Ontario, Canada.; 2 Department of Orthopedic Surgery,; 3 Department of Cell Biology,; 4 Regeneration Next Initiative,; 5 Department of Pharmacology and Cancer Biology, and; 6 Department of Radiation Oncology, Duke University School of Medicine, Duke University, Durham, North Carolina, USA.

**Keywords:** Oncology, Cancer, Mouse models

## Abstract

Sarcomas contain a subpopulation of tumor-propagating cells (TPCs) with enhanced tumor-initiating and self-renewal properties. However, it is unclear whether the TPC phenotype in sarcomas is stable or a dynamic cell state that can derive from non-TPCs. In this study, we utilized a mouse model of undifferentiated pleomorphic sarcoma (UPS) to trace the lineage relationship between sarcoma side population (SP) cells that are enriched for TPCs and non-SP cells. By cotransplanting SP and non-SP cells expressing different endogenous fluorescent reporters, we show that non-SP cells can give rise to SP cells with enhanced tumor-propagating potential in vivo. Lineage trajectory analysis using single-cell RNA sequencing from SP and non-SP cells supports the notion that non-SP cells can assume the SP cell phenotype de novo. To test the effect of eradicating SP cells on tumor growth and self-renewal, we generated mouse sarcomas in which the diphtheria toxin receptor is expressed in the SP cells and their progeny. Ablation of the SP population using diphtheria toxin did not impede tumor growth or self-renewal. Altogether, we show that the sarcoma SP represent a dynamic cell state and targeting TPCs alone is insufficient to eliminate tumor progression.

## Graphical abstract

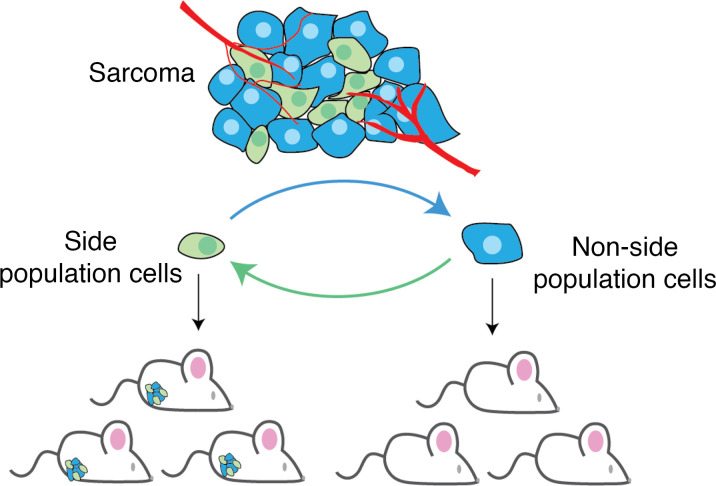

## Introduction

Neoplastic cells within tumors exhibit distinct genetic, epigenetic, and morphological properties (1). This heterogeneity is driven by the diverse genetic, epigenetic, and molecular alterations accumulated in individual tumor clones during the course of tumor progression (2–5). These distinct alterations endow phenotypic differences among the various subpopulations of tumor cells and contribute to therapeutic failure (6). In sarcomas and many other cancer types, a subpopulation of tumor-propagating cells (TPCs) with stem cell–like characteristics has been identified and is thought to be capable of self-renewal and giving rise to other cancer cell subpopulations in the tumor (7, 8). Functionally, TPCs are defined by their ability to continuously propagate the tumor in serial transplantation experiments in immunodeficient animal hosts (8). Importantly, TPCs have also been shown to display increased therapy resistance; thus, their selective eradication is hypothesized to be essential for cancer therapy (9). Multiple studies have characterized and targeted TPCs to impede tumor progression in a wide variety of cancer types (10, 11). Integral to these studies is the assumption that the lineage relationship between TPCs and non-TPCs is unidirectional, where TPCs alone can give rise to non-TPCs. However, emerging evidence in normal tissues suggests there is substantial lineage plasticity, especially within the context of injury, where differentiated cells can express stem cell markers and acquire stem cell properties (12, 13). For instance, differentiated hepatocytes can convert to bipotential progenitors following chemical injury (14). Similarly, loss of basal stem cells in the lung epithelium can promote the dedifferentiation of luminal secretory cells to basal stem cells (15). Broad cellular dedifferentiation and disruption of lineage specifying transcription factors are a well-recognized phenomenon in tumors (16–18). Nonetheless, whether lineage plasticity exists between TPCs and non-TPCs in sarcomas has not been explored.

Undifferentiated pleomorphic sarcoma (UPS) is the most diagnosed soft-tissue sarcoma in adults. This tumor type comprises heterogeneous cells with distinct cytological features, epigenetic changes, and phenotypical behaviors (19–21). Cells with TPC characteristics can be prospectively isolated from patient UPS tumors and other sarcomas subtypes using the side population (SP) assay (22–24). Here, we investigate whether TPC-enriched SP cells in sarcoma are a stable population or a dynamic cell state. Using lineage tracing, single-cell RNA sequencing (scRNA-Seq), and genetic ablation, we demonstrate that non-SP cells can give rise to SP cells in mouse models of UPS de novo. Selective depletion of SP cells and their progenies are not able to impede tumor growth or reduce tumor self-renewal.

## Results

### SP cells identify TPCs in murine UPS.

**Figure 1:**
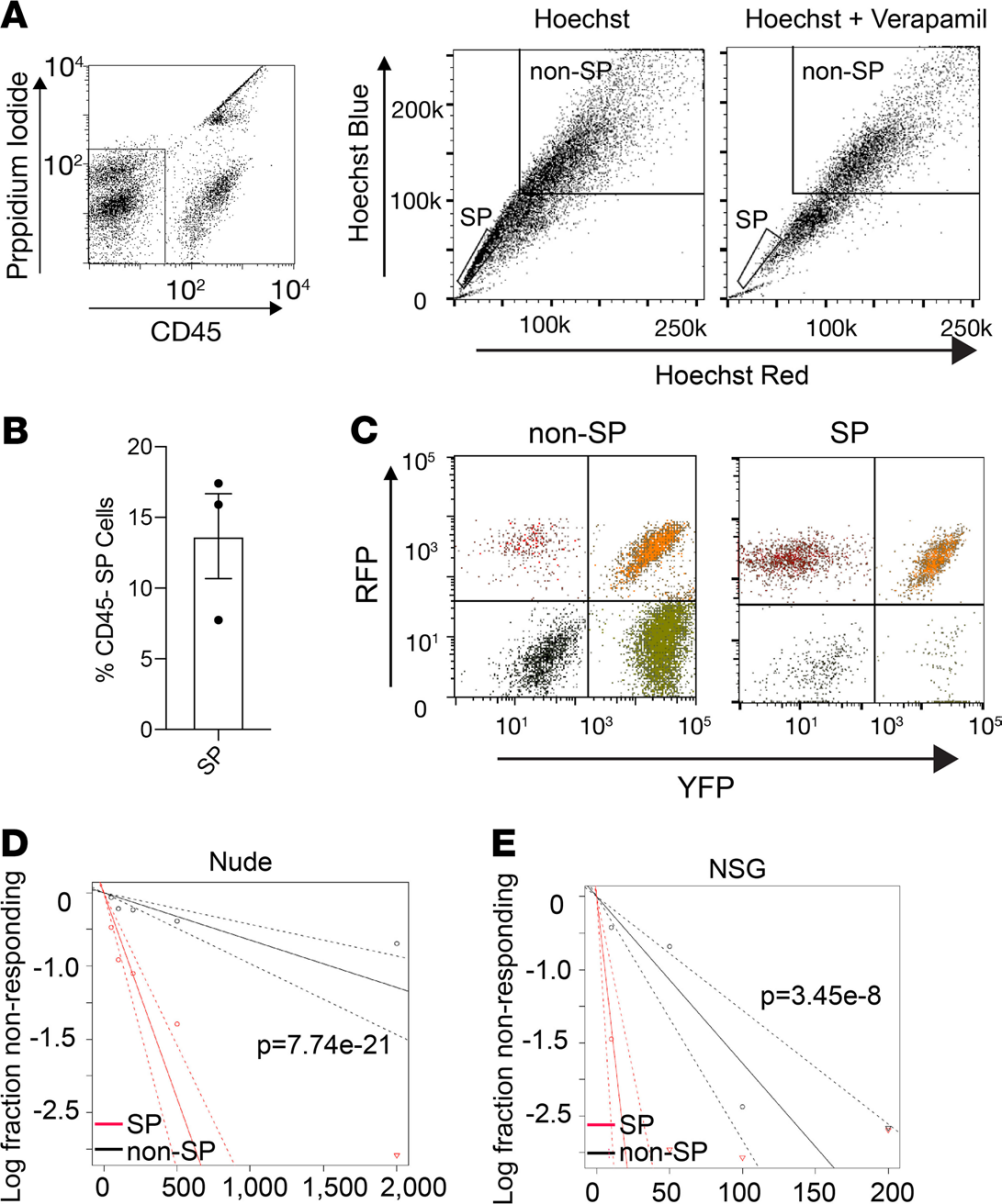
Side population cells in KPCC tumors enrich for TPCs. (**A**) Representative FACS gating schematic for side population (SP) and non-SP cells in KPCC tumors (*n* > 3). (**B**) Average percentage of CD45^–^ SP cells in KPCC tumors (*n* = 3). Error bars represent mean ± SEM. (**C**) SP and non-SP cells in KPCC tumors express different florescent reporters (*n* = 3). (**D**) Extreme limiting dilution analysis (ELDA) shows that SP cells are significantly enriched for tumor-propagating potential compared with non-SP cells in nude mice (χ^2^ = 87.7, df = 1, *P* = 7.74 × 10^–21^). The dotted line indicates the 95% confidence interval. (**E**) ELDA shows that SP cells are significantly enriched for tumor-propagating potential compared with non-SP cells in NSG mice (χ^2^ = 30.4, df = 1, *P* = 3.45 × 10^–8^). The dotted line indicates the 95% confidence interval.

To trace TPCs in vivo, we utilized a mouse model of spatially and temporally restricted UPS driven by conditional Cre recombinase–induced activation of oncogenic *Kras^G12D^* mutation and homozygous deletion of *Trp*53 (25). Injection of adenovirus-expressing Cre recombinase into the gastrocnemius muscle of this model induces UPS tumors at the injection site. We crossed this model with the multifluorescent lineage reporter allele R26R-Confetti to generate *Kras^LSL-G12D^; Trp53^f/f^;* R26R-Confetti (KPCC) mice. The R26R-Confetti alleles stably label the different tumor clones with distinct fluorescent reporters (26–28). SP cells are enriched for TPCs in several types of human sarcoma (22, 23). Hence, we sought to trace the lineage relationship between SP and non-SP cells in KPCC mice. We digested primary tumors and stained the cells with Hoechst 33342 dye, while excluding cells from the hematopoietic lineage based on CD45 expression (Figure 1A). In KPCC tumors, the CD45^–^ SP cells represented 13.68% (±3.0% SEM) of total tumor cells (Figure 1B). In human sarcomas, the SP fraction has self-renewal capacity in serial transplantations (22). Furthermore, in the KPCC model, both the SP and non-SP cells consisted of cells labeled by distinct fluorescent reporters (Figure 1C), suggesting they are polyclonal. To test the tumor-propagating potential of SP and non-SP cells in the KPCC mouse model, we orthotopically transplanted the 2 populations at limiting dilutions in *Foxn1^nu/nu^* nude mice. SP cells formed significantly more tumors than non-SP cells when analyzed by extreme limiting dilution analysis (ELDA) (29) (Table 1 and Figure 1D). Because the degree of immunodeficiency of the animal hosts is known to influence the ability of tumor cells to engraft (30), we also performed limiting dilution transplant experiments in the NOD/SCID IL2rγ^null^ (NSG) mice. Consistent with their more permissive immune system, fewer cells overall were required to form tumors in the NSG mice compared with *Foxn1^nu/nu^* nude mice (Table 2). However, SP cells remained significantly more tumorigenic compared with non-SP cells (Figure 1E). Taken together, SP cells in the KPCC model are enriched for tumor-propagating potential and are labeled with different fluorescent reporters.

### SP cells can be derived from non-SP cells de novo.

**Figure 2:**
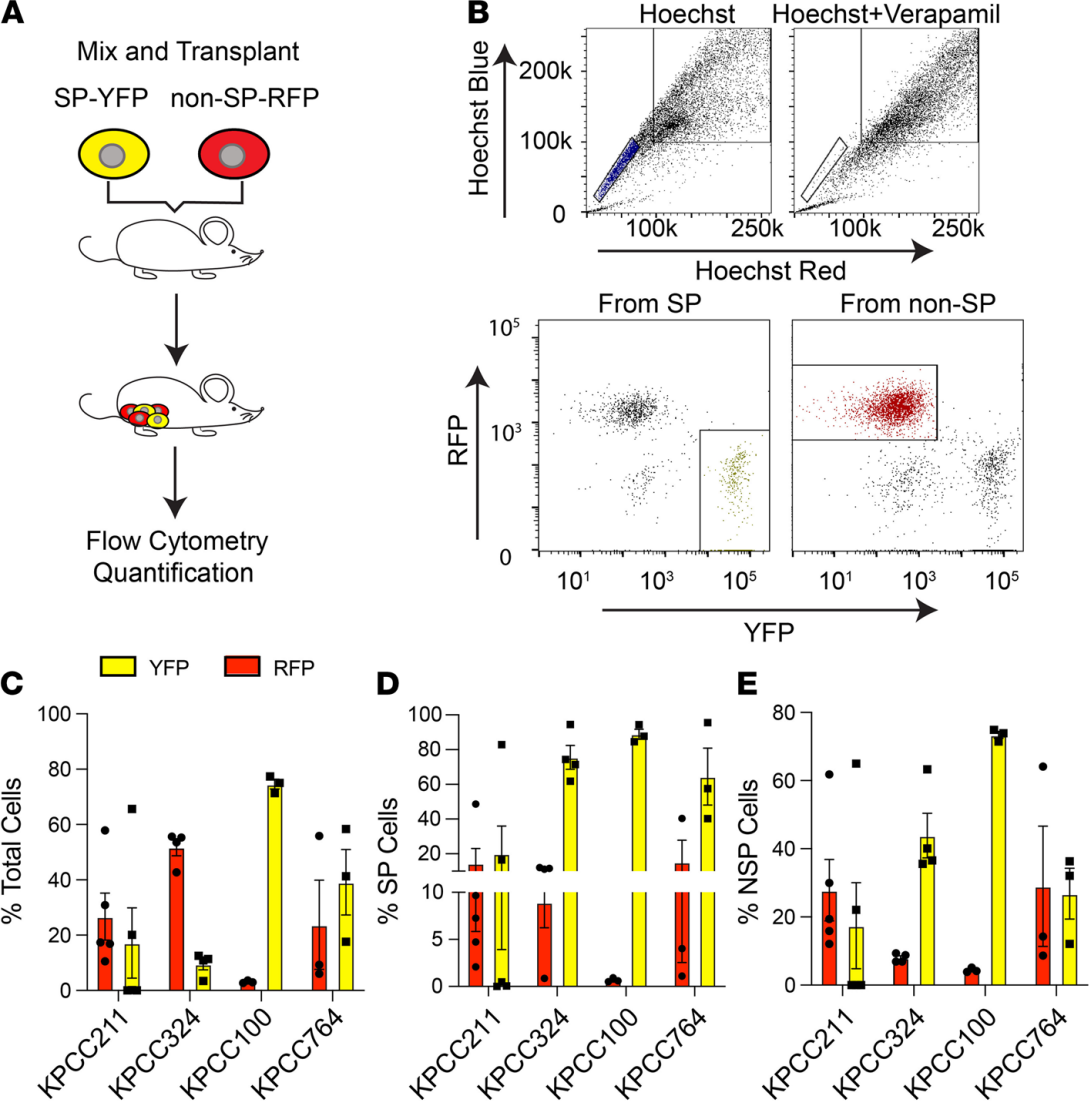
Cotransplantation of SP and non-SP cells expressing different florescent reporters reveals that NSP cells can give rise to SP cells in vivo. (**A**) Schematic of cotransplantation experiment. (**B**) FACS gating to sort for SP cells expressing YFP and non-SP cells expressing RFP from KPCC tumors (*n* = 4). (**C–E**) The percentages of cells expressing RFP and YFP within the total cell population, the SP compartment, and the non-SP compartment. Each dot represents a mouse. Error bars represent mean ± SEM.

To investigate the phenotypic stability of TPCs in sarcoma, we took advantage of the differently labeled tumor cells in the KPCC model and isolated the SP population expressing YFP and non-SP cells expressing RFP to trace these cell subpopulations in cotransplant experiments. To ensure the purity of our flow cytometry, we reanalyzed SP and non-SP cells after sorting and found a high degree of purity in the sorted cells (Supplemental Figure 1; supplemental material available online with this article; https://doi.org/10.1172/jci.insight.148768DS1). We used 4 independent primary KPCC tumors and cotransplanted the sorted SP-YFP and non-SP-RFP cells into *Foxn1^nu/nu^* mice (*n* = 3–5 for each KPCC tumor) at an average ratio of approximately 1 SP/3 non-SP (Figure 2A) to lineage trace the 2 subpopulations. When the cotransplanted tumors reached 350–600 mm^3^, the tumors were digested and analyzed by flow cytometry (Figure 2B). We found that both RFP- and YFP-expressing cells survived and proliferated in the transplanted tumor (Figure 2C). Importantly, we found RFP expressing-cells within the SP compartment of the cotransplanted tumors, indicating that non-SP cells are able to convert to SP phenotype de novo during tumor growth (Figure 2D). In the non-SP compartment, there were YFP-expressing cells, consistent with SP cells giving rise to non-SP cells (Figure 2E).

To test whether the SP cells derived from the non-SP cells have TPC properties, we compared the tumor-propagating potential of RFP-expressing SP and non-SP cells isolated from the coinjection experiments at limiting dilutions. Compared with non-SP cells, the RFP-expressing SP cells were significantly more tumorigenic (Supplemental Figure 2). Collectively, these data suggest that SP cells enriched for tumor-propagating potential can derive from non-SP cells in vivo.

### scRNA-Seq analysis of SP and non-SP cells.

**Figure 3:**
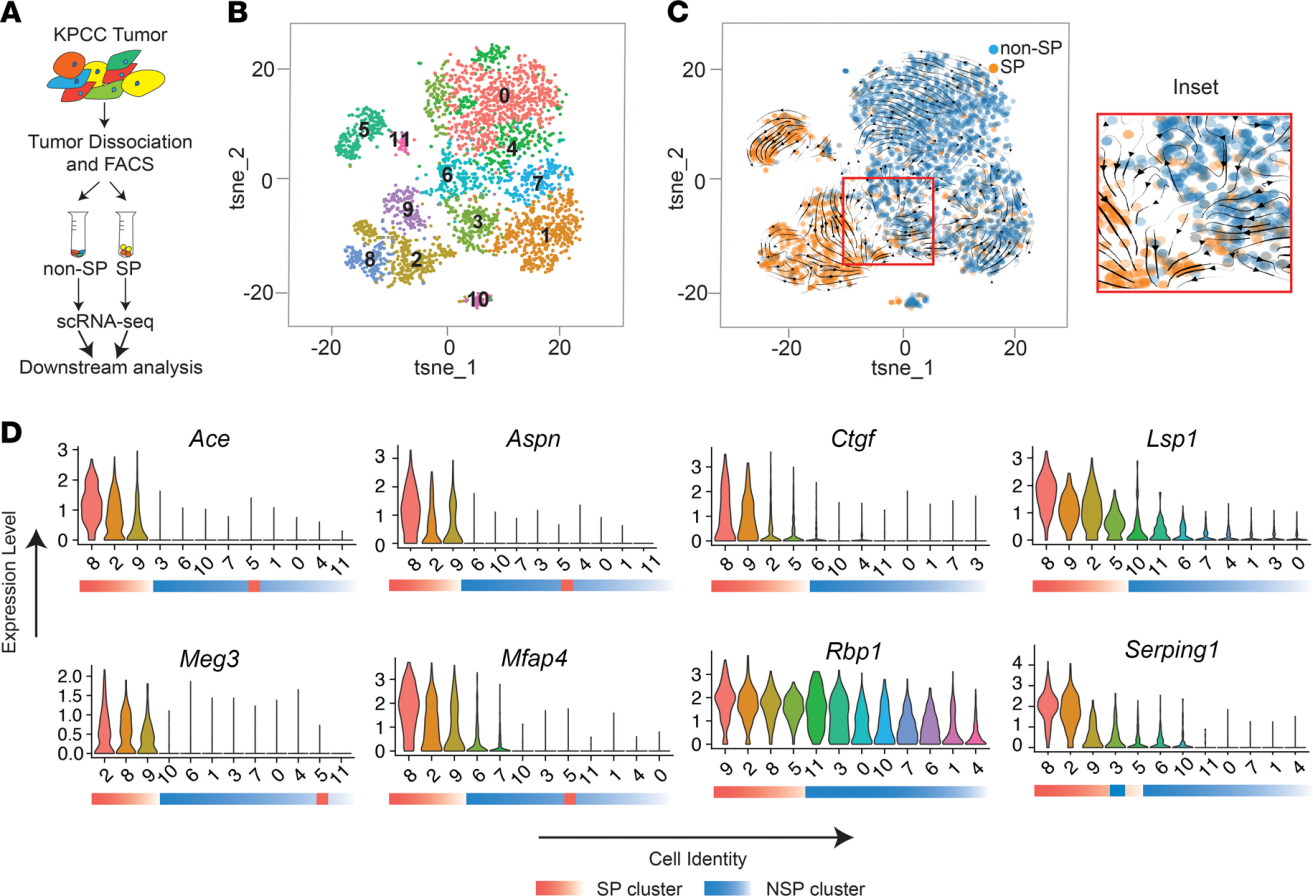
RNA velocity analysis of SP and non-SP populations. (**A**) Schematic of the scRNA-Seq experiment. (**B**) T-distributed stochastic neighbor embedding (tSNE) plot showing different clusters in the SP and non-SP cells from KPCC-844 tumor. (**C**) RNA velocity analysis showing predicted cell fate transition from non-SP to SP population. (**D**) Violin plot showing expressions of marker genes in SP and NSP cells. Cell clusters are arranged by decreasing gene expression. Each cell identity number corresponds to a cell identity number in the tSNE plot.

Transcriptome analysis at single-cell resolution is a powerful tool for studying cellular states and lineage dynamics within complex tissues and tumor samples (31, 32). To better understand the lineage relationship between SP and non-SP cells, we sorted SP and non-SP populations from 3 independent KPCC tumors and performed scRNA-Seq on these populations. We combined the SP and non-SP scRNA-Seq data from each paired sample and filtered for cells that express fluorescent reporters to enrich for tumor cells (Figure 3A). We profiled 6080 SP cells and 6806 non-SP cells from the 3 samples. Pseudo-bulk gene expression analysis between SP and non-SP cells showed that genes associated with negative regulation of developmental processes and cell differentiation were upregulated, while genes associated with cell cycle were downregulated, congruent with the more progenitor cell–like and quiescent phenotypes associated with TPCs (Supplemental Tables 1–4). Cluster analysis showed that both the SP and non-SP populations contain multiple cell clusters, reflecting the heterogeneity in gene expression within each subpopulation (Figure 3B and Supplemental Figure 3). To explore the lineage relationship of SP and non-SP cells using scRNA-Seq data, we carried out pseudotime analysis using RNA velocity. RNA velocity leverages the relative abundance of pre-mRNA to mature mRNA to predict future states of individual cells (35, 36). In each KPCC tumor, RNA velocity suggested that some non-SP cells give rise to SP cells (Figure 3C and Supplemental Figure 3, B and E). These data are consistent with our in vivo lineage-tracing experiments, demonstrating that SP cells can derive from non-SP cells.

Marker genes that identify sarcoma TPCs are poorly understood. To investigate candidate marker genes, we looked for common genes that are highly expressed in clusters of SP cells across all 3 KPCC tumors. We searched for genes that were expressed in the SP clusters with an average log fold change of >0.5, detected in at least 35% of SP cells, and were consistently upregulated in most clusters in the SP population. We identified 8 marker genes, including *Ace*, *Aspn*, *Ctgf*, *Lsp*, *Meg3*, *Mfap4*, *Rbp1*, and *Serging1* (Figure 3D and Supplemental Figures 3 and 4). To better understand the expression of these marker genes in muscle tissues, we analyzed scRNA-Seq data from human and mouse muscles. The marker genes are substantially upregulated in muscle satellite cells, fibroadipogenic progenitors (FAPs), and mesenchymal stromal cells (MSCs) compared with other cell types in normal human and mouse muscles (Supplemental Figures 5 and 6). The high expression of SP cell marker genes in the progenitor populations of normal muscles is consistent with the increased stemness of TPCs.

Moreover, to better understand the gene expression differences between different tumor clones, we compared the scRNA-Seq data between RFP and YFP cells for both SP and non-SP population. The top gene sets upregulated in RFP-expressing cells compared with YFP-expressing cells for both SP and non-SP populations were associated with ribosome assembly, cell cycle, and RNA processing. Genes sets associated with oxidation, collagen, plasminogen, and angiogenesis were downregulated (Supplemental Tables 5 and 6). Specific to SP cells, RFP-expressing cells had upregulated genes associated with telomere localization and senescence compared with YFP-expressing cells; genes involved in response to IGF receptor (*Igfr*) signaling and *Tgf-*β production were downregulated. Within the non-SP population, RFP-expressing cells showed upregulation in mitochondrial metabolism and downregulation of genes involved in PDGF receptor (*Pdgfr*) signaling, epithelial-mesenchymal transition, and wound healing (Supplemental Tables 7 and 8). These differences are reflective of the heterogeneity in gene expression between different tumor clones (4, 33, 34).

### Genetic ablation of SP cells in sarcomas does not inhibit tumor self renewal.

**Figure 4:**
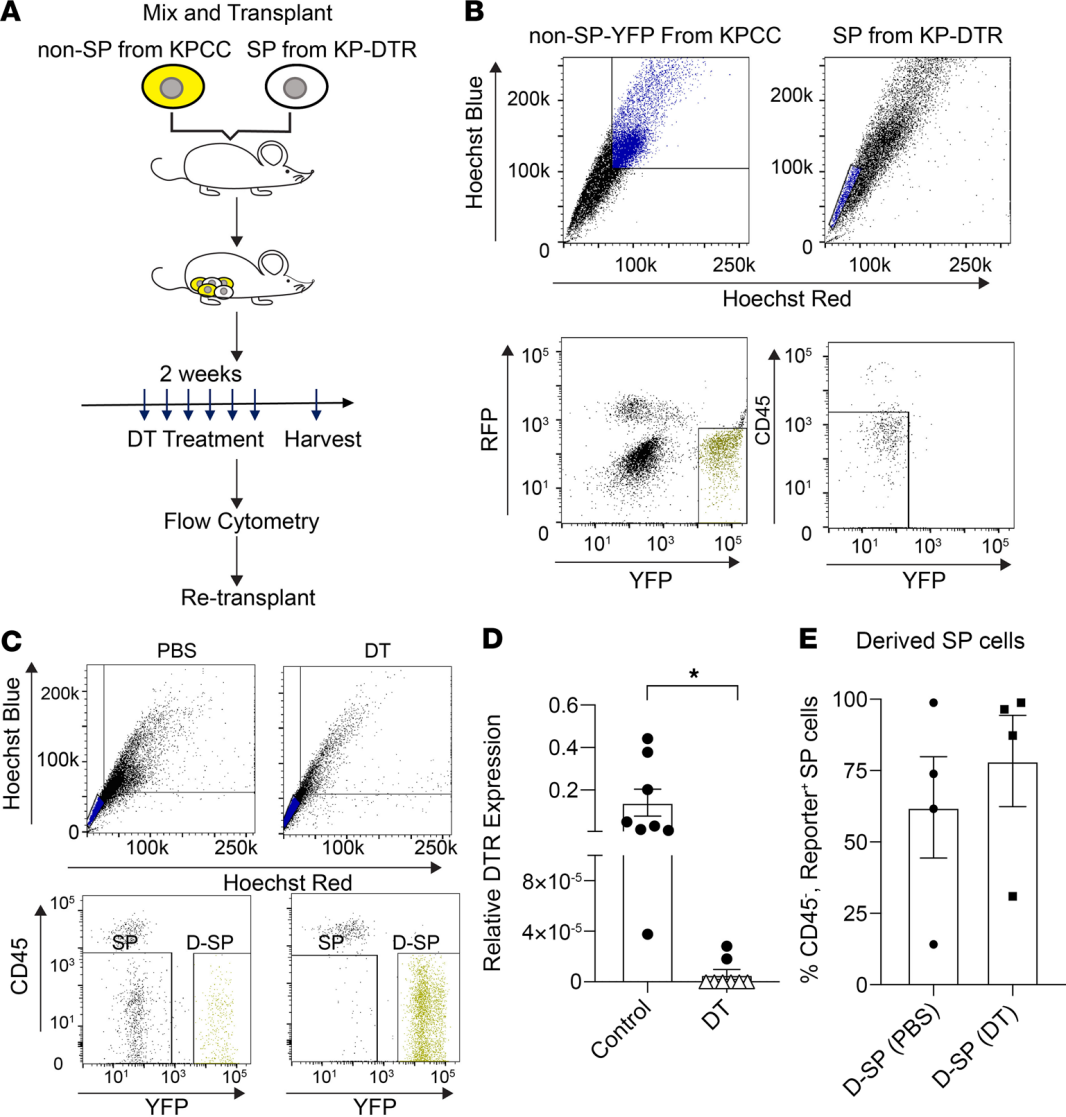
Cotransplantation and ablation of SP cells in vivo. (**A**) Schematic of cotransplantation and genetic ablation of SP cells expressing diphtheria toxin receptor (DTR) gene. (**B**) FACS gating scheme to sort for non-SP cells from KPCC tumors and SP cells from KP-DTR tumors for cotransplantation (*n* = 4). (**C**) FACS gating of SP and non-SP cells after the tumors are treated with diphtheria toxin (DT) or PBS. (**D**) The relative expression of the DTR gene for tumors treated with DT compared with tumors treated with 1X PBS. Each symbol represent tumors from a cotransplanted mouse. Triangles represent mice with undetectable DTR expression (*P < 0.05, 2-tailed Student’s *t* test). Error bars represent mean ± SEM. (**E**) FACS analysis of mean SP cells expressing fluorescent reporters in cotransplanted tumors after DT or PBS treatment. Each dot represents the mean percentage of fluorescent SP cells from each set of cotransplantation experiments (*n* ≥ 3 for each set). Error bars represent mean ± SEM.

Because of TPCs’ ability to continuously self-renew and propagate the tumor, it was proposed that targeted eradication of TPCs may be crucial for inhibiting tumor progression and recurrence (9). To test the effect of ablating TPCs during tumor growth, we generated *Kras^LSL-G12D^*; *Trp53^f/f^*; *Rosa26-DTR* (KP-DTR) mice. Injection of adenovirus-expressing Cre recombinase activates the oncogenic mutations to initiate UPS formation and the induce of diphtheria toxin receptor (DTR) expression in the transformed cells and their progeny. To specifically ablate SP cells in growing tumors, we isolated the CD45^–^ SP cells from KP-DTR tumors and mixed them with non-SP cells expressing fluorescent reporters from KPCC animals for orthotopic transplantation into the gastrocnemius muscle of nude mice (Figure 4, A and B). We performed 4 sets of SP-DTR and non-SP-KPCC cell cotransplantations. After tumor formation, the animals were randomly divided into 2 groups to receive either diphtheria toxin (DT) or vehicle (1X PBS). Overall, DT treatment had limited effects on tumor growth (Supplemental Figure 7). To confirm that DT was able to ablate the SP-DTR population, we performed qPCR for the expression of DTR in the transplanted tumors. Compared with that in tumors treated with vehicle, DTR expression was undetectable in most tumors treated with DT (Figure 4, C and D). This result indicates that DT treatment was effective in ablating SP-DTR cells in vivo. FACS analysis of the treated tumors revealed that 62.11% (±17.78% SEM) and 78.34% (±16.00% SEM) of SP population cells expressed fluorescent reporters in the PBS- and DT-treated tumors, respectively, indicating that they are derived from non-SP cells (Figure 4E).

Next, we performed transplantation assays at limiting dilutions to compare the self-renewal capacity of DT-treated tumors with that of the vehicle-treated control tumors. An equal number of cells derived from KPCC-DTR mixture tumors treated with DT or vehicle were injected into the gastrocnemius muscles of nude mice. Depletion of SP cells by DT treatment did not reduce tumor-initiating capacity compared with the vehicle (Table 3). This result suggests that non-SP cells compensate for SP cells in tumor-propagating capacity and that ablating SP cells alone is not sufficient to inhibit tumor self-renewal.

## Discussion

TPCs are functionally defined as cells with enhanced tumor-initiating and self-renewal capacity (35). From a therapeutic perspective, the elimination of TPCs is thought to be important to impede tumor growth and recurrence (36–39). Nonetheless, recent evidence in normal tissues suggests that the lineage hierarchy between stem cells and their differentiated progenies may be plastic, where the differentiated cells can acquire stem cell phenotypes under certain conditions, such as during tissue repair (15, 40, 41). To understand the lineage relationship between SP and non-SP cells, we performed lineage tracing and targeted depletion of SP cells in a mouse model of UPS. By cotransplanting SP and non-SP cells expressing different fluorescent reporters into immunodeficient mice, we showed that non-SP cells could give rise to SP cells de novo. This result is supported by in silico lineage analysis using scRNA-Seq data. To test the impact of eliminating SP cells on tumor growth and self-renewal, we orthotopically cotransplanted SP cells stably expressing DTR with non-SP cells. Ablation of SP cells by DT injection showed compensation of the SP fraction by non-SP cells in the ability to propagate a tumor, as the tumorigenic capacity of tumors treated with DT was similar to tumors treated with the control vehicle. Overall, our data suggest that TPCs, as defined by the SP phenotype, are likely a dynamic state rather than a stable cell population in UPS.

To date, few marker genes for the sarcoma TPCs have been identified. In this study, we identified 8 cell markers that are consistently upregulated in the different scRNA-Seq clusters of the SP population. These genes are also upregulated in progenitor cell populations of normal human and mouse muscles. FAPs, MSCs, and satellite cells all have been shown to be the cells of origin for UPS and other soft-tissue sarcomas (42–44). Several marker genes we identified play known roles in tissue development and self-renewal. Specifically, *Aspn* and *Ctgf* are crucial for mesenchymal progenitor cell self-renewal (45, 46). *Serping1* plays a role in stem cell proliferation and tissue development (47). In addition, *Meg3* regulates muscle development and regeneration (48).

Emerging evidence suggests that TPC plasticity may exist in multiple tumor types. In breast and pancreatic cancer, induction of gene expression programs associated with epithelial-mesenchymal transition can promote TPC properties (49–51). However, these studies primarily focused on cell lines and xenograft tumors engrafted away from the primary tumor site. Recently, studies using mouse organoids of colorectal cancer showed that Lgr5^–^ cancer cells can convert to Lgr5^+^ TPCs to reinitiate tumor growth, and this conversion may be essential for metastatic colonization (52). Importantly, studies of TPC plasticity are almost exclusively focused on epithelial tumors. In this study, we utilized autochthonous mouse models of UPS and orthotopic allografts to provide evidence of TPC plasticity in tumors that originate from the mesenchymal tissue. Nonetheless, our results do not rule out the possibility that the TPCs in sarcoma may contain heterogeneous cells that can be isolated by different methods. For example, in breast cancer, cells with TPC properties can be isolated by ALDH or CD44^+^/CD24^–^ markers (53). Therefore, it is conceivable that a minor subset of non-SP cells may harbor cells with TPC properties, which gave rise to SP cells. However, given the limited tumorigenic capacity of non-SP cells in our transplant models and other sarcoma models (22, 23), if these cells are present, they are likely rare and cannot fully account for the high percentage of non-SP-derived, highly tumorigenic SP cells observed in our study. The interchangeability between non-SP and SP cells supports a model in which sarcoma cells exhibit lineage plasticity and that the SP cells are a dynamic cell state.

The plasticity of cellular phenotypes can result from intrinsic properties and extrinsic cues (54). For example, dysregulation of cell-fate-specifying transcription factors, such as *Sox2* and *Nkx2.1*, can contribute to lineage plasticity and stemness in cancer cells (55, 56). Extrinsic cues from the tumor microenvironment can determine cancer cell heterogeneity and affect the TPC phenotype (57). Indeed, TPCs in head-and-neck cancers and glioblastoma reside in the perivascular niche and are supported by vascular endothelial cells to prevent apoptosis (58, 59). Furthermore, cancer-associated fibroblasts and hypoxia can stimulate stemness and promote TPC survival via activation of self-renewal pathways (60, 61). Additionally, the crosstalk among cancer cells can influence the phenotypic diversity of cancer cells. In breast cancer, a minor subpopulation of cells expressing *IL-11* can stimulate the growth of other cancer cells through a non-cell-autonomous manner (62). Moreover, breast cancer cells with a mesenchymal phenotype can promote tumorigenicity and self-renewal of nearby tumor cells via paracrine activation of Wnt signaling (63). Unlike epithelial cancers, sarcomas are derived from mesenchymal cells, which are known to have a high degree of plasticity during repair processes (64–66). It is possible that, similar to the cell type from which sarcomas are derived, sarcoma tumor cells may have a high level of plasticity due to molecular programs activated during repair. Investigations into the interactions among subpopulations of cancer cells or between cancer cells and the TPC-promoting microenvironment may reveal the mechanistic insights that contribute to TPC plasticity.

TPCs are hypothesized to be a valuable target of therapy because of their enhanced tumorigenicity, self-renewal, and therapy resistance. Clinically, the relative frequency of the SP fraction in sarcomas is associated with tumor grade in patients (22). Similarly, the percentage of TPCs and TPC biomarkers in other tumor types is frequently associated with more malignant histopathological features and patient survival (67, 68). However, the interconversion between SP and non-SP cells indicates that the elimination of TPCs at one point in time is unlikely to eradicate long-term tumor propagation and progression. Equally important, standard chemotherapy can increase the relative frequency of TPCs. For example, the percentage of SP cells increased in patient-derived sarcoma xenografts that are treated with the standard-of-care chemotherapy doxorubicin and cisplatin (69). This phenomenon of TPC enrichment after therapy has been largely attributed to enhanced resistance intrinsic to TPCs. It remains unclear whether the lineage plasticity between SP and non-SP cells is affected by conventional therapy in vivo. Studies show that self-renewal pathways, such as Wnt/β-catenin signaling, can be upregulated in response to therapy. Future studies on the amount of SP stability in association with different clinical characteristics and response to therapy may reveal the full extent of TPC plasticity in mesenchymal tumors.

## Methods

### Mouse models.

The R26R-Confetti (stock no. 013731) and R26R-LSL-DTR (stock no. 007900) mice were obtained from The Jackson Laboratories. The *Kras^LSL-G12D^* mice were provided by Tyler Jacks (MIT, Cambridge, Massachusetts, USA), and the *Trp53^f/f^* mice were provided by Anton Berns (NKI, Amsterdam, The Netherlands). All mice were on a mixed genetic background, and both male and female mice were included in the study. The animals were housed at room temperature with a 12-hour-light/dark cycle. To induce tumors, adenovirus-expressing Cre recombinase mixed with 50 μl of 2 M CaCl_2_ in DMEM were directly injected into the hind gastrocnemius muscle of 7- to 12-week-old mice. Growth of tumors was confirmed by physical examination at the injection site and by histology.

### Tumor dissociation.

Primary sarcoma tumors from KPCC and KP-DTR mice were mechanically and enzymatically dissociated into single-cell suspensions as previously described (22). Briefly, the tumors were mechanically dissociated into small pieces and enzymatically digested with a mixture of 10 mg/mL collagenase IV (Worthington), 2.4 U/ml Dispase (BD), and 0.05% trypsin. Red blood cells were excluded with ACK lysis buffer (Thermo Fisher Scientific). The digested mixture was filtered through a 45-micron strainer, and the single-cell suspensions were frozen down in liquid nitrogen using Synth-a-Freeze media following the manufacture’s protocol (Thermo Fisher Scientific).

### Flow cytometry.

FACS analyses were performed on BD FACSDiva Cell Sorter (BD FACSDiva Software, RRID:SCR_001456) at the Duke Flow Cytometry Core. For isolation of SP and non-SP cells, single-cell suspensions were treated with 2.5 mg/mL Hoechst 33342 dye (MilliporeSigma, B2261) alone, or in combination with 50 mmol/L verapamil (MilliporeSigma, V4629) as a negative control, for 90 minutes at 37°C. SP cells were identified using dual-wavelength analysis (blue, 424–444 nm; red, 675 nm) after excitation with 360 nm UV laser. To enrich for tumor SP cells, digested tumor mixture was stained with rat anti-mouse CD45-PE-Cy7 (BD Biosciences, 552848, RRID:AB_394489) or rat anti-mouse CD45-APC-Cy7 (BD Biosciences, 557659, RRID:AB_396774) antibody at 1:800 dilution, or cells were sorted on the expressed fluorescent reporters. RFP and YFP cells from the KPCC tumors were identified using the blue 488 nm laser. Dead cells were eliminated with propidium iodide (Thermo Fisher Scientific), according to the manufacturer’s instructions. To analyze the purity of the sorted cells, we took a small sample of sorted cells immediately after FACS and reanalyzed for the presence of different sorted fractions using the same sorter (FACSDiva Cell Sorter, RRID:SCR_001456). Analysis of flow cytometry data were performed using FloJo (version 10.7).

### Tumor transplantation and DT treatment.

For tumor transplantation experiments, 5- to 8-week-old male *Foxn1^nu/nu^* mice and *NOD-SCID IL2rγnull* mice were purchased from the Duke University Division of Laboratory Animals and Resources. Tumor cells were diluted into 1X PBS at different numbers, as indicated in the study. To inject the cells, the nude mice were anesthetized under 2% isoflurane. The cell solutions were injected into the left hind limb gastrocnemius muscle of nude mice and subcutaneously into NSG mice. Tumor formation was confirmed by physical examination. Tumor volume was determined by the following formula: (width × height × length)π/6. All animals were euthanized when the tumors reached approximately 1.0–1.2 cm in largest diameter. Enrichment of TPC population was determined by ELDM based on the number of tumors of the total number of mice transplanted at each cell dilution (29). For cotransplantation, SP cells expressing YFP and non-SP cells expressing RFP from the same tumor were sorted and mixed at an average ratio of 1:3 in 1X PBS and injected into the left gastrocnemius muscle. An average of 362 SP cells and 1550 non-SP cells were cotransplanted together. The resulting tumors were digested and analyzed by flow cytometry to determine the percentage of each fluorescent reporter in the SP and non-SP compartments. To generate the tumors mixed with non-SP cells from KPCC and SP cells KP-DTR cells, the SP and non-SP cells were mixed at an average ratio of 1:3.5 respectively. An average of 625 cells from the SP compartment and 2225 cells from the non-SP compartment were coinjected into the gastrocnemius muscle of nude mice. DT was obtained from MilliporeSigma and resuspended in 1X PBS. The animals were randomized to receive DT injected intraperitoneally at 250 μg/kg diluted in 100 μl or 1X PBS injection as vehicle. All injections were performed every other day for a total of 6–7 doses. The animals were monitored for signs of weight loss and changes in posture and mobility.

### RT-qPCR.

RNA was extracted from transplanted tumor cells derived from mixed KPCC and KP-DTR cells treated with PBS or DT using the Qiagen RNeasy Mini Kit. Reverse transcription of RNA to cDNA was performed using the iScript cDNA Synthesis kit (Bio-Rad) according to the manufacture’s instructions. Quantitative PCR was performed using SYBR green reagent (Invitrogen) on a QuantStudio 3 real-time PCR system (Thermo Fisher Scientific). Gene expression was calculated and expressed relative to housekeeping gene GAPDH using the standard ΔΔCT method. The sequence of the DTR gene was obtained from a previously published report (70). The following primers were used for the DTR gene: 5′-AGGTTACCATGGAGAGAGGT-3′ (sense); 5′-CCACAGCCAGGATAGTTGTATG-3′ (antisense).

### Library preparation for scRNA-Seq.

FACS-sorted SP and non-SP single-cell suspensions were loaded on the 10× Genomics Chromium Controller Single-Cell Instrument mixed with reverse transcription reagents along with gel beads and oil to generate single-cell gel beads in emulsions (GEMs) for reverse transcription. Reverse transcription of GEMs was performed in an Eppendorf Mastercycler Pro. The conditions were 53°C for 45 minutes; 85°C for 5 minutes; and 4°C hold. GEMs were then broken for purification of single-strand cDNA with DynaBeads MyOne Silane beads (Thermo Fisher Scientific). Amplification of cDNA was performed using the Eppendorf Mastercycler Pro (Eppendorf) with the following conditions: 98°C for 3 minutes; 11–13 cycles of 98°C for 15 seconds, 67°C for 20 seconds, and 72°C for 1 minute; 72°C for 1 minute; and 4°C hold. The cDNA product was purified with the SPRIselect Reagent Kit (0.6 × SPRI) (Beckman Coulter). Using the reagents in the Chromium Single-Cell 3′ Library Kit, indexed sequencing libraries were created by (a) fragmentation, end repair, and A-tailing; (b) SPRIselect cleanup; (c) adapter ligation; (d) postligation cleanup with SPRIselect; (e) sample index PCR; and (f) PostindexPCR cleanup. The barcoded sequencing libraries were quantified by quantitative PCR (KAPA Biosystems Library Quantification Kit for Illumina platforms). Sequencing libraries were transferred to the Duke University Center for Genomic and Computational Biology and were loaded on a Novaseq 6000 (Illumina) for sequencing.

### Analysis of ScRNA-Seq data.

The Cell Ranger v3 software (10× Genomics) was used to demultiplex cellular barcodes to produce raw 3′ end read profiles for individual cells. We then performed sequence alignment against the mm10 reference genome, with filtering, barcode counting, and unique molecular identifier counting to produce a feature-barcode matrix for each sample. Cell Ranger produced gene expression matrices. Downstream analysis was performed using R package Seurat (v3.0.1) (71). In Seurat, the data were first normalized to a log scale after basic filtering for minimum gene and cell observance frequency cut-offs (http://satijalab.org/seurat/pbmc3k_tutorial.html). Unwanted sources of variation, including the total cellular read count and mitochondrial reads, were removed using the regression method provided in the Seurat ScaleData function. Principal components were calculated using the most variably expressed genes, and the first 10 principal components were carried forward for clustering and visualization. Cells were embedded into a K-nearest neighbor graph using the FindNeighbors function and iteratively grouped with the Louvain algorithm via the FindClusters function. The t-distributed stochastic neighbor embedding (tSNE) dimensionality reduction method was used to place similar cells together in 2-dimensional space. Cluster biomarkers were identified using the FindAllMarkers function, and differentially expressed genes between clusters were identified using the Wilcoxon test (*P* ≤ 0.05 was considered statistically significant). For single-cell RNA velocity analysis, the “velocyto run10x” command was used to quantify spliced and unspliced mRNAs with *mm10* reference genome (31). The Scvelo package was then applied for analysis of RNA velocity (32). Briefly, the normalized data were used to calculate first- and second-order moments for each cell among the nearest neighbors in PCA space using scvelo.pp.moments() function. Next, the velocities were estimated and the velocity graph constructed using the scvelo.tl.velocity() and scvelo.tl.velocity_graph() functions. Velocity vectors were visualized on previously calculated tSNE coordinates with the scvelo.tl.velocity_embedding() function. The scRNA-Seq data have been deposited in the Gene Expression Omnibus (GEO, RRID:SCR_005012) under accession code GSE162847.

To identify marker genes that are highly expressed in SP cells, we filtered for genes that are upregulated in all 3 KPCC samples with the following criteria: average log fold change of ≥0.5, adjusted *P* ≤ 0.05, expressed in an average of 35% of SP cells, and upregulated in ≥60% of SP clusters. SP and non-SP clusters were identified as cell clusters enriched for SP and non-SP cells, respectively. scRNA-Seq data for mouse muscles were accessed from GSE143435_D0 and human muscles accessed from GSE130646. Analysis of cell populations and marker gene expression was performed using Seurat with a similar process as analysis of the KPCC tumor cells in this study.

### Gene ontology analysis.

To understand the molecular process associated with differentially regulated genes, genes with adjusted *P* ≤ 0.05 were divided into upregulated (log fold change > 0) and downregulated (log fold change < 0). Genes were submitted to Gene Ontology (GO) (http://geneontology.org/) for the PANTHER Overrepresentation Test (released February 24, 2021) with GO Ontology database DOI: 10.5281/zenodo.4735677 (released May 1, 2021). Fisher’s test was used for statistical significance, and GO processes with an FDR ≤ 0.05 were considered significant. GO processes with redundant key terms were reduced where only one of the GO terms was included in the tables of this study. Grouping of genes in Supplemental Tables 2–4, 6, and 8 are based on GO terms and literature review of gene function databases (72–75).

### Statistics.

Graphs and statistical calculation were generated using GraphPad Prism (RRID:SCR_002798, version 8), unless described otherwise. Error bars indicate SEM. Statistical significance was determined by 2-tailed Student’s *t* test unless described otherwise. *P* ≤ 0.05 and adjusted *P* ≤ 0.05 were considered statistically significant.

### Study approval.

All animal studies were performed in accordance with approved protocols from Duke University Institutional Animal Care and Use Committee.

## Author contributions

YJT and BAA designed the study. YJT designed and conducted the experiments with assistance from VP, YY, HZ, and PN. YJT, YX, and YD analyzed the sc-RNAseq data. DGK contributed to the project intellectually. YJT wrote the manuscript with input from the coauthors. BAA supervised the study.

## Supplementary Material

Supplemental data

## Figures and Tables

**Table 1:**
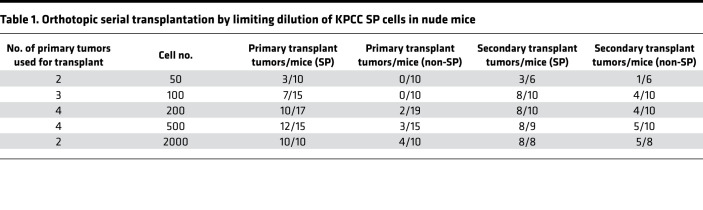
Orthotopic serial transplantation by limiting dilution of KPCC SP cells in nude mice

**Table 2:**
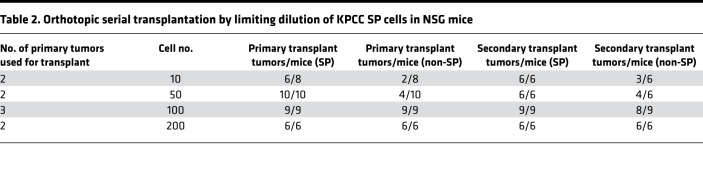
Orthotopic serial transplantation by limiting dilution of KPCC SP cells in NSG mice

**Table 3:**
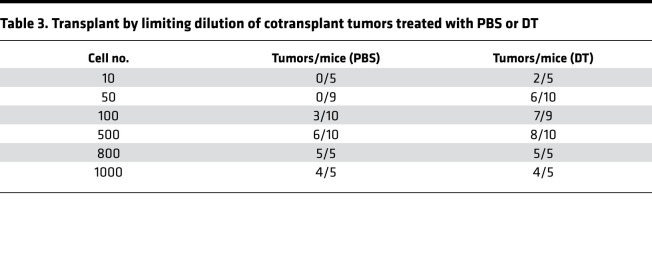
Transplant by limiting dilution of cotransplant tumors treated with PBS or DT

## Version 1 10/07/2021

In-Press Preview

## Version 2 11/22/2021

Electronic publication
