# Supplementary material for: Tumor-propagating side population cells are a dynamic subpopulation in undifferentiated pleomorphic sarcoma
Source: JCI Insight. 2021 Nov 22;6(22):e148768. doi: 10.1172/jci.insight.148768 (PMC8663789; doi:10.1172/jci.insight.148768)
Supplement: Supplemental data [file jciinsight-6-148768-s110.pdf]

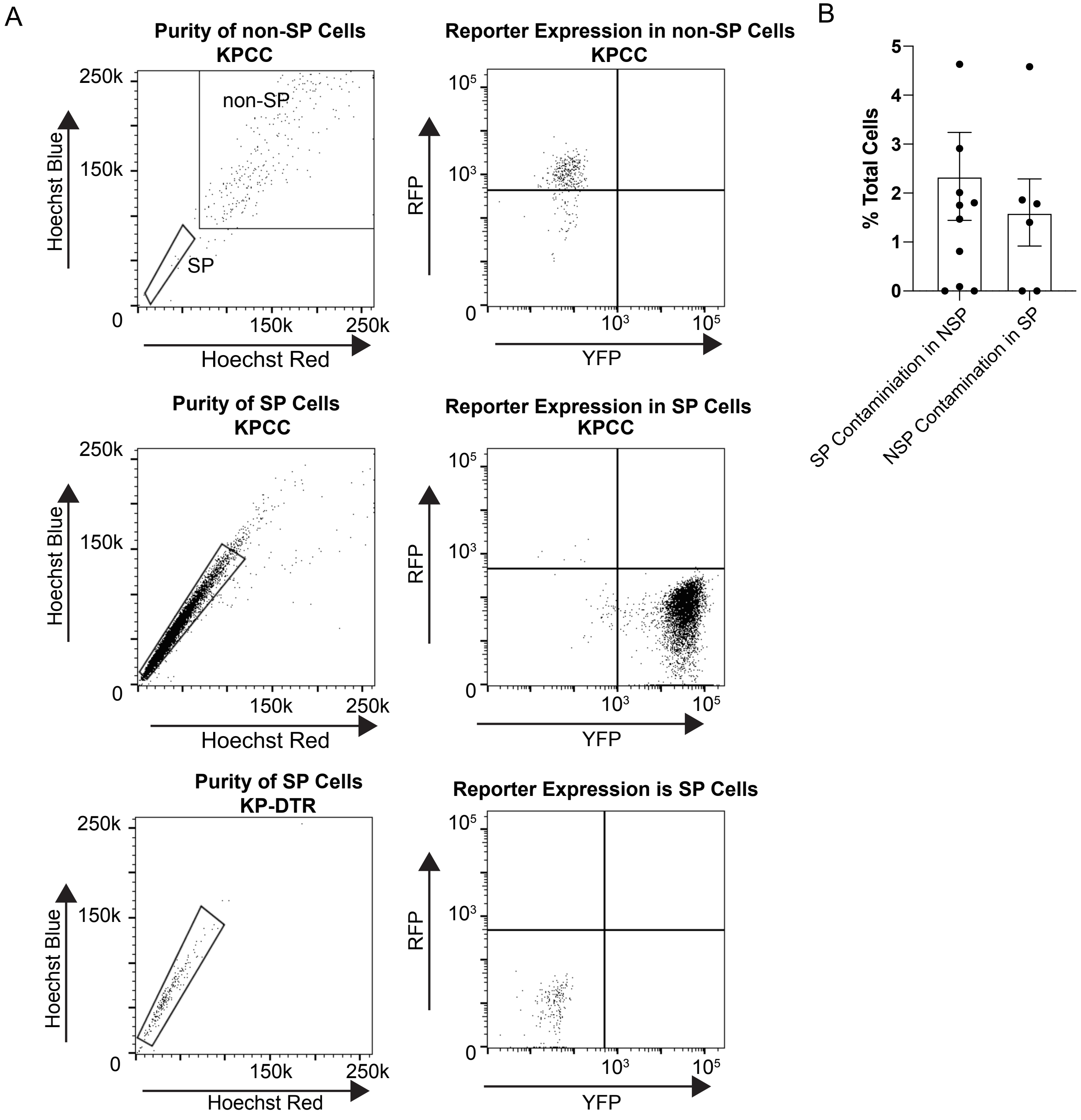

Supplemental Figure 1. Purity of sorted SP and non-SP cells by FACS. A. Representative FACS plot analyzing the purity of sorted SP and non-SP cells from KPCC tumors and the purity of sorted SP cells from KP-DTR tumors. **B.** Graph showing the mean percent of non-SP cells in sorted SP population and SP cells in sorted non-SP population with error bars that represent standard error of mean.

A

Cell Number	Tumors/Transplanted Mice (D-SP)	Tumors/Transplanted Mice (non-SP)
100	2/5	0/5
200	7/9	3/9
Total	9/14	3/14

B

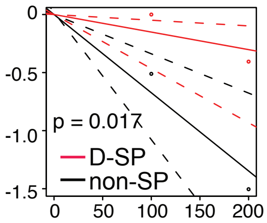

Supplemental Figure 2. **Tumors formed from serial transplantation by limiting dilution of derived SP cells from non-SP cells (D-SP) compared to non-SP cells. A.** Table showing the number of tumors formed from orthotopic transplant. **B.** ELDA analysis shows D-SP cells are enriched for tumor propagating potential compared to non-SP cells(Chi-sq = 5.7, df = 1, p = 0.017). Dotted line indicates 95% confidence intervals.

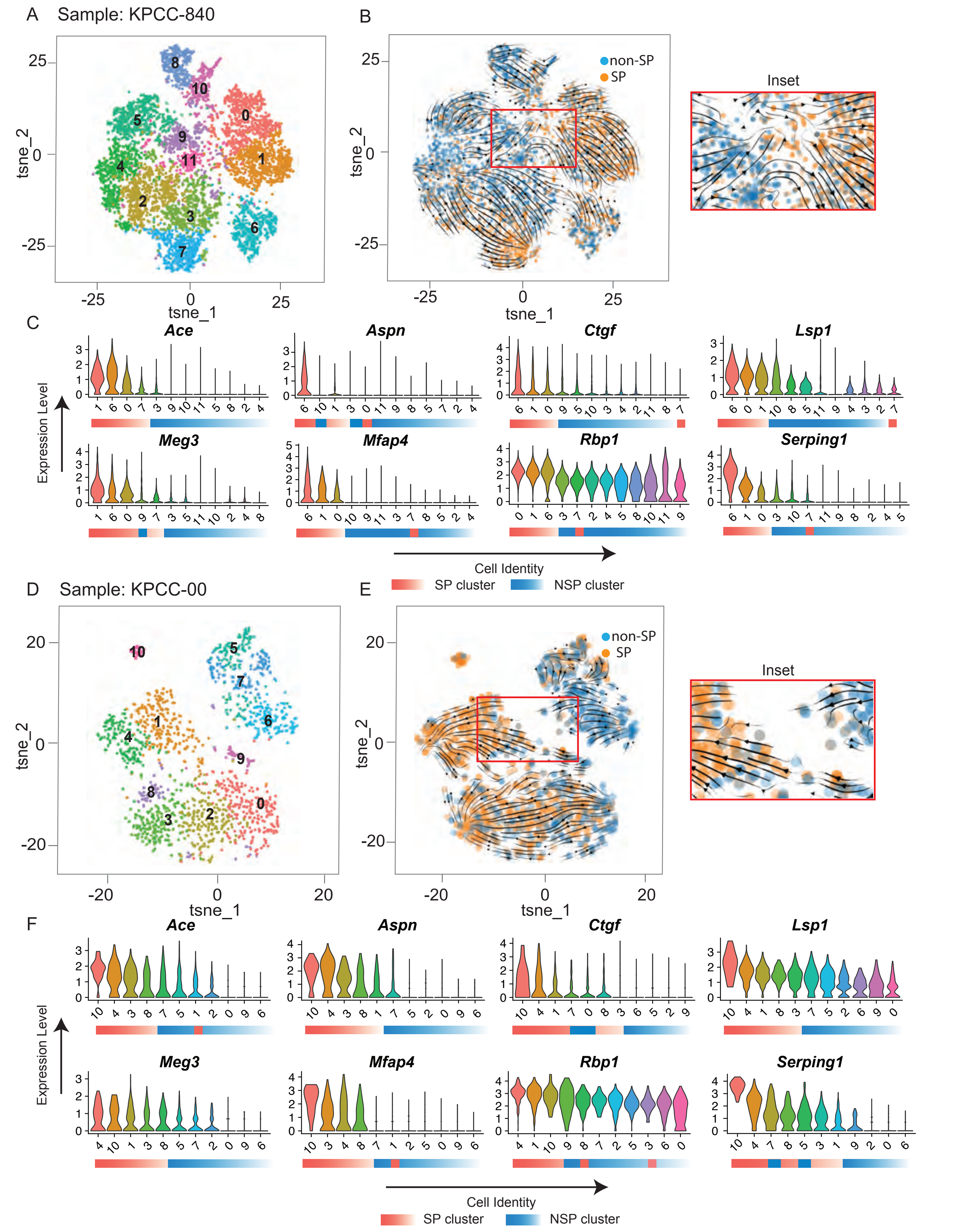

Supplemental Figure 3. ScRNA-seq and RNA velocity analysis of sorted SP and non-SP cells from KPCC tumors. A - C. Sorted SP and non-SP cells from primary autochthonous tumor KPCC-840 are analyzed by scRNA-seq. **D - F.** Sorted SP and non-SP cells from primary autochthonous tumor KPCC-00 are analyzed by scRNA-seq. The tSNE plots, overlaid RNA-velocity analyses, and violin plots of marker genes of SP cells for each tumor are shown. The cell identity number in violin plots correspond to the cell identity number in tSNE plots.

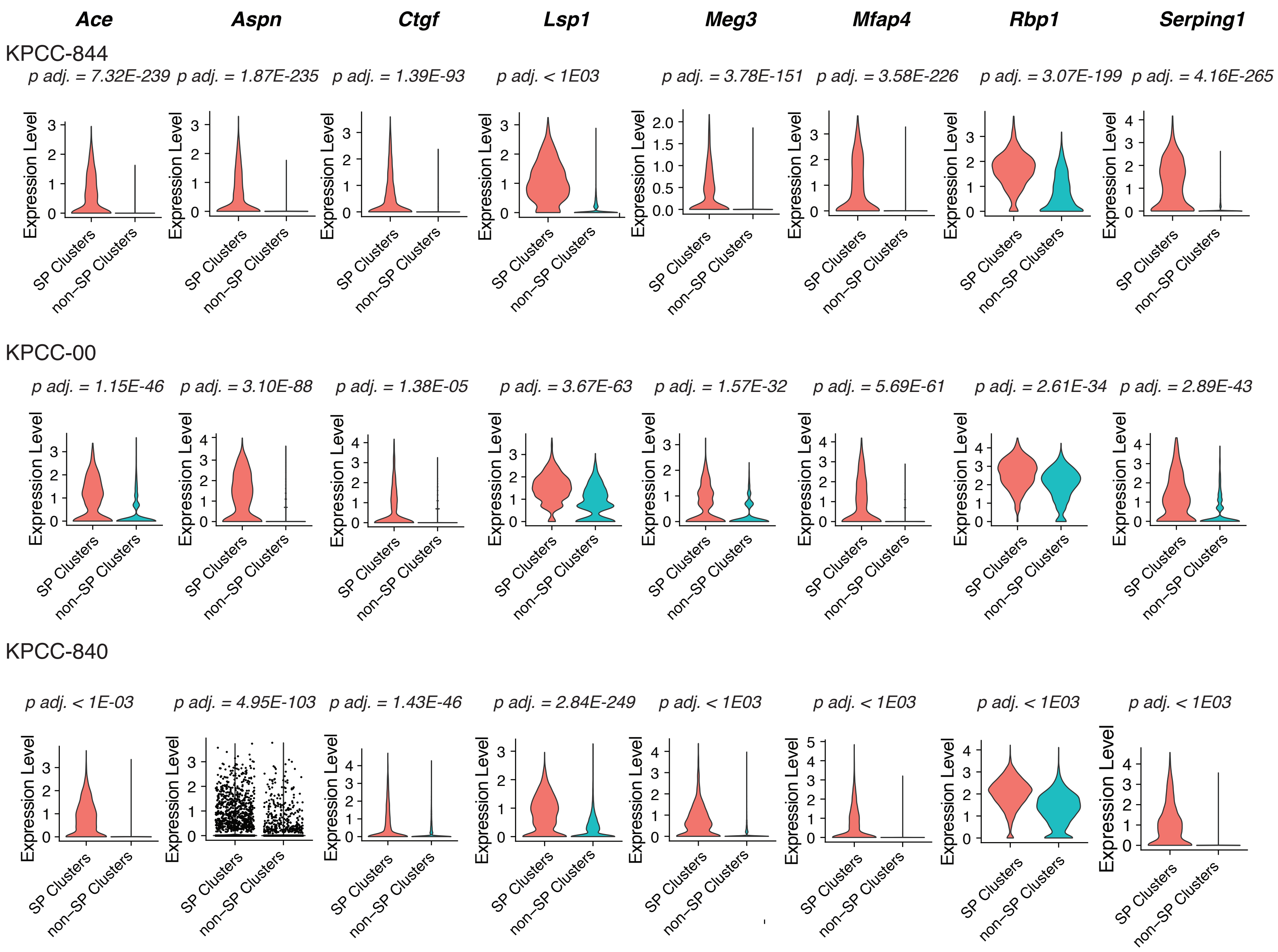

Supplemental Figure 4. Expression of SP marker genes between SP and non-SP cell clusters for KPCC tumors. Cell clusters from scRNA-seq data are grouped as SP and non-SP populations based on their enrichment for each population. Expression of SP marker genes are analyzed. Adjusted p values (p. adj) are based on Bonferroni correction using the Seurat Package.

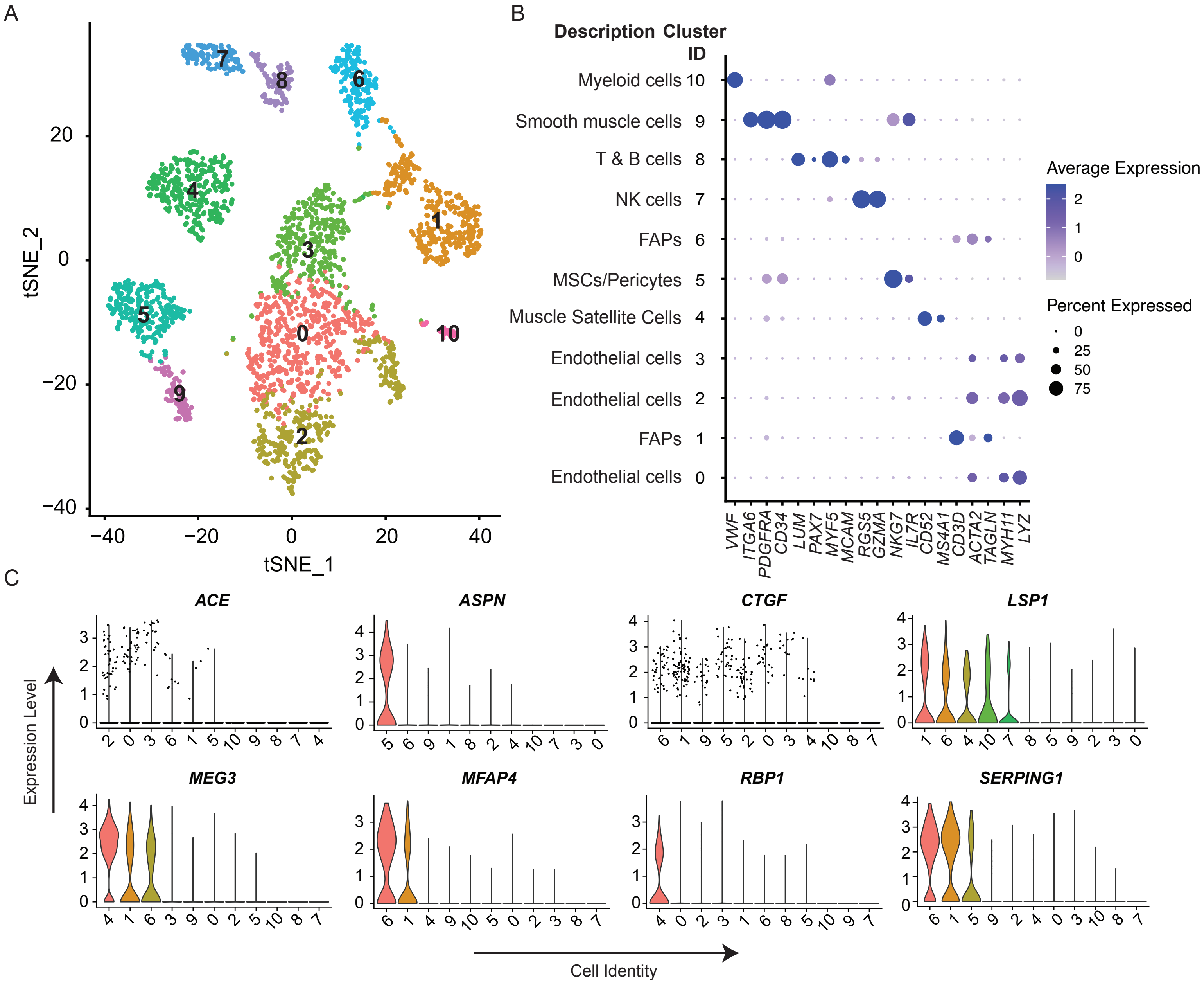

Supplemental Figure 5. ScRNA-seq analysis of normal human muscles. A. Tsne plot of different cell populations in normal human muscle. **B**. Dotplot of marker gene expression that identifies different cell clusters. **C**. Violin plot of SP marker gene expression in different subpopulations of normal human muscle cells. The cell identity number in violin plots correspond to the cell identity number in tSNE plots.

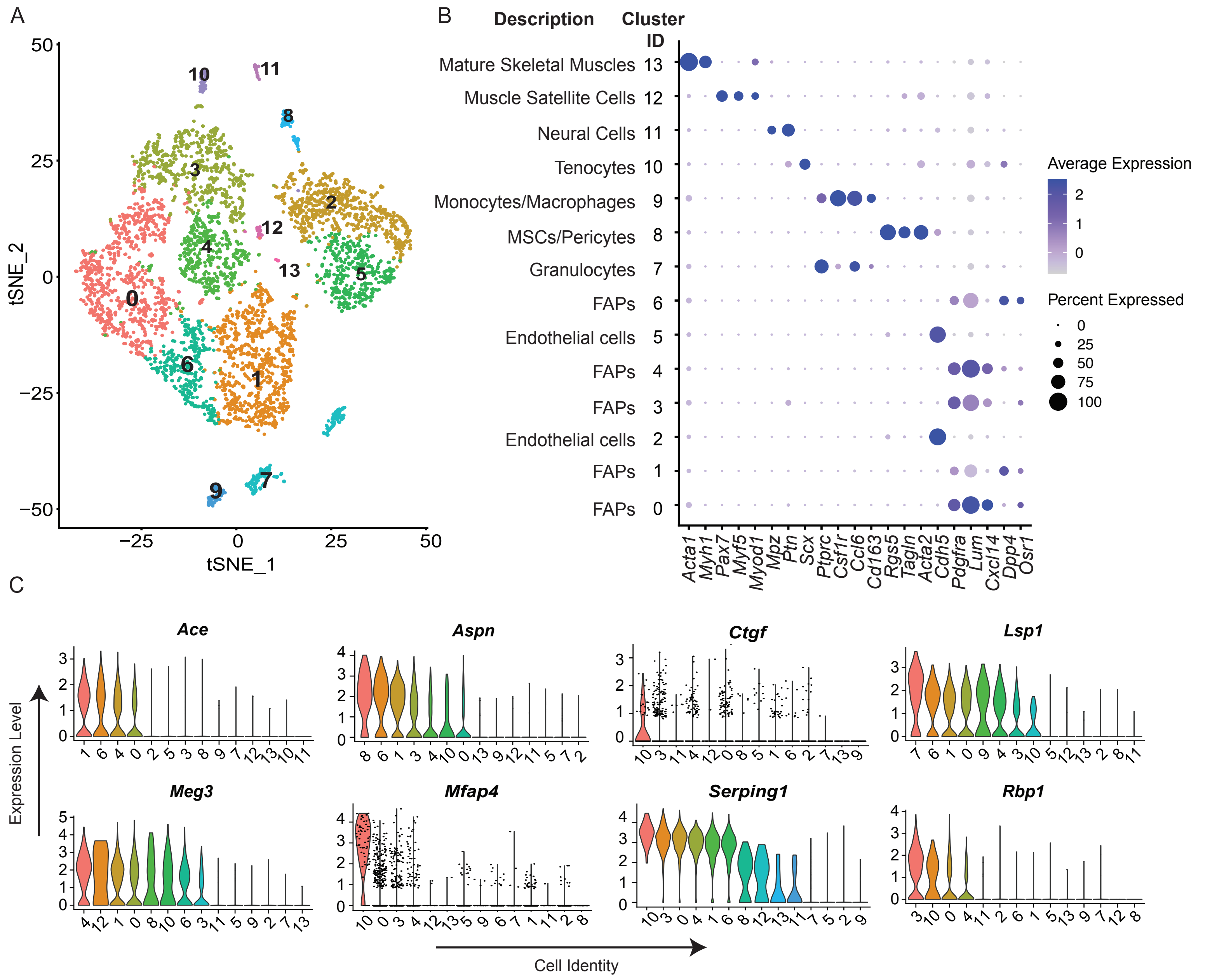

Supplemental Figure 6. ScRNA-seq analysis of normal mouse muscles. **A**. Tsne plot of different cell populations in normal mouse muscle. **B**. Dotplot of marker gene expression that identifies different cell clusters. **C**. Violin plot of SP marker gene expression in subpopulations of different normal mouse muscle cells. The cell identity number in violin plots correspond to the cell identity number in tSNE plots.

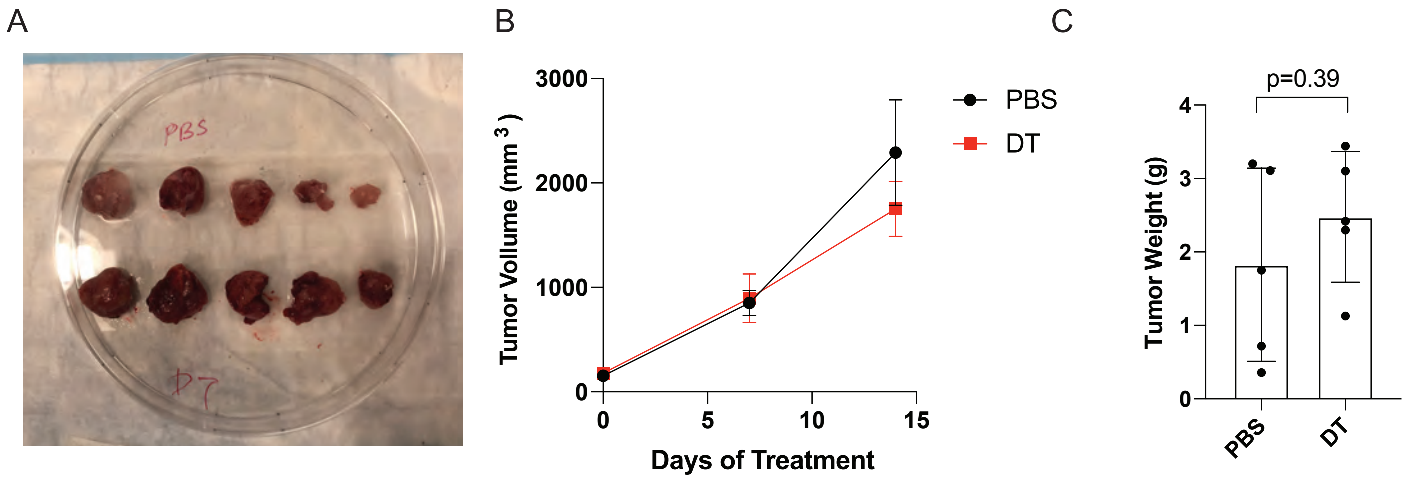

Supplemental Figure 7. SP ablation by DT treatment does not impact tumor growth. A. Representative image of PBS (top row) and DT (bottom row) treated KPCC-NSP and KP-DTR co-transplanted tumors in nude mice. **B.** Tumor volume of PBS and DT treated KPCC-NSP and KP-DTR co-transplanted tumors in nude mice. Each dot is the mean tumor volume of n = 10 mice. Error bars represent standard error of mean. **C.** Tumor weight of PBS and DT treated KPCC-NSP and KP-DTR co-transplanted tumors in nude mice. P value determined using two-tailed Student's t-test. Error bars indicate standard error of mean.

Supplementary Table 1. Differential Gene Expression Analysis from scRNA-seq of SP relative to non-SP cells in tumor KPCC-844

	Function Group	Gene	p value	average logFC	pct.1	pct.2	adusted p value
Upregulated genes	Skeletal muscle development	Myl6	1.97E-64	0.283079746	1	1	3.66E-60
		Chrnd	8.71E-58	0.297508602	0.138	0.013	1.62E-53
		Vgll2	7.63E-61	0.281481707	0.113	0.002	1.42E-56
		Maff	3.13E-20	0.285785697	0.877	0.799	5.81E-16
		Nr4a1	1.58E-31	0.505790306	0.685	0.538	2.94E-27
		Fos	1.96E-59	0.640015618	0.938	0.834	3.63E-55
		Mylpf	9.66E-12	0.47304645	0.149	0.084	1.79E-07
		Acta1	4.74E-08	0.365508259	0.13	0.2	8.79E-04
		Myod1	2.68E-83	0.817947469	0.163	0.006	4.96E-79
		Dcn	2.89E-114	1.350517456	0.878	0.713	5.36E-110
		Ankrd1	8.74E-48	1.283459539	0.29	0.121	1.62E-43
		Eln	6.12E-40	0.254705985	0.13	0.025	1.13E-35
		Heyl	4.86E-36	0.253694686	0.113	0.02	9.02E-32
	Negative regulation of developmental processes	Tgfbr2	2.88E-27	0.347782024	0.7	0.622	5.34E-23
		Col5a1	1.61E-126	1.114973244	0.67	0.405	2.99E-122
		Tmem176a	0	1.534429706	0.627	0.071	0
		Serpinf1	3.17E-47	0.341057186	0.989	0.98	5.87E-43
		Emilin1	3.01E-26	0.314095668	0.374	0.244	5.58E-22
		Spry1	5.93E-20	0.307636358	0.329	0.217	1.10E-15
		Nppc	5.27E-51	1.33170774	0.266	0.095	9.77E-47
		Xbp1	1.67E-46	0.371056709	0.878	0.783	3.10E-42
		Zfp36l1	4.01E-63	0.603332615	0.819	0.697	7.43E-59
		Postn	0	2.733850662	0.756	0.133	0
		Adamts1	1.92E-18	0.39808701	0.668	0.597	3.55E-14
		Serpine1	1.68E-61	0.985300778	0.587	0.394	3.12E-57
		Igf2	1.04E-76	0.834385905	0.156	0.007	1.92E-72
		Rgcc	2.87E-73	0.819980243	0.734	0.504	5.32E-69
		Gnas	2.21E-116	0.322526504	1	1	4.09E-112
		Gas6	8.45E-64	0.373979095	0.172	0.023	1.57E-59
		Thy1	1.82E-28	0.317089177	0.878	0.769	3.38E-24
		Ltbp3	1.02E-59	0.360057876	0.368	0.158	1.89E-55
		Cxcl14	0	3.07933089	0.888	0.208	0
		Gdf15	6.27E-10	0.499749294	0.267	0.198	1.16E-05
		Ifrd1	2.63E-14	0.285375066	0.991	0.995	4.87E-10
		Tmem119	1.56E-62	0.564536758	0.375	0.158	2.90E-58
		Ccl2	3.74E-137	1.736662156	0.933	0.677	6.93E-133
		Aspn	2.09E-135	1.064082419	0.35	0.054	3.88E-131
		Stat3	5.99E-38	0.293248549	0.768	0.67	1.11E-33
		Mmp9	4.02E-17	0.380690495	0.154	0.074	7.45E-13
		Enpp1	1.40E-34	0.391422943	0.475	0.314	2.59E-30
		Fbn1	3.81E-157	1.50496152	0.774	0.515	7.07E-153
		Noct	4.48E-09	0.256783676	0.477	0.441	8.30E-05
		Fndc3b	3.93E-30	0.276420189	0.761	0.657	7.29E-26
		Acvrl1	2.57E-87	0.304523858	0.22	0.026	4.76E-83
		Snai2	7.96E-17	0.254602331	0.346	0.247	1.48E-12
		Rac3	2.28E-43	0.303622842	0.336	0.16	4.22E-39
		Ptn	3.23E-40	0.692124358	0.425	0.249	6.00E-36
		Kctd11	5.66E-19	0.33588973	0.381	0.274	1.05E-14
		Osr1	2.98E-56	0.339144282	0.234	0.063	5.52E-52
		Pgk1	1.27E-27	0.318900101	0.634	0.546	2.36E-23
		Tmem176b	0	1.767933981	0.664	0.079	0
		Igf1	4.60E-149	1.228563671	0.351	0.043	8.54E-145
		Igfbp5	6.20E-86	0.987331138	0.286	0.06	1.15E-81
		Ecscr	6.56E-44	0.523453368	0.504	0.327	1.22E-39
		Zfp36	1.15E-12	0.439111098	0.798	0.72	2.13E-08
		Fbln5	3.13E-86	0.608320008	0.405	0.15	5.80E-82
		Nfib	1.98E-25	0.314767652	0.611	0.531	3.68E-21
		Sfrp1	1.06E-93	0.720930464	0.204	0.015	1.97E-89
		Lrrc17	7.17E-78	0.477002916	0.334	0.105	1.33E-73
		Col4a2	1.66E-22	0.527922686	0.73	0.672	3.07E-18
		Rgs2	3.19E-07	0.262981238	0.708	0.659	0.005917559
		Hspb1	2.85E-08	0.501738386	0.981	0.975	5.28E-04
		Sulf1	1.56E-196	0.829078934	0.576	0.152	2.89E-192
		Kremen1	2.04E-38	0.261042967	0.318	0.155	3.79E-34
		Rora	9.03E-56	0.428378744	0.511	0.294	1.67E-51
		Nrp1	1.23E-144	1.038899257	0.678	0.365	2.27E-140
		Il4ra	4.47E-57	0.456160013	0.45	0.247	8.29E-53
		Thbs1	3.27E-22	0.353056172	0.826	0.715	6.06E-18
		Creb3l1	4.27E-59	0.289532218	0.267	0.08	7.92E-55
		Mafb	1.70E-82	0.573809077	0.268	0.056	3.15E-78
		Tgfbr3	3.65E-63	0.477899153	0.465	0.248	6.76E-59
		Klf2	5.25E-28	0.373147937	0.272	0.143	9.74E-24
		Tpt1	4.68E-100	0.261473323	1	1	8.68E-96
		Dusp10	2.49E-12	0.266072163	0.476	0.4	4.61E-08
		Ccl11	3.36E-215	2.201638666	0.415	0.027	6.23E-211
		Wisp1	1.57E-43	0.397876831	0.563	0.378	2.91E-39
		Nucb2	2.74E-34	0.359864643	0.781	0.716	5.08E-30
		Lif	7.40E-10	0.280109621	0.145	0.086	1.37E-05
		Plac8	3.41E-129	1.615860224	0.513	0.197	6.33E-125
		Sfrp2	3.09E-102	0.947876032	0.293	0.051	5.73E-98
		Arhgef2	2.00E-12	0.350534042	0.574	0.558	3.72E-08
		App	9.29E-115	0.592981786	0.95	0.873	1.72E-110
		Il6	3.29E-103	1.10034302	0.296	0.051	6.10E-99
		Ngp	1.20E-90	0.757709018	0.267	0.046	2.23E-86

		Ypel3	4.57E-102	0.656040824	0.731	0.486	8.47E-98
		Ldlrad4	1.98E-47	0.268629408	0.301	0.124	3.68E-43
		Ybx1	3.13E-131	0.432959808	1	1	5.81E-127
		Rbp4	2.31E-59	0.5535972	0.465	0.237	4.28E-55
		Loxl2	6.43E-75	0.702410199	0.864	0.743	1.19E-70
		Sparc	8.70E-102	1.155677568	0.999	0.995	1.61E-97
		Tcf7l2	2.02E-20	0.284721529	0.491	0.396	3.74E-16
		Loxl3	1.18E-190	1.392025226	0.664	0.276	2.18E-186
		Hes1	2.44E-28	0.341477936	0.276	0.144	4.53E-24
		Ctgf	8.25E-60	0.992992893	0.397	0.187	1.53E-55
		Hif1a	2.04E-44	0.397288464	0.874	0.802	3.78E-40
		Npdc1	1.45E-87	0.578940389	0.764	0.569	2.69E-83
		Col5a2	1.69E-207	1.674674071	0.824	0.499	3.13E-203
		Klf9	1.91E-50	0.44678124	0.638	0.484	3.54E-46
		Ddit4	2.75E-21	0.582394679	0.621	0.528	5.10E-17
		Cebpd	2.71E-21	0.387164601	0.86	0.753	5.03E-17
		Cd248	8.07E-72	0.514404296	0.324	0.107	1.50E-67
		Tagln	1.84E-90	1.103136533	0.26	0.044	3.40E-86
		Fhl1	1.37E-152	0.695004169	0.413	0.076	2.53E-148
		Lamp1	1.53E-48	0.272832165	0.996	0.98	2.84E-44
		Sgcb	9.62E-94	0.484472145	0.5	0.225	1.78E-89
		Tppp3	6.48E-64	0.644028556	0.299	0.101	1.20E-59
		Tubb2a	6.62E-26	0.387667329	0.818	0.739	1.23E-21
		Des	1.37E-113	1.789781019	0.228	0.012	2.53E-109
		Ogn	4.47E-106	0.566551742	0.28	0.041	8.30E-102
		Vcan	2.73E-24	0.344093086	0.83	0.786	5.07E-20
		Cyr61	3.47E-34	0.813330168	0.969	0.932	6.44E-30
		Rab13	2.51E-30	0.27725262	0.703	0.619	4.65E-26
		Pbxip1	3.69E-69	0.508476483	0.631	0.424	6.84E-65
		Prg4	4.06E-66	0.976987673	0.252	0.063	7.53E-62
		Bhlhe40	5.20E-14	0.30644075	0.468	0.39	9.65E-10
		Igsf3	5.42E-30	0.266271853	0.364	0.214	1.01E-25
		Crip2	2.75E-33	0.965840304	0.478	0.347	5.11E-29
		Itm2c	1.51E-56	0.323366694	0.987	0.969	2.79E-52
		Ebf1	2.66E-32	0.416667638	0.753	0.699	4.93E-28
		AW551984	5.31E-79	0.304607031	0.212	0.03	9.85E-75
		Btf3	9.90E-91	0.268933021	1	1	1.84E-86
	Developmental process	Mxra8	1.98E-96	0.719121125	0.819	0.663	3.67E-92
		Nxn	4.02E-41	0.35352418	0.719	0.613	7.46E-37
		Steap4	5.67E-65	0.36353984	0.214	0.043	1.05E-60
		Igfbp7	0	2.688348523	0.623	0.044	0
		Bicc1	3.13E-146	0.659904884	0.425	0.091	5.81E-142
		Lama4	4.13E-121	0.688733734	0.458	0.141	7.67E-117
		Ifi27l2a	2.21E-26	0.419736258	0.137	0.044	4.09E-22
		Pld3	2.68E-53	0.363009051	0.866	0.759	4.96E-49
		Medag	7.19E-169	0.962667405	0.455	0.09	1.33E-164
		Sparcl1	3.26E-38	0.915925185	0.553	0.401	6.05E-34
		Ifi27	9.64E-128	0.785276163	0.854	0.672	1.79E-123
		Nrep	7.20E-34	0.695061742	0.415	0.266	1.33E-29
		Slc38a10	1.66E-31	0.280771357	0.742	0.665	3.08E-27
		Tacc1	1.83E-51	0.302198702	0.368	0.171	3.39E-47
		Ssc5d	5.82E-82	0.350130293	0.29	0.07	1.08E-77
		Rcn1	3.11E-99	0.465943966	0.974	0.933	5.77E-95
		Lhfpl2	2.76E-75	0.451108185	0.391	0.149	5.12E-71
		Palld	2.62E-18	0.252300386	0.709	0.639	4.86E-14
		Ift20	1.37E-29	0.252136073	0.981	0.961	2.54E-25
		Pi15	3.39E-22	0.393265492	0.236	0.128	6.29E-18
		Txnip	1.33E-69	0.732083786	0.266	0.069	2.46E-65
		Tubb2b	2.99E-19	0.339040872	0.77	0.697	5.55E-15
		Marcks	2.71E-38	0.35326556	0.71	0.597	5.03E-34
		C3	5.69E-60	0.343699957	0.146	0.015	1.05E-55
		Yipf3	2.23E-34	0.259695794	0.953	0.925	4.13E-30
		Tmsb10	0	1.151479972	0.999	1	0
		Emp1	1.68E-45	0.361564299	0.993	0.987	3.12E-41
		Cst3	9.49E-38	0.372422097	1	0.999	1.76E-33
		Ndufs5	1.24E-84	0.408446999	0.758	0.583	2.30E-80
		Ndufb5	3.79E-29	0.255273116	0.999	0.996	7.02E-25
		Ltbp2	2.96E-123	0.581634488	0.298	0.035	5.49E-119
		Cnn3	8.16E-32	0.382299673	0.987	0.982	1.51E-27
		Itgb5	5.93E-37	0.36481214	0.458	0.303	1.10E-32
		Tmed3	2.63E-21	0.250189954	0.992	0.993	4.87E-17
		Rpl35	4.51E-120	0.252491367	1	1	8.37E-116
	Cellular organization or biogenesis	Mgarp	4.28E-40	0.291058213	0.222	0.078	7.94E-36
		Tap2	2.07E-131	0.430192283	0.348	0.053	3.83E-127
		Cox7a2l	2.56E-117	0.435200869	0.992	0.956	4.75E-113
		Tspan4	3.37E-73	0.516932404	0.954	0.923	6.26E-69
		Cotl1	4.20E-15	0.395013268	0.559	0.494	7.79E-11
		Rps27	6.70E-288	0.535397005	1	1	1.24E-283
		Gpc4	4.40E-33	0.33904985	0.672	0.539	8.15E-29
		Gem	3.50E-53	0.654982113	0.744	0.569	6.49E-49
		H2-K1	1.86E-51	0.378365157	0.877	0.684	3.44E-47
		Cd302	2.53E-131	0.698429346	0.755	0.498	4.69E-127
		Tmem45a	2.99E-86	0.664860715	0.429	0.165	5.55E-82
		Tln2	2.57E-75	0.481336331	0.388	0.155	4.77E-71
		Dcbld2	2.36E-24	0.275219658	0.487	0.372	4.38E-20
		Col1a1	3.18E-236	2.40723793	0.806	0.384	5.91E-232

		Mme	5.34E-61	0.295747908	0.147	0.014	9.90E-57
		Pros1	3.83E-81	0.582474345	0.8	0.628	7.10E-77
		Fkbp10	2.44E-34	0.315380886	0.961	0.967	4.52E-30
		Ppia	1.21E-178	0.497866817	1	1	2.24E-174
		Lox	2.35E-260	1.545955636	0.672	0.177	4.36E-256
		Dag1	3.91E-20	0.346467871	0.591	0.507	7.26E-16
		Ptk7	4.20E-157	0.7201388	0.461	0.104	7.79E-153
		Myl9	1.36E-97	1.0009585	0.349	0.089	2.52E-93
		Pdpn	1.88E-30	0.263664673	0.971	0.934	3.49E-26
		Col1a2	6.20E-139	1.690844865	0.922	0.813	1.15E-134
	Wound healing	Fcer1g	9.89E-34	0.967642338	0.279	0.139	1.83E-29
		Serpine2	1.52E-123	1.269362169	0.666	0.341	2.82E-119
		Tgfb3	1.70E-15	0.34981513	0.457	0.383	3.15E-11
		Col3a1	6.93E-100	1.563653232	0.975	0.904	1.28E-95
		S100a8	1.92E-145	1.151083007	0.613	0.224	3.55E-141
		Tfpi2	1.84E-84	0.848543466	0.599	0.36	3.42E-80
		Hmox1	1.97E-54	0.761582746	0.948	0.859	3.65E-50
		Eng	6.51E-29	0.394549894	0.436	0.314	1.21E-24
		Fn1	1.87E-216	1.315760636	0.98	0.857	3.46E-212
		Timp1	8.94E-30	0.509572244	0.999	0.999	1.66E-25
		Tnc	1.53E-183	1.740480612	0.883	0.57	2.85E-179
		Csrp1	2.07E-81	0.649628652	0.899	0.779	3.84E-77
		Serping1	2.52E-192	1.562307952	0.619	0.206	4.67E-188
		Lbp	4.34E-74	0.340357882	0.163	0.011	8.04E-70
		Il1rl1	4.50E-30	0.61598858	0.373	0.229	8.34E-26
		C1qtnf3	1.03E-75	0.726505751	0.148	0.005	1.91E-71
	Chemokine production	Apod	1.86E-25	0.707934117	0.372	0.24	3.46E-21
		Gstp1	9.71E-89	0.413833514	0.828	0.688	1.80E-84
		Lgals9	3.07E-46	0.324363488	0.32	0.14	5.70E-42
		Lrp1	3.89E-106	0.845477402	0.902	0.824	7.22E-102
		Tnfaip3	8.42E-30	0.42722953	0.362	0.218	1.56E-25
		Casp4	1.04E-44	0.306915931	0.432	0.231	1.94E-40
		Clip3	2.33E-56	0.345708664	0.481	0.262	4.33E-52
		Csf1	8.21E-62	0.646580832	0.639	0.463	1.52E-57
		Hspa1a	2.35E-19	0.526368647	0.984	0.978	4.36E-15
		Il1r1	4.72E-47	0.442165371	0.541	0.366	8.76E-43
		Slit3	1.87E-119	0.502876967	0.333	0.058	3.47E-115
		Sphk1	1.42E-63	0.53543749	0.541	0.329	2.63E-59
	Cytokine mediated signaling	Socs3	2.54E-09	0.344734895	0.555	0.533	4.72E-05
		Ifngr1	5.35E-53	0.444165093	0.696	0.545	9.91E-49
		Angpt1	1.01E-56	0.258441581	0.248	0.071	1.88E-52
		Hspa1b	8.17E-73	0.790246432	0.889	0.767	1.52E-68
		Jak1	7.82E-60	0.356838507	0.871	0.756	1.45E-55
		Irf7	1.27E-09	0.268679336	0.156	0.095	2.36E-05
		Zbp1	5.57E-71	0.427849189	0.324	0.101	1.03E-66
		Nol3	4.94E-59	0.344153605	0.301	0.107	9.17E-55
		Il6st	2.11E-50	0.410627052	0.609	0.449	3.91E-46
		Mdfic	4.08E-57	0.374762597	0.509	0.298	7.56E-53
		Gadd45b	1.16E-118	0.936472884	0.909	0.753	2.15E-114
		Dnaja1	2.80E-20	0.323380414	0.975	0.961	5.18E-16
		Ubc	1.88E-35	0.392895887	0.979	0.955	3.49E-31
	MAP kinase activity	Uba52	1.12E-156	0.507307153	0.993	0.95	2.08E-152
		Tnfsf11	3.74E-95	0.687194789	0.334	0.084	6.94E-91
		Ubb	1.22E-246	0.627925548	1	1	2.26E-242
		Akap13	1.87E-23	0.286867085	0.492	0.388	3.46E-19
		Fam195b	4.41E-57	0.326199508	0.974	0.956	8.18E-53
		Igf1r	2.07E-18	0.29368301	0.494	0.412	3.83E-14
	Response to IL-15	Il15ra	1.10E-91	0.4623785	0.277	0.052	2.04E-87
		Acsl4	1.07E-71	0.445488283	0.716	0.533	1.98E-67
		Ptgs2	7.73E-29	0.657751903	0.697	0.579	1.43E-24
		Lrrc32	2.62E-25	0.279506122	0.27	0.147	4.85E-21
		Cd200	5.67E-40	0.752977116	0.547	0.418	1.05E-35
	Negative regulation of transforming growth factor beta signaling pathway	Lum	1.14E-30	0.684881389	0.623	0.463	2.11E-26
		Htra1	2.00E-14	0.294323474	0.919	0.864	3.71E-10
		Hspa5	4.32E-15	0.293181105	0.999	1	8.00E-11
		Pmepa1	8.72E-80	0.718686909	0.84	0.734	1.62E-75
		Htra3	1.63E-64	0.354124585	0.183	0.027	3.03E-60
		Bst2	4.68E-37	0.420937566	0.585	0.419	8.68E-33
		Cxcl1	7.19E-87	1.25838628	0.986	0.842	1.33E-82
		Cxcl16	2.01E-67	0.42769187	0.33	0.113	3.73E-63
		Osmr	2.56E-91	0.394359953	0.406	0.135	4.75E-87
		Numbl	4.13E-35	0.290753957	0.489	0.339	7.67E-31
		Cxcl3	1.35E-10	0.691234949	0.186	0.117	2.50E-06
		Ccl8	8.49E-41	0.706557854	0.178	0.051	1.57E-36
		Ndufa13	7.72E-64	0.254875724	1	1	1.43E-59
		Ifi205	9.26E-75	0.347518485	0.172	0.015	1.72E-70
		Tnfrsf1a	2.75E-48	0.367381712	0.927	0.866	5.09E-44
		Ccl7	1.65E-176	1.769202155	0.868	0.496	3.05E-172
		Nfkb1	1.06E-21	0.301688351	0.561	0.471	1.96E-17
		Nfkbiz	1.08E-26	0.350251617	0.725	0.613	2.00E-22
		Tnfaip6	7.14E-83	1.096644374	0.766	0.561	1.32E-78
		Aldh1a2	3.78E-83	0.793704061	0.2	0.021	7.01E-79
		Nfil3	4.89E-31	0.356163265	0.507	0.369	9.07E-27
		Gbp2	1.48E-48	0.322611109	0.213	0.059	2.75E-44
		Cfh	9.15E-80	0.289798274	0.167	0.009	1.70E-75
	Response to cytokine	Serpina3g	3.01E-48	0.276679169	0.152	0.027	5.58E-44

		Ugcg	8.45E-98	0.633933724	0.561	0.293	1.57E-93
		Dok1	6.31E-35	0.483321058	0.774	0.669	1.17E-30
		Cxcl5	1.42E-191	2.056857582	0.381	0.026	2.64E-187
		Il3ra	1.63E-41	0.34868995	0.658	0.523	3.01E-37
		Ifi204	6.21E-29	0.323660204	0.458	0.323	1.15E-24
		Hk2	2.11E-26	0.345882959	0.656	0.578	3.91E-22
		Timp2	2.76E-76	0.490125916	0.962	0.906	5.12E-72
		Gfpt2	1.81E-21	0.325742717	0.464	0.353	3.36E-17
		Iigp1	7.42E-49	0.465427315	0.109	0.008	1.38E-44
		Il11ra1	1.69E-28	0.461230631	0.605	0.521	3.14E-24
		Serpina3n	1.26E-53	0.511438507	0.17	0.032	2.33E-49
		Gapdh	2.81E-82	0.307870926	1	1	5.21E-78
		Cxcl2	1.27E-15	0.558617533	0.891	0.771	2.35E-11
		Isg15	4.26E-35	0.534253272	0.321	0.157	7.91E-31
		Lsp1	3.79E-254	1.430940484	0.727	0.269	7.03E-250
		Crlf1	3.57E-213	1.844450958	0.527	0.099	6.62E-209
		Pcolce	2.38E-10	0.308365569	0.984	0.993	4.41E-06
		Icam1	7.25E-34	0.343252409	0.216	0.083	1.34E-29
		Mmp3	5.25E-25	0.5752665	0.535	0.379	9.75E-21
		Plod3	2.00E-47	0.30844023	0.858	0.778	3.70E-43
		P3h1	1.37E-33	0.337760733	0.572	0.454	2.54E-29
		Adamts2	1.85E-200	0.860457626	0.477	0.075	3.42E-196
		Mmp19	8.56E-65	0.535272721	0.434	0.206	1.59E-60
	Collagen metabolic process	P3h3	1.03E-48	0.270295042	0.296	0.117	1.91E-44
		Mmp23	1.72E-110	0.668986036	0.473	0.174	3.19E-106
		Rcn3	1.18E-190	1.332967899	0.834	0.543	2.20E-186
		Mmp2	6.45E-48	0.746746779	0.911	0.869	1.20E-43
		Mrc2	2.60E-25	0.368241215	0.606	0.547	4.83E-21
		Serpinh1	2.17E-47	0.485763351	0.999	1	4.03E-43
		Nid1	2.88E-70	0.570102275	0.775	0.642	5.34E-66
		Col15a1	8.32E-130	1.281139722	0.413	0.101	1.54E-125
		Gpm6b	2.57E-39	0.363636913	0.371	0.203	4.77E-35
		Loxl1	9.36E-202	1.3034966	0.775	0.405	1.74E-197
		Pmp22	1.59E-39	0.443934178	0.568	0.41	2.94E-35
		Adamts4	5.45E-22	0.283517257	0.273	0.159	1.01E-17
		Bcl3	1.59E-31	0.28100513	0.404	0.251	2.95E-27
		Col16a1	8.14E-41	0.358296381	0.437	0.267	1.51E-36
		Cyp1b1	8.66E-68	0.288858895	0.134	0.005	1.61E-63
		Col4a1	2.22E-22	0.494901118	0.697	0.633	4.12E-18
		Abi3bp	3.71E-70	0.484403194	0.154	0.011	6.87E-66
		Crispld2	1.29E-15	0.294254829	0.296	0.199	2.39E-11
		Lgals3bp	5.65E-38	0.475388213	0.581	0.459	1.05E-33
		Adamts5	6.12E-97	0.448839627	0.268	0.042	1.13E-92
		Col5a3	1.41E-134	1.066035842	0.552	0.22	2.61E-130
	Extracellular matrix organization	Efemp2	5.34E-42	0.43078511	0.788	0.73	9.90E-38
		Lamc1	3.40E-23	0.309904829	0.648	0.58	6.31E-19
		Col11a1	4.93E-51	0.36874087	0.224	0.065	9.13E-47
		Fbln2	1.78E-233	1.478706986	0.718	0.271	3.30E-229
		Smoc2	7.62E-56	1.196233596	0.445	0.252	1.41E-51
		Col14a1	2.37E-55	0.323502289	0.121	0.008	4.39E-51
		Ptx3	1.33E-124	1.423235682	0.395	0.091	2.47E-120
		Ccdc80	1.18E-201	0.919538011	0.419	0.039	2.18E-197
		Emilin2	1.39E-23	0.281942631	0.465	0.337	2.57E-19
		Olfml3	1.96E-21	0.375148741	0.652	0.582	3.63E-17
		Sgce	5.04E-20	0.255931229	0.611	0.533	9.35E-16
		Dpt	6.42E-61	0.749143251	0.387	0.18	1.19E-56
		Mfap4	2.14E-152	1.398664086	0.473	0.115	3.97E-148
		Col8a1	2.70E-176	1.251065067	0.487	0.105	5.02E-172
		Crtap	6.00E-24	0.269534009	0.768	0.706	1.11E-19
		Col6a2	3.07E-171	1.640817725	0.653	0.288	5.70E-167
		Col6a1	9.00E-117	1.409209944	0.768	0.562	1.67E-112
		Fzd1	3.31E-23	0.319082376	0.41	0.294	6.13E-19
		Ednra	6.62E-47	0.340702358	0.3	0.125	1.23E-42
		Gng11	1.69E-15	0.343672147	0.971	0.955	3.13E-11
	G-protein coupled receptor signaling	Gpr180	2.97E-19	0.257326408	0.562	0.482	5.50E-15
		Mrgprf	3.68E-24	0.264602197	0.296	0.171	6.82E-20
		Pth1r	4.15E-72	0.318406522	0.233	0.047	7.70E-68
		Gng5	2.44E-134	0.405567195	1	1	4.52E-130
		Ccl3	6.94E-10	0.302534861	0.143	0.081	1.29E-05
		Kdelr2	7.93E-40	0.311156	0.989	0.983	1.47E-35
		Ppp1r15a	6.16E-50	0.40464374	0.945	0.873	1.14E-45
		Kdelr3	3.84E-31	0.311380369	0.881	0.815	7.12E-27
		Os9	7.64E-41	0.321516502	0.742	0.628	1.42E-36
	Protein localization to endoplasmic reticulum	Herpud1	1.24E-48	0.579581977	0.804	0.694	2.30E-44
		Arxes2	3.99E-55	0.30814681	0.312	0.118	7.40E-51
		Sec62	3.56E-47	0.254564002	0.997	0.992	6.61E-43
		Kdelr1	3.68E-54	0.288691175	0.994	0.992	6.82E-50
		Sec61g	6.28E-124	0.448620258	0.971	0.883	1.17E-119
		Ptgfrn	1.31E-53	0.405316525	0.553	0.364	2.43E-49
		Rpl24	4.65E-104	0.278673774	0.999	1	8.63E-100
		Srpx2	3.43E-225	1.131915714	0.621	0.165	6.36E-221
		Cthrc1	2.71E-93	1.02357134	0.79	0.587	5.02E-89
		Zfand5	1.46E-102	0.524949298	0.922	0.798	2.71E-98
		Hspg2	1.20E-61	0.507279814	0.879	0.817	2.23E-57
		Angptl1	7.36E-85	0.473047163	0.165	0.006	1.36E-80
		Tmem100	3.88E-50	0.372048471	0.257	0.087	7.20E-46

	Morphogenesis	Mfap2	2.30E-65	0.626982705	0.633	0.412	4.27E-61
		Mfap5	3.08E-258	1.989603064	0.493	0.038	5.72E-254
		Ctnnbip1	9.77E-16	0.268767132	0.658	0.627	1.81E-11
		Ttn	1.53E-49	0.290716902	0.123	0.013	2.85E-45
		Psmb10	1.57E-18	0.277849321	0.782	0.745	2.92E-14
		Cdh11	5.40E-61	0.452364766	0.415	0.192	1.00E-56
		Mdfi	8.59E-88	0.355751101	0.266	0.048	1.59E-83
		Ctsh	2.62E-57	0.444936782	0.366	0.159	4.86E-53
		Meox1	4.96E-25	0.270038672	0.128	0.041	9.20E-21
		Col12a1	5.47E-156	0.890871172	0.394	0.062	1.01E-151
		Kdm6b	9.29E-10	0.264473206	0.562	0.516	1.72E-05
		Rhoj	3.59E-69	0.505974515	0.546	0.318	6.66E-65
		Nbl1	7.25E-158	0.886783996	0.858	0.602	1.34E-153
		Jam3	2.35E-47	0.350956393	0.589	0.41	4.37E-43
		Jun	2.92E-19	0.400886963	0.991	0.993	5.41E-15
		Plpp3	5.06E-38	0.491601035	0.778	0.687	9.38E-34
		Gas1	5.05E-103	0.956141291	0.517	0.237	9.36E-99
		Angptl4	1.41E-86	0.782464546	0.885	0.748	2.62E-82
		Rdh10	4.04E-23	0.395224866	0.628	0.55	7.49E-19
		Mycbp2	2.16E-55	0.389255079	0.606	0.427	4.00E-51
		Ndn	4.33E-75	0.429693327	0.348	0.112	8.03E-71
		Pkd2	4.34E-47	0.400110255	0.439	0.258	8.05E-43
		Ace	3.43E-152	1.051130938	0.435	0.094	6.36E-148
		Fam20a	1.10E-42	0.306811897	0.316	0.144	2.03E-38
		Prrx2	7.30E-61	0.490291802	0.444	0.237	1.35E-56
		Itga7	5.18E-37	0.350215876	0.236	0.098	9.61E-33
		Ncam1	2.16E-23	0.297613216	0.611	0.511	4.01E-19
		Fap	2.68E-64	0.502866213	0.396	0.18	4.96E-60
		Amotl2	1.10E-17	0.289496916	0.439	0.338	2.04E-13
		Dlc1	1.27E-52	0.301319749	0.296	0.111	2.35E-48
		Nr4a2	2.03E-33	0.518948664	0.538	0.404	3.77E-29
		Mgp	4.46E-246	2.192291309	0.617	0.126	8.27E-242
		Lama2	4.28E-70	0.25607161	0.215	0.038	7.94E-66
		Nfix	1.46E-43	0.318741422	0.423	0.241	2.70E-39
		Unc93b1	2.02E-51	0.33465038	0.432	0.228	3.74E-47
		Slc39a1	7.89E-34	0.337772805	0.737	0.594	1.46E-29
		Aqp1	3.34E-15	0.642813594	0.187	0.105	6.19E-11
		Gfra1	1.88E-40	0.315753778	0.22	0.078	3.48E-36
		Bmper	2.72E-126	0.446756949	0.252	0.013	5.04E-122
		Actn3	1.62E-52	0.472102053	0.152	0.024	3.01E-48
		Thra	5.61E-47	0.363429709	0.484	0.298	1.04E-42
		Antxr1	4.04E-142	0.645882275	0.52	0.171	7.49E-138
		Tnnt3	1.42E-62	0.570811599	0.144	0.012	2.63E-58
		Tnnt2	4.13E-15	0.322913142	0.113	0.048	7.67E-11
		Slc39a14	1.36E-42	0.360683541	0.474	0.301	2.52E-38
		Emb	3.64E-94	0.828763185	0.893	0.676	6.75E-90
		Svep1	7.25E-92	0.342806625	0.18	0.007	1.34E-87
		Vcam1	9.59E-130	1.136517896	0.565	0.238	1.78E-125
		Thbs4	1.85E-139	1.834292723	0.503	0.166	3.44E-135
		Ugdh	2.02E-52	0.388636523	0.936	0.862	3.75E-48
		Rps6	0	0.545426482	1	1	0
		Pcsk5	4.08E-53	0.302622489	0.198	0.046	7.57E-49
		Thbs3	2.22E-59	0.349754282	0.34	0.135	4.11E-55
	VEGF-activated platelet-derived growth factor receptor signaling pathway	Pdgfrb	6.50E-87	0.61478241	0.41	0.148	1.21E-82
		Pdgfra	4.35E-40	0.571378594	0.597	0.492	8.06E-36
		Vegfa	1.20E-10	0.264881109	0.407	0.342	2.22E-06
	Negative regulation of fibroblast growth factor production	Fgfr4	3.93E-83	0.513870488	0.16	0.005	7.30E-79
		Fgfr1	9.66E-61	0.369474925	0.95	0.903	1.79E-56
		Pgm2	5.15E-56	0.38023037	0.535	0.322	9.55E-52
		H2afy	2.96E-64	-0.301005218	0.975	0.993	5.50E-60
		H2afx	3.51E-40	-0.400066294	0.619	0.76	6.50E-36
		Cygb	1.93E-155	0.979478639	0.41	0.069	3.58E-151
		Polr2l	1.03E-31	0.274276371	0.705	0.64	1.92E-27
		Gpi1	6.99E-49	0.262080395	0.999	0.994	1.30E-44
		St3gal1	9.50E-39	0.290978757	0.652	0.502	1.76E-34
		Ddit4l	6.44E-35	0.285217287	0.168	0.051	1.19E-30
		Dpm3	3.16E-62	0.354436207	0.947	0.886	5.86E-58
		Dnajb9	1.40E-19	0.254075878	0.806	0.728	2.59E-15
		Oxct1	2.23E-21	0.262067959	0.661	0.604	4.13E-17
		Rpl36al	1.26E-84	0.256549271	1	0.999	2.33E-80
		P4ha3	8.87E-158	0.62483664	0.366	0.044	1.64E-153
		Gabarap	1.87E-68	0.310739365	1	1	3.48E-64
		Jund	2.22E-201	0.936169287	0.93	0.733	4.11E-197
		Fah	5.61E-45	0.340066746	0.433	0.251	1.04E-40
		Camk4	7.53E-51	0.276736595	0.194	0.047	1.40E-46
		Psmb9	7.41E-64	0.30497442	0.222	0.048	1.37E-59
		Cpq	2.90E-41	0.378537077	0.522	0.365	5.37E-37
		Uap1	7.20E-21	0.384785478	0.844	0.793	1.34E-16
		Dram1	4.93E-121	0.571505866	0.423	0.117	9.14E-117
		Rpl27a	2.52E-279	0.482245044	1	1	4.67E-275
		Eef1a1	2.83E-140	0.339146192	1	1	5.24E-136
		Ost4	3.08E-126	0.447003339	0.987	0.972	5.71E-122
		Pla1a	1.10E-121	0.868284197	0.385	0.089	2.03E-117
		Mrpl33	9.83E-64	0.321510472	0.969	0.882	1.82E-59
		Maf	4.47E-63	0.357500328	0.31	0.107	8.29E-59
		Rpl39	2.49E-139	0.280231005	1	1	4.61E-135

		Metabolic process	Iffo1	1.10E-22	0.312940309	0.233	0.124	2.04E-18
			Ndufa5	2.75E-20	0.254932151	0.974	0.958	5.10E-16
			Dpep1	1.07E-94	0.610678848	0.299	0.063	1.99E-90
			Rarres2	1.58E-120	1.221934224	0.226	0.007	2.94E-116
			Sh3glb1	2.63E-96	0.656549821	0.925	0.823	4.87E-92
			Hacd2	2.39E-31	0.43120632	0.728	0.65	4.43E-27
			Asb5	2.02E-100	0.854796997	0.227	0.02	3.75E-96
			Selm	9.44E-200	1.16597767	0.868	0.617	1.75E-195
			Nsg1	4.33E-52	0.56930831	0.557	0.366	8.03E-48
			Gzmd	6.73E-65	0.823629394	0.134	0.007	1.25E-60
			Rbp1	1.57E-106	0.700970373	0.897	0.75	2.91E-102
			Mgst1	1.83E-278	1.365402231	0.523	0.039	3.40E-274
			Slc7a2	1.74E-125	0.426815149	0.276	0.023	3.23E-121
			Rnaset2a	3.28E-34	0.366908003	0.522	0.392	6.08E-30
			Pappa	2.26E-25	0.267372311	0.17	0.068	4.19E-21
			Nudt4	4.90E-48	0.414426318	0.907	0.84	9.08E-44
			Rpl13	2.78E-143	0.285362258	1	1	5.16E-139
			Btg1	3.22E-48	0.362995379	0.887	0.791	5.98E-44
			Spock3	1.67E-13	0.376415355	0.299	0.217	3.10E-09
			Sepp1	4.39E-109	0.736321879	0.591	0.287	8.14E-105
			Bgn	8.46E-222	1.139106801	0.715	0.28	1.57E-217
			Plpp1	1.04E-18	0.26666278	0.687	0.615	1.94E-14
			Ctsc	1.92E-87	0.785748897	0.527	0.265	3.55E-83
			Ass1	6.15E-67	0.841808243	0.517	0.289	1.14E-62
			Map1lc3a	4.20E-49	0.303497244	0.983	0.954	7.80E-45
			Pim1	7.07E-46	0.482279053	0.724	0.569	1.31E-41
			Sdf2	9.76E-43	0.279311904	0.947	0.906	1.81E-38
			Moxd1	5.08E-82	0.375684812	0.16	0.006	9.42E-78
			Fosb	2.45E-51	0.648189636	0.607	0.444	4.54E-47
			Bag3	1.43E-19	0.510624379	0.881	0.854	2.66E-15
			Comt	2.08E-45	0.294060084	0.933	0.854	3.85E-41
			Gzme	5.67E-162	1.522808602	0.336	0.025	1.05E-157
			Luc7l2	2.52E-32	0.275498013	0.649	0.556	4.68E-28
			Ndufv3	3.73E-74	0.357992659	0.979	0.93	6.91E-70
			Prdx6	6.32E-34	0.254793292	0.997	0.998	1.17E-29
			Rpl7	1.30E-129	0.258565092	1	1	2.42E-125
			Svbp	6.62E-53	0.39809093	0.925	0.865	1.23E-48
			Abcg2	1.03E-34	0.256179072	0.338	0.181	1.91E-30
			Rtn2	2.06E-63	0.589584349	0.312	0.111	3.82E-59
			B3gnt9	8.74E-25	0.258482333	0.371	0.242	1.62E-20
			Hsd11b1	8.70E-139	0.722433113	0.317	0.032	1.61E-134
			Aebp1	2.85E-70	0.604617763	0.975	0.983	5.29E-66
			Arl1	1.67E-116	0.423273593	0.988	0.943	3.10E-112
			Tapbp	1.00E-99	0.679280895	0.681	0.446	1.86E-95
			Rpl22l1	3.31E-26	0.261417437	0.999	0.998	6.14E-22
			P4ha2	6.98E-14	0.258388105	0.627	0.579	1.29E-09
			Bnip3l	6.76E-53	0.288148491	0.936	0.845	1.25E-48
			Serinc3	6.38E-69	0.39254241	0.891	0.775	1.18E-64
			Oat	6.42E-73	0.445947509	0.95	0.879	1.19E-68
			Fitm1	7.22E-71	0.375729892	0.145	0.007	1.34E-66
			Aldh3b1	2.00E-23	0.255484225	0.22	0.109	3.71E-19
			Serinc2	1.44E-35	0.347979633	0.39	0.231	2.67E-31
			Gzmc	3.81E-56	0.730687863	0.114	0.005	7.06E-52
			Tmem165	2.91E-68	0.402816983	0.667	0.497	5.39E-64
			Hnrnpa1	2.99E-39	0.290768555	0.927	0.861	5.55E-35
			Atp5e	7.23E-110	0.28209092	1	1	1.34E-105
			Rnf187	4.62E-107	0.460684077	0.841	0.66	8.56E-103
			Rpl15	0	0.757749292	1	1	0
			Commd1	6.10E-30	0.255401498	0.507	0.377	1.13E-25
			Acaa1a	1.65E-44	0.271838743	0.922	0.848	3.06E-40
			Dtx3	1.29E-28	0.284501421	0.505	0.372	2.39E-24
			Dapk2	3.33E-54	0.367444498	0.164	0.027	6.18E-50
			S100a9	1.72E-199	1.40070213	0.603	0.17	3.19E-195
			Pitpnc1	5.94E-19	0.260539157	0.313	0.205	1.10E-14
			Sod3	9.74E-251	1.825116122	0.499	0.048	1.81E-246
			Bmp1	1.12E-96	0.673397541	0.73	0.525	2.08E-92
			Taf10	5.36E-100	0.429849005	0.947	0.843	9.95E-96
			Adam12	2.25E-121	0.602656579	0.352	0.069	4.17E-117
			Plod2	2.25E-39	0.354402431	0.642	0.506	4.17E-35
			Hspa8	9.37E-58	0.288336711	1	1	1.74E-53
			Plod1	7.46E-38	0.37974959	0.665	0.549	1.38E-33
			Nfia	2.40E-160	0.635181552	0.461	0.099	4.44E-156
			Itm2a	2.79E-67	0.990449999	0.858	0.712	5.17E-63
			Rnase4	1.05E-157	1.29669011	0.78	0.51	1.94E-153
			Ak1	3.00E-24	0.312689273	0.55	0.442	5.56E-20
			Arpc3	1.26E-54	0.287603507	0.973	0.926	2.33E-50
			Gpx3	7.10E-143	1.386922578	0.507	0.168	1.32E-138
			Erlec1	1.66E-37	0.281484232	0.889	0.84	3.08E-33
			Cpxm1	1.27E-101	0.956303629	0.446	0.164	2.35E-97
			Celf2	1.12E-34	0.351225408	0.544	0.403	2.08E-30
			Gstm2	2.54E-45	0.389984411	0.435	0.257	4.71E-41
			Hp	1.25E-46	0.3727678	0.119	0.014	2.32E-42
			Mcpt8	9.09E-74	1.171608098	0.463	0.221	1.69E-69
			Ldhb	1.28E-06	0.303383922	0.429	0.412	0.023766191
			Ctla2a	2.52E-97	1.044142062	0.215	0.017	4.67E-93
			Psmb8	8.99E-171	0.936140293	0.553	0.153	1.67E-166

		Bcat1	8.09E-27	0.306903606	0.591	0.455	1.50E-22
		C1ra	6.22E-149	0.489605985	0.277	0.009	1.15E-144
		Kng2	4.38E-114	0.596854844	0.268	0.028	8.12E-110
		Lmcd1	5.22E-117	0.686793035	0.24	0.014	9.68E-113
		Camp	8.44E-51	0.606343386	0.161	0.029	1.57E-46
		Spon1	7.32E-184	1.325688572	0.375	0.03	1.36E-179
		Lrpap1	1.26E-32	0.30697192	0.672	0.577	2.34E-28
		Mxd4	2.37E-35	0.331402895	0.6	0.47	4.40E-31
		Tsc22d1	1.39E-30	0.36899925	0.972	0.944	2.59E-26
		Ndufa3	5.36E-77	0.323410957	0.981	0.952	9.94E-73
		Zbtb20	2.66E-73	0.591674786	0.754	0.585	4.93E-69
		Tnni2	2.27E-78	0.987302862	0.182	0.017	4.21E-74
		Ndufa4l2	9.69E-35	0.918013499	0.275	0.128	1.80E-30
		Dnajb1	8.95E-27	0.624154078	0.977	0.961	1.66E-22
		Psme2	1.47E-58	0.398834598	0.959	0.916	2.73E-54
		Igfbp4	7.10E-27	0.637111446	0.947	0.893	1.32E-22
		Vgll3	2.95E-139	0.440067666	0.295	0.023	5.46E-135
		Peg3	2.02E-121	0.670528181	0.227	0.007	3.76E-117
		Maged1	3.20E-63	0.346569812	0.979	0.955	5.94E-59
		Fndc1	1.99E-69	0.444479884	0.21	0.037	3.70E-65
		Itm2b	1.82E-128	0.590788265	1	0.997	3.38E-124
		Crip1	5.37E-179	1.30443862	0.87	0.61	9.97E-175
		Slpi	3.81E-32	0.351227164	0.314	0.157	7.07E-28
		Psme1	7.82E-29	0.251971588	0.952	0.916	1.45E-24
		Cilp	8.24E-143	1.245007411	0.331	0.038	1.53E-138
		Il17rc	3.82E-28	0.253377292	0.476	0.344	7.08E-24
		Adam9	1.63E-22	0.251497428	0.602	0.522	3.03E-18
		Ech1	2.84E-32	0.250613478	0.93	0.875	5.28E-28
		Ostc	1.12E-51	0.290965305	0.993	0.985	2.07E-47
		Prss23	4.09E-14	0.252299004	0.295	0.201	7.58E-10
		Ndufa4	1.62E-32	0.297994303	0.983	0.975	3.01E-28
		Pde4b	1.24E-116	0.549329087	0.421	0.118	2.31E-112
		Atp1a1	2.21E-29	0.288056526	0.995	0.98	4.10E-25
		Kcnj15	1.31E-65	0.257669244	0.128	0.004	2.44E-61
		Scarb2	1.75E-28	0.278454133	0.589	0.47	3.24E-24
		Slc16a3	6.83E-32	0.355335518	0.481	0.301	1.27E-27
		Fxyd1	3.10E-142	0.548540443	0.3	0.023	5.74E-138
		Ap3s1	4.15E-42	0.282466362	0.826	0.726	7.69E-38
		Slc50a1	3.90E-34	0.262464231	0.878	0.814	7.23E-30
		Maged2	3.95E-34	0.332604629	0.871	0.8	7.33E-30
		Scn7a	2.63E-65	0.264791193	0.133	0.006	4.87E-61
		Slc29a1	3.35E-29	0.521850906	0.386	0.247	6.22E-25
	Ion transport	Fkbp7	6.99E-81	0.575062513	0.825	0.676	1.30E-76
		Jsrp1	6.44E-59	0.487885346	0.18	0.032	1.19E-54
		Fkbp9	3.68E-142	0.5733045	0.967	0.868	6.82E-138
		Snx7	6.49E-36	0.299900723	0.749	0.653	1.20E-31
		Slc44a2	2.84E-76	0.452959175	0.556	0.307	5.26E-72
		Copz2	4.83E-112	0.711669336	0.794	0.586	8.96E-108
		Saraf	3.12E-70	0.445734836	0.964	0.938	5.79E-66
		Fxyd6	1.21E-31	0.402989541	0.22	0.093	2.25E-27
		Slc6a6	5.85E-14	0.321390363	0.722	0.686	1.09E-09
		Lcn2	2.34E-28	0.386570416	0.144	0.045	4.34E-24
		Kcne4	4.69E-118	0.754087168	0.312	0.048	8.70E-114
		Pdia4	1.03E-31	0.263343779	0.963	0.909	1.91E-27
		Phlda3	9.22E-34	0.396785929	0.603	0.474	1.71E-29
		Gpx7	4.84E-65	0.502394458	0.842	0.731	8.97E-61
		Wisp2	3.16E-156	1.138174513	0.298	0.013	5.86E-152
		Slfn2	2.66E-33	0.294806136	0.178	0.059	4.94E-29
	Response to stimulus	Susd6	1.05E-44	0.350377209	0.47	0.29	1.94E-40
		Mest	9.36E-90	1.541023632	0.328	0.087	1.74E-85
		Colec12	4.00E-76	0.58701214	0.672	0.492	7.43E-72
		Nucb1	3.06E-36	0.271745682	0.895	0.847	5.67E-32
		Mt2	1.66E-07	0.264947278	0.999	0.996	0.003079074
		H2-T22	2.79E-51	0.384301474	0.489	0.292	5.17E-47
		Fcgrt	6.78E-168	0.896928132	0.662	0.286	1.26E-163
		Brinp3	7.69E-100	0.383022151	0.228	0.021	1.43E-95
		Retnlg	3.73E-68	0.519881886	0.206	0.035	6.92E-64
		Fibin	2.15E-214	1.481240928	0.417	0.029	3.99E-210
		Arhgef25	5.84E-36	0.30941453	0.545	0.401	1.08E-31
	Signal transduction	Acta2	8.31E-78	1.241816767	0.463	0.208	1.54E-73
		Tspan6	1.06E-133	0.712063511	0.699	0.402	1.96E-129
		Cnrip1	4.02E-27	0.255726653	0.477	0.336	7.45E-23
		Glipr2	1.02E-46	0.349107931	0.854	0.712	1.89E-42
		Malat1	2.26E-56	0.477577244	0.999	1	4.20E-52
		Fas	1.41E-46	0.400273578	0.302	0.126	2.62E-42
		Fstl1	3.88E-142	1.44403949	0.899	0.752	7.19E-138
	Cell death	Dusp1	5.51E-13	0.33465906	0.864	0.767	1.02E-08
		Casp12	2.11E-37	0.310594671	0.462	0.289	3.92E-33
		Srpx	2.28E-157	0.593194511	0.345	0.034	4.22E-153
	Golgi protein	Tgoln1	7.29E-41	0.274218267	0.814	0.716	1.35E-36
	Protein folding	Fkbp14	6.94E-28	0.287751667	0.447	0.31	1.29E-23
	Hepran synthesis	Hs3st1	1.10E-52	0.357268591	0.227	0.063	2.04E-48
		H19	3.31E-168	2.001354894	0.383	0.046	6.13E-164
		C1qtnf6	2.07E-97	0.980633331	0.72	0.513	3.84E-93
		Pdgfrl	7.24E-186	0.912296097	0.448	0.067	1.34E-181
		Ms4a4d	1.17E-209	0.861609625	0.372	0.011	2.18E-205

		500015O10Ri	3.59E-108	0.836201591	0.247	0.023	6.66E-104
		Lhfp	3.34E-122	0.833689765	0.824	0.644	6.19E-118
		Pnrc1	1.70E-152	0.805006232	0.898	0.692	3.15E-148
		Pla2g16	2.68E-159	0.79909937	0.515	0.152	4.96E-155
		Plxdc2	1.12E-218	0.798634119	0.475	0.056	2.08E-214
		C1s1	1.55E-177	0.741089725	0.334	0.014	2.87E-173
		Serf2	9.19E-239	0.634133817	0.997	0.958	1.70E-234
		Meg3	1.19E-99	0.617257381	0.385	0.114	2.21E-95
		Ftl1	5.92E-110	0.54435016	1	1	1.10E-105
		Snhg18	1.19E-65	0.531196733	0.878	0.802	2.21E-61
		Fam46a	1.53E-39	0.475286362	0.577	0.424	2.84E-35
		Gm3511	1.72E-204	0.448803382	1	1	3.20E-200
		030408B16Ri	2.94E-48	0.366469014	0.154	0.029	5.45E-44
		Gm10736	2.05E-125	0.356803527	0.999	0.999	3.80E-121
		Akr1b3	1.84E-24	0.343124443	0.545	0.47	3.42E-20
		Sdpr	7.18E-44	0.330998245	0.264	0.096	1.33E-39
		Rps27rt	2.23E-53	0.326699269	0.78	0.665	4.14E-49
		Gm8818	7.68E-13	0.325148268	0.236	0.155	1.42E-08
		Dleu2	1.10E-24	0.291132263	0.385	0.26	2.04E-20
		Rian	1.25E-62	0.287320682	0.241	0.06	2.32E-58
	Unclear functions	Tmem140	9.06E-57	0.268527776	0.216	0.052	1.68E-52
		Laptm4a	1.66E-54	0.315329184	1	1	3.08E-50
		Ptms	3.12E-54	0.293997908	0.969	0.916	5.78E-50
		Lrrc15	8.13E-49	0.261647015	0.161	0.031	1.51E-44
		Phf11d	2.05E-46	0.251052448	0.134	0.02	3.80E-42
		Nt5dc2	2.85E-46	0.403964045	0.906	0.779	5.29E-42
		Scpep1	1.02E-39	0.276616127	0.752	0.624	1.89E-35
		Smim3	4.33E-36	0.320660032	0.874	0.788	8.03E-32
		Fam114a1	8.48E-36	0.345321373	0.581	0.448	1.57E-31
		Rabac1	2.84E-30	0.270007576	0.939	0.934	5.27E-26
		Ginm1	3.26E-27	0.303728342	0.589	0.493	6.04E-23
		Yrdc	2.25E-21	0.272280862	0.528	0.456	4.17E-17
		Fkbp11	7.56E-21	0.256519364	0.913	0.861	1.40E-16
		Tmem86a	5.20E-16	0.261289808	0.357	0.257	9.65E-12
		Filip1l	1.90E-14	0.342982114	0.529	0.46	3.52E-10
		Chic2	2.85E-83	0.392472854	0.925	0.8	5.29E-79
		Hcfc1r1	4.50E-64	0.371930567	0.967	0.924	8.35E-60
		Dhrs7	1.20E-60	0.414569868	0.471	0.251	2.23E-56
		Gm3699	1.36E-74	0.276663238	0.999	0.996	2.52E-70
		Gm10076	1.48E-71	0.267332966	0.999	0.994	2.75E-67
		Gltscr2	4.79E-45	0.263192931	0.995	0.982	8.88E-41
		110065P20Ri	3.30E-43	0.262955874	0.925	0.871	6.12E-39
		Spats2l	3.00E-37	0.37594407	0.351	0.192	5.56E-33
		AW112010	1.05E-20	0.25943544	0.182	0.086	1.95E-16
		Rn7sk	8.65E-10	0.259413486	0.379	0.307	1.60E-05
		Fam198b	2.32E-10	0.256634473	0.326	0.254	4.30E-06
Downregulated genes		Tes	7.51E-61	-0.352857856	0.766	0.884	1.39E-56
		Ccnd1	7.24E-189	-0.819739803	0.951	0.99	1.34E-184
		Tipin	8.03E-44	-0.30977594	0.869	0.91	1.49E-39
		Ercc1	1.82E-74	-0.439014415	0.955	0.974	3.38E-70
		Nasp	3.81E-54	-0.31476575	0.89	0.936	7.07E-50
		Mcm7	1.01E-39	-0.269570772	0.491	0.697	1.88E-35
		Aaas	7.07E-45	-0.258709686	0.414	0.658	1.31E-40
		Ezr	5.29E-51	-0.369538385	0.362	0.611	9.81E-47
		Chaf1a	2.08E-47	-0.284807917	0.29	0.523	3.85E-43
		Nup37	1.02E-35	-0.282267635	0.496	0.678	1.89E-31
		Rala	9.11E-126	-0.427265303	0.995	0.996	1.69E-121
		Hells	5.39E-47	-0.288884604	0.389	0.644	9.99E-43
		Cenpa	2.81E-48	-0.458637497	0.764	0.829	5.22E-44
		Rae1	1.55E-78	-0.331088064	0.922	0.961	2.88E-74
		Eps8	1.41E-190	-0.67038879	0.93	0.987	2.62E-186
		Cdca8	2.12E-38	-0.358970015	0.565	0.698	3.92E-34
		Sdccag3	6.88E-44	-0.259388761	0.726	0.854	1.28E-39
		Cep164	2.80E-54	-0.286008611	0.431	0.693	5.20E-50
		Setd8	3.93E-46	-0.288053221	0.706	0.843	7.28E-42
		Nsmce2	9.60E-42	-0.344659569	0.912	0.952	1.78E-37
		Sgsm3	1.44E-38	-0.251381193	0.3	0.506	2.67E-34
		Cdkn2b	7.19E-36	-0.300412528	0.933	0.948	1.33E-31
		Avpi1	3.39E-56	-0.40237483	0.788	0.882	6.29E-52
		Kif20a	4.21E-42	-0.26449274	0.235	0.462	7.81E-38
		Gmnn	1.55E-55	-0.386547416	0.482	0.704	2.87E-51
		Cdc20	3.73E-54	-0.438281992	0.694	0.797	6.91E-50
		Mapk12	3.30E-78	-0.34735204	0.275	0.588	6.12E-74
		Tpx2	3.26E-56	-0.401771271	0.354	0.614	6.04E-52
		Ndc1	1.37E-56	-0.321592497	0.418	0.676	2.54E-52
		Dbf4	1.88E-33	-0.252936708	0.439	0.644	3.49E-29
		Smc1a	6.43E-42	-0.253972854	0.904	0.933	1.19E-37
		Cdc123	1.56E-45	-0.254668374	0.896	0.934	2.89E-41
		Nsun2	5.01E-52	-0.259479407	0.96	0.963	9.29E-48
		Plk1	4.19E-38	-0.260698101	0.212	0.421	7.77E-34
		Spc25	1.41E-35	-0.261990114	0.299	0.52	2.62E-31
		Bub3	1.03E-42	-0.262938194	0.949	0.95	1.91E-38
		Cenph	2.26E-45	-0.264864389	0.286	0.533	4.19E-41
		Kif2a	1.05E-41	-0.268244925	0.488	0.707	1.95E-37
		Ier3	2.23E-18	-0.268342202	0.969	0.974	4.14E-14
		Cdca3	4.16E-30	-0.272182341	0.557	0.696	7.71E-26

	Cell Cycle	Tacc3	1.68E-45	-0.272343338	0.218	0.446	3.11E-41
		Racgap1	4.80E-34	-0.273229872	0.471	0.657	8.89E-30
		Ccnb2	2.34E-29	-0.274466141	0.539	0.682	4.34E-25
		Mcm3	8.53E-36	-0.276518663	0.421	0.645	1.58E-31
		Smc2	4.79E-35	-0.278750403	0.693	0.8	8.89E-31
		Ran	1.63E-68	-0.278986616	1	0.998	3.02E-64
		Tsg101	1.03E-72	-0.279345485	0.975	0.986	1.90E-68
		Rrs1	3.92E-35	-0.283404563	0.538	0.709	7.27E-31
		Fgf10	2.25E-63	-0.283794426	0.13	0.386	4.17E-59
		Triobp	1.01E-46	-0.288496015	0.528	0.738	1.87E-42
		E2f8	8.04E-56	-0.28864119	0.177	0.421	1.49E-51
		Psma8	2.75E-79	-0.292093258	0.125	0.41	5.10E-75
		Ppp1ca	6.95E-119	-0.292404315	1	1	1.29E-114
		Cenpf	4.22E-39	-0.293344464	0.228	0.444	7.82E-35
		Nup43	4.58E-52	-0.293375717	0.371	0.631	8.50E-48
		Bcl2l1	1.13E-57	-0.293571405	0.34	0.617	2.10E-53
		Zw10	2.12E-51	-0.30183121	0.256	0.49	3.93E-47
		Arpp19	3.44E-99	-0.302282503	1	0.999	6.38E-95
		Rassf1	2.50E-40	-0.303046407	0.91	0.935	4.64E-36
		Tuba1b	1.53E-54	-0.303852326	0.993	0.999	2.83E-50
		Mad1l1	4.51E-56	-0.307272568	0.318	0.579	8.36E-52
		Rps27l	2.04E-98	-0.308009456	1	1	3.79E-94
		Cfl1	2.57E-123	-0.310067108	1	1	4.77E-119
		Prc1	1.65E-40	-0.312764444	0.314	0.54	3.07E-36
		Cks1b	2.02E-64	-0.323534265	0.983	0.991	3.74E-60
		Pbk	7.09E-44	-0.32485842	0.293	0.541	1.32E-39
		Tuba1a	6.04E-41	-0.327445173	0.994	0.998	1.12E-36
		Cdkn1a	5.32E-23	-0.330647579	0.665	0.762	9.87E-19
		Birc5	1.09E-28	-0.331540119	0.667	0.742	2.02E-24
		Tpr	5.55E-104	-0.343848236	0.994	0.998	1.03E-99
		Tpd52l1	1.88E-73	-0.352116417	0.261	0.567	3.49E-69
		Tubb5	7.23E-69	-0.35532447	0.998	0.999	1.34E-64
		Cdk4	3.06E-123	-0.357387296	1	1	5.68E-119
		Mcm6	5.86E-49	-0.360479393	0.625	0.785	1.09E-44
		Rbm38	4.04E-70	-0.36335254	0.435	0.722	7.50E-66
		Ranbp1	5.58E-110	-0.385897532	0.999	1	1.03E-105
		Trip13	2.94E-66	-0.395568157	0.313	0.595	5.46E-62
		Zwint	2.85E-89	-0.404524794	0.93	0.964	5.28E-85
		Eif4ebp1	1.70E-141	-0.414682775	1	1	3.15E-137
		Cdk1	3.88E-33	-0.416682229	0.706	0.769	7.19E-29
		Calm1	2.50E-166	-0.421667877	1	1	4.63E-162
		Ccnb1	1.30E-50	-0.429132326	0.385	0.608	2.41E-46
		Pin1	1.95E-151	-0.432720984	0.993	0.996	3.61E-147
		Tuba1c	8.03E-84	-0.498145405	0.933	0.964	1.49E-79
		Tuba4a	1.32E-99	-0.550043179	0.589	0.822	2.45E-95
		Cdkn2a	3.15E-154	-0.550505768	0.994	1	5.85E-150
		Tubb4b	6.83E-109	-0.552960233	0.951	0.975	1.27E-104
		Tubb6	9.26E-189	-0.838230644	0.993	0.997	1.72E-184
		Aurka	4.92E-64	-0.44237244	0.291	0.573	9.12E-60
		Btc	3.86E-104	-0.719605877	0.151	0.497	7.17E-100
		Atp2b1	6.13E-40	-0.252344843	0.981	0.989	1.14E-35
		Wwc1	5.06E-53	-0.252783841	0.128	0.352	9.38E-49
		Wars	3.03E-46	-0.25354565	0.349	0.592	5.61E-42
		Rsl1d1	2.81E-52	-0.25466097	0.981	0.991	5.21E-48
		Myl12b	1.59E-39	-0.25662531	0.953	0.968	2.96E-35
		Mllt3	1.14E-36	-0.257539905	0.314	0.522	2.12E-32
		Hmces	4.04E-48	-0.270419731	0.507	0.741	7.49E-44
		Rnh1	2.98E-70	-0.272961715	0.999	1	5.53E-66
		Nefm	3.26E-56	-0.275047989	0.121	0.349	6.04E-52
		Tmbim1	8.87E-46	-0.277721943	0.744	0.876	1.64E-41
		Ociad2	1.60E-74	-0.519737562	0.732	0.854	2.97E-70
		Cyth2	2.58E-63	-0.296920818	0.942	0.977	4.79E-59
		Hn1	3.18E-120	-0.414906292	0.987	0.997	5.90E-116
		Syne3	7.73E-64	-0.298962347	0.135	0.385	1.43E-59
		Epha2	2.22E-47	-0.29935885	0.35	0.605	4.12E-43
		Parvb	2.28E-62	-0.333219593	0.461	0.726	4.22E-58
		Ybx3	2.02E-84	-0.338234524	0.964	0.987	3.75E-80
		Anxa7	4.47E-72	-0.338311628	0.974	0.987	8.29E-68
		Sh3kbp1	3.10E-94	-0.368781148	0.914	0.973	5.74E-90
		Msn	8.88E-86	-0.381470394	0.986	0.993	1.65E-81
		Pxn	5.97E-98	-0.430250141	0.809	0.92	1.11E-93
		Pde3b	2.17E-98	-0.453914766	0.352	0.697	4.03E-94
		Eef1e1	1.73E-140	-0.502257523	0.948	0.985	3.21E-136
		Chn1	4.50E-124	-0.560684821	0.379	0.736	8.34E-120
		Phlda2	9.81E-81	-0.579461561	0.132	0.425	1.82E-76
		Dusp9	8.97E-119	-0.619975588	0.381	0.722	1.66E-114
		Cpe	1.45E-195	-0.62885794	0.931	0.999	2.70E-191
		Ghr	5.44E-151	-0.647014605	0.775	0.927	1.01E-146
		Gal	2.83E-162	-1.79956159	0.674	0.872	5.24E-158
		Klhl1	1.44E-52	-0.32142266	0.066	0.261	2.67E-48
		Vasp	1.50E-47	-0.250161976	0.989	0.996	2.78E-43
		Dazap1	5.36E-58	-0.254720596	0.988	0.995	9.94E-54
		Cdh13	1.31E-35	-0.258822677	0.239	0.444	2.43E-31
		Tmem120a	2.66E-39	-0.259627897	0.83	0.892	4.94E-35
		Cacybp	6.66E-53	-0.260499633	0.993	0.996	1.24E-48
		Tbcb	5.74E-72	-0.263930986	0.989	0.995	1.06E-67

	Developmental process	Gas8	5.27E-45	-0.264949064	0.407	0.649	9.77E-41
		Smarcc1	9.97E-47	-0.269384258	0.909	0.952	1.85E-42
		Marcksl1	2.01E-63	-0.270636044	0.985	0.995	3.73E-59
		Cep57	2.39E-46	-0.280398627	0.437	0.68	4.42E-42
		Prrc2c	8.49E-59	-0.283595392	0.979	0.992	1.57E-54
		Lhx6	2.28E-66	-0.285358266	0.144	0.411	4.24E-62
		Bzw2	5.53E-50	-0.286167569	0.906	0.95	1.03E-45
		Sh2b3	4.40E-45	-0.286352528	0.254	0.485	8.16E-41
		Pdlim2	9.56E-23	-0.299557464	0.876	0.895	1.77E-18
		Pitpna	6.62E-78	-0.299824159	0.985	0.99	1.23E-73
		Pir	7.99E-55	-0.303624431	0.267	0.521	1.48E-50
		Lhx8	8.85E-48	-0.305382293	0.191	0.415	1.64E-43
		Lefty1	6.22E-53	-0.311726178	0.34	0.593	1.15E-48
		Fam212a	1.23E-43	-0.31285194	0.239	0.463	2.29E-39
		Tshz1	9.40E-44	-0.312889521	0.476	0.694	1.74E-39
		Pcdh8	1.07E-59	-0.316739526	0.096	0.328	1.99E-55
		Pfn1	1.50E-110	-0.318754924	1	1	2.78E-106
		Tnfaip2	1.33E-49	-0.325774601	0.65	0.809	2.46E-45
		Lgals1	7.15E-110	-0.33002975	1	1	1.33E-105
		Capg	7.55E-87	-0.330347518	1	1	1.40E-82
		C1qb	5.17E-34	-0.331166049	0.034	0.16	9.59E-30
		Actb	1.30E-64	-0.331996819	1	1	2.41E-60
		Tspan8	5.27E-45	-0.337244706	0.033	0.185	9.77E-41
		Fhl2	2.14E-72	-0.349400471	0.948	0.977	3.98E-68
		Pfdn1	1.83E-117	-0.357611408	0.999	1	3.40E-113
		C1qa	7.84E-33	-0.358387824	0.028	0.146	1.45E-28
		Fosl1	1.07E-72	-0.361676385	0.885	0.952	1.98E-68
		Nr2f2	3.73E-55	-0.362507294	0.437	0.683	6.91E-51
		Tpd52	5.56E-91	-0.371777147	0.797	0.933	1.03E-86
		Pak1ip1	2.62E-101	-0.383479677	0.966	0.986	4.85E-97
		Crmp1	9.52E-95	-0.401735266	0.217	0.579	1.77E-90
		Tmem108	6.94E-86	-0.402941906	0.14	0.448	1.29E-81
		Atxn10	9.39E-106	-0.405423208	0.999	0.998	1.74E-101
		Phactr1	1.87E-48	-0.410884889	0.186	0.404	3.46E-44
		Cnr1	1.31E-100	-0.436207183	0.22	0.591	2.42E-96
		Stmn2	1.24E-30	-0.448936443	0.136	0.303	2.30E-26
		Nrn1	4.68E-24	-0.467419235	0.485	0.595	8.68E-20
		Chrna1	8.27E-110	-0.482392161	0.859	0.968	1.53E-105
		Hbegf	1.35E-59	-0.503719251	0.909	0.928	2.51E-55
		Stc1	2.52E-50	-0.510031334	0.565	0.738	4.67E-46
		Plet1	8.44E-64	-0.516513097	0.102	0.346	1.57E-59
		Ncl	3.47E-148	-0.536048146	1	1	6.43E-144
		Itga6	1.90E-142	-0.605719893	0.403	0.784	3.53E-138
		Cryab	9.52E-24	-0.619414416	0.783	0.796	1.77E-19
		Dkk2	7.28E-121	-0.627262785	0.858	0.959	1.35E-116
		Gpr149	1.01E-178	-0.703170758	0.151	0.638	1.87E-174
		Alcam	2.31E-188	-0.724785898	0.82	0.957	4.29E-184
		Itgb7	3.47E-134	-0.726931948	0.283	0.68	6.43E-130
		Cdx4	3.43E-83	-0.735785716	0.075	0.347	6.37E-79
		Nptx1	1.58E-154	-0.75545629	0.188	0.633	2.93E-150
		Tm4sf1	3.19E-202	-0.759046615	0.995	1	5.92E-198
		Gap43	3.03E-109	-0.821336596	0.705	0.829	5.61E-105
		Krt76	4.20E-129	-0.95265395	0.081	0.452	7.79E-125
		Esm1	4.50E-89	-0.998757788	0.574	0.733	8.35E-85
		Cst6	1.66E-145	-1.094832118	0.811	0.9	3.07E-141
		Tinag	4.69E-140	-0.965207733	0.071	0.462	8.70E-136
		Krt8	1.79E-99	-1.679895561	0.522	0.694	3.31E-95
		S100a6	4.27E-161	-0.451932088	1	1	7.92E-157
		Wdr1	8.00E-41	-0.256969314	0.984	0.985	1.48E-36
		Sox11	5.68E-44	-0.257194444	0.352	0.615	1.05E-39
		Tenm4	2.93E-56	-0.25985314	0.101	0.322	5.43E-52
		Arpc2	5.22E-90	-0.259999814	1	1	9.68E-86
		Dap	2.09E-64	-0.263067296	1	1	3.87E-60
		Klf5	2.07E-32	-0.269636656	0.611	0.766	3.84E-28
		Dnmt1	1.97E-41	-0.272533327	0.397	0.626	3.66E-37
		Hmgb3	4.97E-52	-0.27329771	0.399	0.67	9.22E-48
		Med28	3.82E-72	-0.275793389	0.993	0.992	7.09E-68
		C1qbp	1.76E-86	-0.285704914	0.999	1	3.27E-82
		Ascl1	3.02E-46	-0.286488737	0.029	0.184	5.61E-42
		Srrt	1.86E-51	-0.290843969	0.766	0.871	3.45E-47
		Lsm1	8.89E-68	-0.293108242	0.973	0.982	1.65E-63
		Bloc1s6	2.59E-44	-0.294076598	0.393	0.614	4.81E-40
		Srsf6	1.84E-72	-0.296175447	0.987	0.993	3.41E-68
		Smarca4	5.01E-68	-0.296847282	0.906	0.961	9.30E-64
		Eif5a	2.16E-91	-0.299220615	1	1	4.01E-87
		Idh2	9.89E-64	-0.300886989	0.925	0.963	1.83E-59
		Enpp2	2.40E-08	-0.310563498	0.24	0.323	4.46E-04
		Coro1c	1.79E-55	-0.313248087	0.845	0.918	3.31E-51
		Etv4	1.27E-60	-0.31358894	0.875	0.944	2.35E-56
		Fbn2	8.30E-48	-0.338599822	0.389	0.638	1.54E-43
		Bmp2	7.76E-50	-0.342019351	0.571	0.763	1.44E-45
		Pak3	2.52E-61	-0.346165467	0.282	0.56	4.68E-57
		Isl1	1.72E-65	-0.348645011	0.16	0.424	3.19E-61
		Vim	2.32E-92	-0.349296911	0.999	1	4.30E-88
		Epha3	3.29E-69	-0.351486355	0.248	0.547	6.09E-65
		Pa2g4	1.20E-68	-0.354654634	0.984	0.99	2.23E-64

	Cell differentiation	Leo1	1.26E-66	-0.355837873	0.69	0.84	2.34E-62
		Prmt1	6.64E-93	-0.36372117	0.997	0.998	1.23E-88
		Spry4	1.65E-67	-0.372462233	0.306	0.599	3.05E-63
		Prpf19	7.45E-101	-0.374476197	0.961	0.984	1.38E-96
		Tkt	6.67E-103	-0.380374454	0.968	0.993	1.24E-98
		Tnfrsf12a	3.06E-80	-0.384100696	0.99	0.998	5.67E-76
		Mmd	1.72E-54	-0.386986316	0.868	0.913	3.18E-50
		Map1b	3.08E-62	-0.388629079	0.967	0.961	5.71E-58
		Bcl6b	4.00E-66	-0.390503819	0.134	0.392	7.42E-62
		Crabp2	4.20E-37	-0.398452922	0.392	0.584	7.79E-33
		Tmsb4x	5.06E-82	-0.399435389	1	1	9.39E-78
		S100a10	7.22E-98	-0.414228428	1	1	1.34E-93
		Eif6	6.86E-159	-0.427249588	1	0.999	1.27E-154
		Anxa1	1.63E-51	-0.428779706	0.991	0.991	3.01E-47
		Ngf	2.27E-70	-0.503204548	0.477	0.721	4.21E-66
		Cd24a	2.90E-42	-0.537215887	0.141	0.333	5.38E-38
		Clic1	5.63E-176	-0.541062368	1	1	1.04E-171
		Uchl3	7.23E-182	-0.573807257	0.971	0.995	1.34E-177
		Wnt7b	3.03E-145	-0.620593146	0.292	0.704	5.62E-141
		Slc6a4	9.91E-103	-0.636633731	0.073	0.389	1.84E-98
		Rbpms2	8.18E-177	-0.646278485	0.784	0.939	1.52E-172
		Pthlh	7.07E-100	-0.678517695	0.598	0.807	1.31E-95
		Twist2	3.23E-206	-0.689308693	0.836	0.987	5.98E-202
		Hmga2	1.74E-227	-0.789674147	0.968	0.998	3.22E-223
		Clu	1.49E-123	-0.822352785	0.497	0.774	2.77E-119
		Hmg20a	2.08E-112	-0.827026013	0.561	0.778	3.85E-108
		Kras	3.82E-272	-0.868941928	0.963	0.998	7.08E-268
		Nefl	4.03E-164	-1.059020328	0.689	0.875	7.47E-160
		Areg	1.00E-197	-1.183864136	0.8	0.943	1.86E-193
		Xrcc5	2.04E-75	-0.417472747	0.615	0.804	3.78E-71
	Intracellular transport	March11	5.95E-52	-0.386333295	0.254	0.506	1.10E-47
	Splicesome	Snrpe	1.05E-133	-0.372168775	1	1	1.95E-129
		Sf3a3	1.60E-51	-0.268713267	0.849	0.913	2.97E-47
		Sf3a2	3.14E-41	-0.272079284	0.693	0.821	5.82E-37
		Ppil1	1.03E-50	-0.27972714	0.848	0.908	1.90E-46
		Sf3b2	3.82E-85	-0.280000632	0.997	0.998	7.08E-81
		Snrpb	1.39E-101	-0.285200951	1	1	2.57E-97
		Srpk1	8.10E-63	-0.304179973	0.879	0.94	1.50E-58
		Carhsp1	2.23E-60	-0.29349352	0.977	0.987	4.13E-56
		Snrpd3	1.15E-73	-0.306049419	0.99	0.993	2.13E-69
		Srsf3	6.66E-57	-0.308639606	0.993	0.998	1.23E-52
		Ddx41	6.87E-58	-0.317148515	0.787	0.889	1.27E-53
		Phf5a	6.38E-102	-0.340064168	0.987	0.996	1.18E-97
		Lsm2	1.74E-62	-0.340221285	0.936	0.959	3.23E-58
		Smn1	1.26E-69	-0.348483692	0.836	0.906	2.33E-65
		Snrpd1	6.26E-97	-0.363336006	0.995	0.997	1.16E-92
		Snrpa1	1.23E-106	-0.4035101	0.97	0.986	2.27E-102
		Ddx46	3.25E-98	-0.407124735	0.9	0.948	6.03E-94
		Ubtd1	4.66E-90	-0.401588963	0.933	0.977	8.65E-86
		Pgm1	6.44E-106	-0.481033209	0.605	0.841	1.19E-101
		Sh2d1b1	7.22E-41	-0.250090558	0.07	0.235	1.34E-36
		Lars	1.29E-33	-0.250463956	0.694	0.812	2.39E-29
		Pts	1.37E-40	-0.250548028	0.463	0.675	2.54E-36
		Rfc2	5.22E-35	-0.250791371	0.846	0.902	9.67E-31
		Gpd2	1.77E-34	-0.25139972	0.548	0.738	3.29E-30
		Psmb6	5.81E-79	-0.251431394	1	1	1.08E-74
		Cars	7.22E-27	-0.251588865	0.57	0.725	1.34E-22
		Ube2t	1.47E-39	-0.251836444	0.215	0.417	2.72E-35
		Mrps5	2.65E-46	-0.251997992	0.463	0.711	4.92E-42
		Foxl1	5.13E-58	-0.252712764	0.144	0.389	9.52E-54
		Dcun1d5	1.32E-62	-0.253364551	0.995	0.997	2.46E-58
		Orc2	1.17E-40	-0.253891214	0.437	0.664	2.17E-36
		Nup85	2.09E-35	-0.254286137	0.519	0.71	3.87E-31
		Tsta3	1.94E-51	-0.254409349	0.918	0.951	3.59E-47
		Ddx39	7.86E-95	-0.451387918	0.92	0.96	1.46E-90
		Pycrl	4.49E-38	-0.254768972	0.655	0.809	8.33E-34
		Psmc4	2.20E-54	-0.255245535	0.983	0.991	4.08E-50
		Denr	1.97E-45	-0.25546189	0.974	0.982	3.65E-41
		Lbr	3.06E-45	-0.255926058	0.334	0.581	5.68E-41
		Chac1	5.07E-28	-0.25613908	0.117	0.264	9.40E-24
		Riok1	2.86E-40	-0.256293027	0.455	0.67	5.30E-36
		Ddx18	3.29E-33	-0.256713881	0.814	0.876	6.11E-29
		Psmc6	2.96E-60	-0.257110881	0.988	0.997	5.49E-56
		Bop1	1.17E-47	-0.257580841	0.864	0.917	2.17E-43
		Elovl6	8.28E-38	-0.257873523	0.367	0.588	1.54E-33
		Lrwd1	3.62E-41	-0.258445621	0.421	0.648	6.72E-37
		Lactb2	2.64E-46	-0.25853693	0.303	0.542	4.90E-42
		Sms	7.74E-59	-0.25875579	0.668	0.862	1.44E-54
		Pin4	1.76E-49	-0.259000347	0.896	0.94	3.27E-45
		Mrpl34	3.65E-57	-0.25912894	0.979	0.989	6.78E-53
		Asf1a	2.03E-38	-0.25912894	0.588	0.77	3.77E-34
		Akr1b8	8.10E-45	-0.260103997	0.974	0.986	1.50E-40
		Tor1a	2.52E-45	-0.260391831	0.774	0.872	4.67E-41
		Sfxn1	6.49E-42	-0.261544001	0.838	0.904	1.20E-37
		Polr2c	6.57E-76	-0.261961394	0.995	0.998	1.22E-71
		Exosc2	1.11E-40	-0.263433885	0.375	0.599	2.06E-36

Eif1ax	5.49E-75	-0.263516041	0.997	0.998	1.02E-70
Uba1	8.26E-62	-0.264016702	0.983	0.991	1.53E-57
Eif3g	4.08E-83	-0.264339549	0.999	0.999	7.56E-79
Glrx5	1.10E-61	-0.264696596	0.992	0.998	2.05E-57
G3bp1	2.53E-63	-0.265368951	0.995	1	4.69E-59
Dtymk	8.20E-37	-0.26661611	0.738	0.839	1.52E-32
Mrps14	1.52E-70	-0.267167968	0.995	0.998	2.82E-66
Mak16	2.08E-42	-0.268037528	0.911	0.929	3.85E-38
Slk	9.85E-46	-0.26921189	0.437	0.687	1.83E-41
Fip1l1	5.86E-44	-0.269722346	0.909	0.944	1.09E-39
Ppat	6.80E-43	-0.270670389	0.404	0.636	1.26E-38
Cers6	1.27E-45	-0.271629056	0.405	0.648	2.36E-41
Polr2e	1.31E-64	-0.272042151	0.99	0.992	2.43E-60
Mrto4	3.13E-63	-0.272221513	0.973	0.988	5.80E-59
Ak2	2.14E-64	-0.27226929	0.983	0.991	3.97E-60
Acot9	1.86E-39	-0.272611302	0.911	0.927	3.45E-35
Nucks1	6.87E-43	-0.272888169	0.965	0.975	1.27E-38
Upf3b	2.88E-42	-0.273198488	0.656	0.807	5.33E-38
Rad18	8.33E-51	-0.275189658	0.261	0.509	1.54E-46
Prps1	1.44E-43	-0.275492072	0.561	0.755	2.67E-39
Shmt1	2.93E-46	-0.275653133	0.309	0.556	5.42E-42
Gcnt1	2.90E-59	-0.276378141	0.146	0.394	5.38E-55
Rrp15	9.55E-43	-0.276381004	0.844	0.9	1.77E-38
Rrp1b	6.92E-34	-0.276660187	0.387	0.586	1.28E-29
Psmb7	1.97E-81	-0.277161781	0.999	1	3.66E-77
Polr3k	1.13E-59	-0.277550997	0.886	0.942	2.09E-55
Mrpl21	5.03E-64	-0.277622403	0.967	0.983	9.32E-60
Mmp10	1.16E-33	-0.277876998	0.172	0.361	2.15E-29
Psma6	8.15E-77	-0.278071117	0.999	0.999	1.51E-72
Wdr74	1.31E-45	-0.2782943	0.728	0.846	2.43E-41
Eif2b3	7.90E-45	-0.279061462	0.603	0.777	1.46E-40
Tbl3	3.27E-47	-0.279450891	0.509	0.727	6.07E-43
Ola1	1.66E-67	-0.27956787	0.98	0.991	3.08E-63
Plpp2	1.38E-44	-0.280502967	0.651	0.817	2.56E-40
Trmt6	7.34E-39	-0.282164888	0.566	0.736	1.36E-34
Psmc1	6.03E-63	-0.282618877	0.983	0.986	1.12E-58
Slc25a13	6.40E-58	-0.283456159	0.172	0.419	1.19E-53
Med10	1.10E-62	-0.283736762	0.966	0.978	2.04E-58
Mrps18c	1.31E-57	-0.283789188	0.941	0.948	2.42E-53
Polr2j	2.42E-65	-0.284118088	0.988	0.988	4.49E-61
Gsr	1.42E-47	-0.284302005	0.872	0.93	2.62E-43
Nme6	7.34E-55	-0.284631549	0.246	0.496	1.36E-50
Ciapin1	6.27E-58	-0.285094411	0.935	0.959	1.16E-53
Gtf2a2	3.37E-70	-0.285136854	0.983	0.993	6.26E-66
Dph6	3.75E-51	-0.285761494	0.347	0.588	6.96E-47
Umps	1.03E-39	-0.285914849	0.525	0.708	1.92E-35
Rbm25	4.75E-68	-0.286054011	0.985	0.995	8.82E-64
Nedd8	4.19E-70	-0.286398404	0.958	0.979	7.77E-66
Got1	1.18E-50	-0.286953313	0.824	0.904	2.20E-46
Rnaseh2c	1.10E-62	-0.287124337	0.967	0.983	2.04E-58
Tk1	7.08E-37	-0.288888535	0.573	0.728	1.31E-32
Psmb3	3.64E-86	-0.289346859	1	1	6.75E-82
Glo1	9.53E-54	-0.289600524	0.732	0.867	1.77E-49
Mrpl49	2.18E-53	-0.289640964	0.802	0.898	4.05E-49
Abhd1	7.83E-42	-0.289943059	0.23	0.443	1.45E-37
Dhx34	2.82E-42	-0.29000212	0.179	0.388	5.24E-38
Pigf	2.68E-55	-0.290874134	0.479	0.726	4.98E-51
Adsl	2.80E-54	-0.29092331	0.785	0.895	5.19E-50
Cpsf2	8.03E-58	-0.291122047	0.782	0.895	1.49E-53
Mid1ip1	3.63E-47	-0.291452269	0.394	0.637	6.74E-43
Mrpl18	1.57E-68	-0.291730912	0.989	0.989	2.92E-64
Aig1	2.94E-26	-0.292469591	0.895	0.909	5.45E-22
Asl	3.05E-49	-0.292577925	0.511	0.725	5.65E-45
Prim1	4.19E-44	-0.293239171	0.278	0.514	7.78E-40
Txn2	7.53E-56	-0.293393657	0.501	0.74	1.40E-51
Qtrt1	8.58E-58	-0.294058647	0.424	0.687	1.59E-53
Mtch2	3.75E-94	-0.294514084	0.997	0.999	6.96E-90
Gsto1	1.27E-87	-0.296848743	0.997	1	2.36E-83
Dut	1.02E-48	-0.299259304	0.971	0.981	1.90E-44
Cirh1a	6.50E-58	-0.301141158	0.766	0.889	1.20E-53
Mdh2	5.26E-99	-0.301347892	1	1	9.75E-95
Itpa	6.75E-60	-0.301658989	0.932	0.95	1.25E-55
Polr3g	5.23E-67	-0.302269519	0.156	0.42	9.71E-63
Tyms	3.86E-42	-0.305237475	0.787	0.867	7.16E-38
Mrps10	6.43E-66	-0.305272119	0.877	0.941	1.19E-61
Gpt2	2.86E-54	-0.30555382	0.275	0.523	5.30E-50
Tyw5	6.87E-47	-0.306958591	0.392	0.619	1.27E-42
Ppp4c	2.54E-83	-0.307366409	0.984	0.993	4.71E-79
Ube2k	4.57E-82	-0.307451135	0.995	0.994	8.47E-78
Rps6ka1	4.64E-71	-0.307507414	0.185	0.467	8.61E-67
Eif3d	5.70E-91	-0.307643687	0.995	0.998	1.06E-86
Polr2g	5.60E-96	-0.308041703	0.991	0.998	1.04E-91
Nadk	6.25E-61	-0.308328454	0.919	0.945	1.16E-56
Gtpbp4	1.54E-56	-0.308440073	0.908	0.945	2.86E-52
Dnm1	6.03E-47	-0.311275412	0.718	0.844	1.12E-42
Tsn	2.59E-68	-0.311296183	0.987	0.993	4.80E-64

			Prdx4	1.72E-72	-0.312247692	0.985	0.995	3.19E-68
			Polr3h	1.37E-53	-0.313419688	0.75	0.865	2.54E-49
		Metabolic process	Park7	9.71E-64	-0.314174692	0.756	0.889	1.80E-59
			Fen1	4.18E-53	-0.314436296	0.322	0.581	7.75E-49
			Tmem199	1.14E-65	-0.314502676	0.663	0.849	2.11E-61
			Gtf3c6	1.20E-64	-0.31572027	0.854	0.919	2.22E-60
			Fkbp1a	7.96E-125	-0.315910067	1	1	1.48E-120
			Psma3	5.18E-125	-0.316651439	1	1	9.60E-121
			Vps37b	1.36E-47	-0.318144177	0.364	0.614	2.52E-43
			Ruvbl2	4.50E-62	-0.31908042	0.897	0.942	8.35E-58
			Xrn2	7.22E-78	-0.31937725	0.943	0.975	1.34E-73
			Rfc5	1.01E-48	-0.319385788	0.541	0.745	1.87E-44
			Esd	2.33E-63	-0.320740657	0.978	0.991	4.33E-59
			Sprr1a	8.01E-40	-0.320779428	0.154	0.358	1.49E-35
			Psma2	1.11E-97	-0.321368591	1	1	2.05E-93
			Ebna1bp2	1.27E-73	-0.321494249	0.989	0.991	2.36E-69
			Hint1	6.31E-112	-0.322026615	1	1	1.17E-107
			Eif1ad	4.09E-73	-0.322693296	0.849	0.925	7.59E-69
			Mrps25	1.85E-72	-0.322932519	0.921	0.957	3.43E-68
			Smg6	3.68E-39	-0.323067096	0.746	0.844	6.83E-35
			Txn1	3.84E-104	-0.323291233	1	1	7.12E-100
			Peak1	7.18E-43	-0.324068503	0.665	0.813	1.33E-38
			Psmd14	1.14E-84	-0.324571067	0.981	0.99	2.11E-80
			Rbp2	2.35E-56	-0.325785247	0.238	0.493	4.36E-52
			Ddx27	3.37E-68	-0.326663398	0.829	0.912	6.25E-64
			Pcbd1	3.08E-60	-0.327261355	0.292	0.56	5.71E-56
			Aprt	6.19E-83	-0.327872983	0.999	1	1.15E-78
			Hsd17b12	1.72E-87	-0.330038291	0.964	0.989	3.19E-83
			Ndufb9	8.52E-51	-0.330791103	1	1	1.58E-46
			Cndp2	4.51E-48	-0.332359071	0.809	0.894	8.37E-44
			Dbi	7.00E-92	-0.33278951	0.999	1	1.30E-87
			Ndufa8	5.72E-109	-0.332900301	0.995	1	1.06E-104
			Syncrip	7.95E-63	-0.33338231	0.527	0.75	1.47E-58
			Rpf2	2.48E-51	-0.335994315	0.666	0.808	4.61E-47
			Imp4	1.56E-69	-0.337938905	0.826	0.921	2.89E-65
			Odc1	4.32E-49	-0.340933839	0.766	0.849	8.01E-45
			Traf5	5.55E-65	-0.341098407	0.245	0.522	1.03E-60
			Plau	5.25E-36	-0.343230969	0.393	0.597	9.73E-32
			Ly6e	5.14E-50	-0.347998718	0.999	1	9.54E-46
			Wdr12	2.47E-61	-0.348526083	0.564	0.772	4.58E-57
			Psmd7	2.03E-95	-0.349328872	0.999	1	3.76E-91
			Cops5	3.04E-109	-0.351058973	0.98	0.991	5.63E-105
			Farsb	1.12E-67	-0.353934658	0.674	0.844	2.07E-63
			Orc6	1.21E-64	-0.354139432	0.89	0.94	2.25E-60
			Ssrp1	2.44E-111	-0.358461327	0.996	0.999	4.53E-107
			Pter	3.02E-67	-0.362325987	0.315	0.595	5.61E-63
			Dhfr	3.20E-52	-0.366525932	0.451	0.684	5.94E-48
			Dpy30	1.41E-67	-0.36927289	0.925	0.943	2.61E-63
			Acsbg1	3.28E-72	-0.369583687	0.21	0.503	6.08E-68
			Rrp9	1.22E-74	-0.371507009	0.748	0.883	2.26E-70
			Psmc2	3.83E-84	-0.372267651	0.977	0.989	7.10E-80
			Psat1	2.35E-75	-0.372794131	0.92	0.966	4.37E-71
			Dkc1	9.43E-67	-0.378240622	0.824	0.904	1.75E-62
			Mgst2	1.67E-76	-0.380919461	0.573	0.816	3.09E-72
			Uchl5	1.01E-78	-0.384913817	0.804	0.904	1.88E-74
			Cyb5b	7.82E-84	-0.385751147	0.899	0.944	1.45E-79
			Pop5	5.79E-70	-0.388052252	0.497	0.739	1.07E-65
			Eif5	1.17E-107	-0.388553285	0.999	1	2.17E-103
			St3gal6	1.24E-42	-0.390547546	0.13	0.321	2.29E-38
			Cd9	8.12E-79	-0.392506454	0.999	1	1.51E-74
			Wdr18	1.35E-92	-0.394684296	0.891	0.941	2.51E-88
			Prkg2	5.42E-34	-0.397800788	0.755	0.822	1.01E-29
			Aimp2	4.28E-94	-0.405433227	0.91	0.945	7.94E-90
			Rars	3.34E-86	-0.406435085	0.924	0.954	6.20E-82
			Mrps6	2.75E-89	-0.40818056	0.983	0.993	5.10E-85
			Bag2	2.69E-78	-0.408241367	0.654	0.836	4.99E-74
			Zdhhc12	1.94E-88	-0.409478472	0.613	0.834	3.60E-84
			Alad	2.82E-97	-0.409736069	0.919	0.965	5.23E-93
			Agpat2	4.27E-51	-0.409945011	0.496	0.698	7.92E-47
			Eif2s1	2.84E-116	-0.410051967	0.982	0.993	5.27E-112
			Acot7	7.89E-96	-0.410755202	0.955	0.978	1.46E-91
			Gsto2	4.23E-71	-0.411697539	0.117	0.382	7.84E-67
			Polr2f	1.88E-134	-0.413246726	1	1	3.48E-130
			Rnf183	2.52E-95	-0.413325407	0.079	0.38	4.67E-91
			Dnajc2	4.05E-93	-0.414997817	0.949	0.974	7.50E-89
			Dusp14	9.07E-82	-0.415565841	0.395	0.695	1.68E-77
			Mrpl20	9.15E-143	-0.41633514	0.983	0.995	1.70E-138
			Ormdl2	4.03E-105	-0.416965616	0.648	0.873	7.48E-101
			Degs1	1.54E-115	-0.41817994	0.965	0.993	2.86E-111
			Eif3h	8.45E-99	-0.419726808	1	1	1.57E-94
			Ptges	5.54E-75	-0.421416047	0.838	0.941	1.03E-70
			Acsl6	1.09E-103	-0.42158206	0.21	0.563	2.01E-99
			Chst2	3.50E-68	-0.432203318	0.322	0.611	6.49E-64
			Ino80c	1.24E-99	-0.436353924	0.576	0.815	2.30E-95
			Hmgn5	2.58E-84	-0.437002688	0.878	0.927	4.79E-80
			Ptgs1	2.29E-51	-0.439821652	0.373	0.604	4.24E-47

	Psmd1	4.28E-129	-0.441469129	0.991	0.996	7.93E-125
	Slc16a11	7.61E-69	-0.441671586	0.105	0.362	1.41E-64
	Ttll12	7.92E-96	-0.448730183	0.682	0.865	1.47E-91
	Lap3	5.15E-134	-0.450515432	0.976	0.989	9.55E-130
	Prdx5	1.01E-39	-0.451462165	0.999	1	1.88E-35
	Obfc1	2.75E-97	-0.454522766	0.447	0.749	5.10E-93
	Nppb	7.00E-30	-0.457658869	0.056	0.184	1.30E-25
	Ppa1	4.68E-139	-0.486136215	0.991	0.995	8.69E-135
	Hao1	3.03E-100	-0.487678668	0.156	0.494	5.63E-96
	Nhp2	6.04E-139	-0.487786474	0.997	1	1.12E-134
	Psma7	1.72E-217	-0.503780472	1	1	3.19E-213
	Qdpr	1.80E-122	-0.508954677	0.772	0.915	3.33E-118
	Sec11c	1.54E-121	-0.511404344	0.92	0.974	2.85E-117
	Pycr2	1.42E-141	-0.52466377	0.947	0.976	2.63E-137
	Lyar	7.05E-126	-0.524841404	0.977	0.986	1.31E-121
	Gale	1.12E-128	-0.543210094	0.447	0.775	2.08E-124
	Pla2g7	1.56E-89	-0.562575688	0.71	0.857	2.88E-85
	Galnt1	1.72E-148	-0.586330207	0.786	0.933	3.19E-144
	Adh7	1.61E-56	-0.589049115	0.069	0.273	2.99E-52
	Uchl1	1.27E-84	-0.599090087	0.583	0.777	2.35E-80
	Mgst3	1.69E-118	-0.761236261	0.714	0.866	3.13E-114
	Upp1	4.67E-156	-0.811972839	0.852	0.946	8.67E-152
	Dtx2	9.37E-148	-0.868416803	0.727	0.888	1.74E-143
	Crhbp	9.04E-135	-0.940225472	0.519	0.781	1.68E-130
	Ctsl	7.35E-263	-0.940615432	1	1	1.36E-258
	Crabp1	3.19E-236	-1.021249658	0.945	0.999	5.92E-232
	Tmpo	1.76E-49	-0.252768488	0.787	0.892	3.26E-45
	Med19	6.00E-43	-0.256805844	0.581	0.777	1.11E-38
	Mllt11	2.33E-27	-0.258428625	0.585	0.728	4.33E-23
	Nqo2	9.96E-32	-0.268247202	0.674	0.795	1.85E-27
	Pum3	1.76E-47	-0.274160764	0.828	0.905	3.26E-43
	Eif5b	3.82E-78	-0.276263052	0.995	0.999	7.08E-74
	Pcbp4	8.72E-49	-0.283439189	0.927	0.953	1.62E-44
	Sltm	8.46E-66	-0.310087878	0.92	0.952	1.57E-61
	Lrrfip1	4.53E-53	-0.310631052	0.528	0.754	8.40E-49
	Ppp1r7	1.63E-68	-0.324278203	0.788	0.884	3.02E-64
	Sigirr	5.01E-67	-0.335515846	0.14	0.401	9.30E-63
	Lxn	2.04E-39	-0.339237546	0.887	0.912	3.79E-35
	Cdc37	7.73E-171	-0.34903652	1	1	1.43E-166
	Pfdn4	1.12E-107	-0.365899911	0.986	0.995	2.09E-103
	Cisd1	1.67E-98	-0.377754835	0.969	0.993	3.10E-94
	Pfdn2	4.61E-129	-0.394004944	0.998	0.998	8.55E-125
	Lgals7	1.80E-167	-1.055293488	0.764	0.918	3.34E-163
	Dynap	2.15E-135	-0.988003595	0.348	0.686	3.99E-131
Telomere maintainence	Cct2	9.33E-114	-0.3182767	1	1	1.73E-109
	Cct6a	2.84E-87	-0.35307839	0.998	0.996	5.26E-83
	Tcp1	7.97E-117	-0.335884596	1	1	1.48E-112
	Cct5	2.34E-89	-0.284205231	1	1	4.34E-85
	Cct7	1.90E-92	-0.307680967	1	1	3.52E-88
Cell component organization or biogenesis	Mpp6	7.47E-66	-0.337871393	0.769	0.901	1.38E-61
	C1ql2	2.80E-71	-0.596445558	0.059	0.298	5.20E-67
	Ntm	1.79E-99	-0.519042246	0.091	0.406	3.33E-95
	Pcolce2	1.88E-74	-0.491355944	0.386	0.657	3.48E-70
	Actr1b	1.98E-163	-0.483086852	0.981	0.997	3.67E-159
	Ak6	4.63E-135	-0.468647865	0.949	0.986	8.58E-131
	Nol7	7.86E-109	-0.381340995	0.979	0.991	1.46E-104
	Mrps22	1.76E-82	-0.376830083	0.664	0.852	3.26E-78
	Bzw1	7.11E-56	-0.290146048	0.984	0.988	1.32E-51
	Dnttip1	1.47E-47	-0.289014518	0.681	0.82	2.73E-43
	Tmem18	5.83E-63	-0.347018416	0.322	0.599	1.08E-58
	Tmem183a	1.27E-75	-0.347048049	0.833	0.923	2.36E-71
	Tmem200a	9.82E-51	-0.28197523	0.176	0.408	1.82E-46
	Chic1	2.10E-52	-0.271211239	0.272	0.529	3.90E-48
	Mrpl28	8.84E-70	-0.263523315	0.987	0.993	1.64E-65
	Hmmr	4.61E-40	-0.259816562	0.256	0.485	8.55E-36
	Ankrd9	1.08E-50	-0.251986835	0.184	0.417	2.00E-46
	Ggct	7.79E-90	-0.562394682	0.334	0.642	1.45E-85
	Timm9	7.34E-37	-0.263715565	0.714	0.832	1.36E-32
	Noc4l	5.69E-39	-0.269164091	0.494	0.693	1.06E-34
	Tomm40	4.17E-57	-0.270488396	0.989	0.991	7.74E-53
	Grpel2	4.00E-50	-0.274012955	0.463	0.713	7.42E-46
	Dek	2.92E-37	-0.276027138	0.926	0.945	5.41E-33
	Pfdn6	1.08E-66	-0.283864167	0.986	0.995	2.00E-62
	Tmem201	3.71E-50	-0.285433269	0.18	0.407	6.88E-46
	Ndufaf2	1.95E-57	-0.291067725	0.914	0.95	3.62E-53
	Tomm34	1.04E-61	-0.30165064	0.702	0.861	1.93E-57
	Elmo2	5.26E-55	-0.306705156	0.342	0.597	9.76E-51
	Nup35	4.44E-50	-0.307551263	0.371	0.611	8.23E-46
	Atad2	2.11E-34	-0.3087358	0.336	0.545	3.91E-30
	Mtx2	1.21E-74	-0.312554183	0.965	0.979	2.24E-70
	Nop58	1.45E-52	-0.312806741	0.934	0.954	2.69E-48
	Hmgn3	1.18E-48	-0.328398676	0.943	0.954	2.19E-44
	Serbp1	1.65E-114	-0.331385377	1	1	3.05E-110
	Ehd4	3.39E-64	-0.350728595	0.67	0.859	6.28E-60
	Rab3ip	9.14E-62	-0.354894101	0.352	0.62	1.70E-57
	Emp3	2.82E-117	-0.37101052	1	1	5.24E-113

	Arpc5	3.05E-95	-0.37960108	0.99	0.997	5.65E-91
	Nolc1	3.64E-71	-0.387590362	0.927	0.948	6.75E-67
	Kctd4	1.33E-70	-0.404360541	0.122	0.392	2.46E-66
	Gchfr	7.06E-89	-0.42790824	0.197	0.526	1.31E-84
	Laptm4b	3.15E-78	-0.430590662	0.934	0.969	5.84E-74
	Timm17a	2.64E-170	-0.470322566	0.997	0.999	4.90E-166
	Laptm5	9.19E-49	-0.476635928	0.078	0.267	1.70E-44
	Plec	5.04E-89	-0.485205248	0.941	0.967	9.35E-85
	Tgfbi	6.68E-75	-0.506629081	0.897	0.964	1.24E-70
	Bok	1.93E-114	-0.641103589	0.732	0.87	3.59E-110
	Kcnn4	3.01E-192	-0.766403611	0.632	0.897	5.59E-188
	Trbc2	1.10E-69	-0.781858908	0.43	0.645	2.03E-65
	Krt18	7.57E-178	-1.171702239	0.802	0.923	1.40E-173
	Tmem173	5.09E-34	-0.331942024	0.809	0.861	9.44E-30
	Penk	1.59E-91	-0.969005798	0.25	0.572	2.95E-87
	Asb6	8.81E-43	-0.255768419	0.364	0.588	1.63E-38
	Rangap1	2.19E-42	-0.27746073	0.794	0.871	4.07E-38
	Rasa2	7.53E-51	-0.307204743	0.366	0.623	1.40E-46
	Islr	1.32E-139	0.664574286	0.354	0.05	2.46E-135
	Pdap1	6.17E-102	-0.341933378	1	0.999	1.14E-97
	Rcan2	1.51E-68	-0.360336788	0.093	0.341	2.79E-64
Signal transduction	Btbd11	1.51E-94	-0.379059784	0.219	0.56	2.79E-90
	Calcb	7.87E-22	-0.387473264	0.049	0.147	1.46E-17
	Gprc5a	1.33E-81	-0.456723722	0.41	0.705	2.46E-77
	Arhgdib	6.98E-94	-0.461110752	0.778	0.899	1.29E-89
	Mpzl1	1.84E-117	-0.483118686	0.902	0.971	3.42E-113
	Itgbl1	8.28E-75	-0.503956079	0.204	0.503	1.54E-70
	Rgs17	8.00E-103	-0.508924164	0.692	0.881	1.48E-98
	Anks1b	7.68E-160	-0.613659897	0.203	0.648	1.42E-155
	Ttc1	4.07E-53	-0.262833018	0.857	0.923	7.55E-49
	Stom	8.05E-61	-0.371668094	0.579	0.786	1.49E-56
	Slc14a1	6.05E-143	-0.7600095	0.299	0.694	1.12E-138
	Osbpl6	8.50E-52	-0.250575604	0.131	0.351	1.58E-47
	Atp6v1c1	3.06E-51	-0.256750818	0.866	0.943	5.68E-47
	Slc25a3	7.95E-106	-0.263947904	1	1	1.47E-101
	Slc20a2	8.80E-49	-0.266132363	0.363	0.622	1.63E-44
	Vta1	5.24E-62	-0.312788916	0.785	0.887	9.72E-58
Ion transport	Nxt1	1.01E-52	-0.31491142	0.673	0.83	1.88E-48
	Timm8b	9.92E-109	-0.318128607	0.997	1	1.84E-104
	Piezo2	6.94E-69	-0.328087071	0.177	0.458	1.29E-64
	Kcnk3	1.22E-44	-0.363699545	0.081	0.265	2.26E-40
	Ap1s1	1.62E-94	-0.369722367	0.989	0.99	3.01E-90
	Slc30a4	9.17E-86	-0.464314632	0.664	0.854	1.70E-81
	Cytip	8.93E-101	-0.4247648	0.178	0.528	1.66E-96
	Cnih4	8.90E-191	-0.484716976	0.996	0.999	1.65E-186
	Slc9a2	1.28E-125	-0.499608381	0.197	0.608	2.37E-121
	Anxa5	5.30E-122	-0.481791946	1	1	9.83E-118
Regulation of cell communication	Neto2	1.71E-159	-0.805072423	0.65	0.863	3.17E-155
	Fam110c	3.04E-91	-0.446462088	0.186	0.517	5.65E-87
	Grina	8.30E-47	-0.314774877	0.9	0.95	1.54E-42
	Txnl1	1.78E-77	-0.253798031	0.997	0.999	3.31E-73
Redox homeostasis	Sh3bgrl3	2.81E-143	-0.488057044	1	1	5.21E-139
	Txndc9	1.45E-42	-0.280412828	0.906	0.933	2.68E-38
	Antxr2	2.48E-33	-0.295285947	0.698	0.802	4.60E-29
Cell transport	Trappc1	5.59E-62	-0.324332312	0.484	0.729	1.04E-57
	Exoc3l4	6.87E-64	-0.345701218	0.31	0.586	1.27E-59
	Trappc13	9.62E-40	-0.255123552	0.494	0.702	1.78E-35
	Klra4	4.13E-129	-0.832365055	0.138	0.532	7.66E-125
	Gm26917	0	-0.798779256	0.947	1	0
	Pxdc1	4.56E-178	-0.752725715	0.881	0.971	8.45E-174
	Gm12603	9.22E-129	-0.678801843	0.199	0.592	1.71E-124
	Fam84a	7.26E-122	-0.557977502	0.193	0.593	1.35E-117
	10039M20Ril	3.45E-148	-0.542930215	0.244	0.699	6.40E-144
	Card19	3.98E-109	-0.499816885	0.86	0.94	7.38E-105
	AA467197	3.03E-47	-0.480326236	0.772	0.824	5.62E-43
	Rps4l	3.28E-112	-0.468078939	0.875	0.983	6.08E-108
	930523C07Ri	5.20E-80	-0.453183377	0.29	0.605	9.65E-76
	Rwdd4a	5.11E-103	-0.450597331	0.889	0.954	9.47E-99
	200002D01Ri	3.42E-79	-0.434884264	0.332	0.657	6.35E-75
	Sssca1	9.21E-90	-0.417659883	0.904	0.954	1.71E-85
	Fam189b	3.12E-66	-0.385646861	0.658	0.828	5.79E-62
	Adss	1.11E-95	-0.380981388	0.95	0.98	2.06E-91
	430016H08Ri	2.32E-57	-0.359725988	0.688	0.823	4.31E-53
	Fam216a	3.37E-54	-0.346858361	0.671	0.815	6.25E-50
	110008F13Ri	3.30E-127	-0.344256905	0.998	1	6.12E-123
	110002H16Ri	6.27E-75	-0.341701423	0.327	0.636	1.16E-70
	Reep5	5.32E-95	-0.338684469	0.997	1	9.86E-91
	700094K13Ri	1.97E-63	-0.338321815	0.431	0.702	3.66E-59
	Tex30	3.27E-61	-0.336120094	0.599	0.8	6.07E-57
	Fh1	6.14E-63	-0.334553385	0.923	0.942	1.14E-58
Unclear function	Ccdc109b	9.00E-40	-0.318870266	0.899	0.915	1.67E-35
	Rn18s-rs5	3.80E-79	-0.31318817	0.185	0.488	7.05E-75
	110008P14Ri	3.50E-55	-0.30572584	0.923	0.961	6.49E-51
	Eml5	1.35E-78	-0.304548012	0.123	0.408	2.51E-74
	410131K14Ri	3.60E-52	-0.301484432	0.324	0.583	6.68E-48
	010204K13Ri	1.18E-50	-0.300182637	0.397	0.657	2.20E-46

		Surf2	1.40E-49	-0.292334956	0.487	0.713	2.60E-45
		Cdkn2aipnl	2.10E-52	-0.289215149	0.517	0.749	3.89E-48
		810417H13Ri	1.06E-24	-0.287220739	0.577	0.681	1.96E-20
		Zfp428	2.09E-50	-0.286285248	0.406	0.649	3.88E-46
		810025M15R	1.35E-54	-0.285094102	0.879	0.934	2.50E-50
		Lyrm9	2.12E-60	-0.280955968	0.19	0.449	3.93E-56
		Cdk2ap2	1.62E-40	-0.277087714	0.965	0.986	3.01E-36
		Fam64a	9.11E-43	-0.275053709	0.246	0.48	1.69E-38
		130401M01R	2.68E-10	-0.272490422	0.902	0.904	4.97E-06
		Gm28875	5.91E-39	-0.272296871	0.212	0.414	1.10E-34
		Utp11l	6.44E-56	-0.272181649	0.959	0.98	1.19E-51
		700037H04Ri	1.38E-46	-0.26932887	0.72	0.853	2.56E-42
		430005L14Ri	1.14E-37	-0.264120784	0.445	0.657	2.11E-33
		Ccdc34	1.16E-38	-0.26244847	0.709	0.822	2.15E-34
		2310061I04Ril	1.06E-38	-0.257025427	0.46	0.671	1.97E-34
		Gm16058	3.21E-55	-0.253180371	0.067	0.267	5.94E-51
		Zfp706	3.57E-55	-0.252176808	0.997	0.998	6.63E-51
		Tmem206	2.39E-55	-0.250150584	0.182	0.424	4.43E-51

Supplementary Table 2. Differential Gene Expression Analysis from scRNA-seq of SP relative to non-SP cells in tumor KPCC-840

	Function Group	Gene	p value	average logFC	pct.1	pct.2	adusted p value
Upregulated genes	Negative regulation of developmental process	Tmem176a	1.23E-68	0.309732589	0.524	0.328	3.82E-64
		Nppc	1.76E-10	0.251018735	0.395	0.328	5.46E-06
		Thy1	8.22E-89	0.364744726	0.833	0.654	2.55E-84
		Srgn	2.06E-43	0.26089578	0.222	0.109	6.40E-39
		Mdk	6.47E-69	0.308313567	0.588	0.412	2.01E-64
		Ccl2	1.72E-49	0.525097147	0.914	0.821	5.34E-45
		Dlk1	3.56E-142	0.698191699	0.532	0.255	1.11E-137
		Mest	1.23E-36	0.427740582	0.318	0.191	3.82E-32
		Ctgf	7.59E-33	0.302764825	0.441	0.32	2.36E-28
		Plk2	7.45E-68	0.290813537	0.906	0.8	2.31E-63
		Rgs2	3.50E-86	0.274100824	0.763	0.606	1.09E-81
		Tagln	6.03E-22	0.267392264	0.385	0.295	1.87E-17
		Spp1	6.66E-23	0.296211811	1	1	2.07E-18
		Tmem176b	2.79E-65	0.308515486	0.556	0.367	8.67E-61
	Skeletal muscle development	Egr1	4.08E-50	0.2722268	0.898	0.798	1.27E-45
		Nr4a1	1.01E-56	0.32363452	0.767	0.647	3.15E-52
		Fos	9.32E-64	0.31363904	0.918	0.804	2.90E-59
		Dcn	6.99E-87	0.371839589	0.983	0.966	2.17E-82
	Response to wounding	Pdpn	1.63E-81	0.306098093	0.95	0.906	5.05E-77
		S100a8	8.34E-16	0.38985502	0.26	0.188	2.59E-11
		Tfpi2	2.58E-79	0.324426195	0.844	0.708	8.01E-75
		Hmox1	8.41E-77	0.435023137	0.93	0.837	2.61E-72
		Timp1	1.27E-53	0.284683517	0.995	0.991	3.93E-49
	Extracellular matrix organization	Fbln2	6.91E-122	0.560019288	0.777	0.588	2.15E-117
		Mmp2	2.30E-46	0.268264175	0.871	0.77	7.15E-42
		Bgn	2.23E-111	0.261351349	0.848	0.704	6.93E-107
		Lum	7.63E-57	0.267842625	0.929	0.856	2.37E-52
		Mfap4	1.17E-81	0.304981072	0.367	0.166	3.63E-77
	Chemokine mediated signaling pathway	Cxcl2	1.49E-27	0.463414558	0.74	0.624	4.62E-23
		Ccl7	4.60E-83	0.433021402	0.774	0.565	1.43E-78
		Cxcl1	2.02E-122	0.705358178	0.931	0.738	6.26E-118
	Chemokine production	Ptgs2	1.30E-39	0.261178355	0.759	0.656	4.05E-35
		Tslp	1.11E-13	0.255949572	0.4	0.339	3.46E-09
	Metabolic processes	Cilp	8.21E-77	0.283401515	0.434	0.218	2.55E-72
		Ppp1r15a	7.49E-112	0.327989105	0.933	0.84	2.33E-107
		Il11	7.91E-83	0.375505236	0.553	0.364	2.46E-78
		Pld3	1.77E-75	0.269341148	0.87	0.78	5.51E-71
		Agtr2	4.32E-46	0.310715309	0.398	0.253	1.34E-41
		Fabp4	8.63E-25	0.347655974	0.275	0.186	2.68E-20
		Rcn3	1.41E-140	0.371971618	0.948	0.81	4.38E-136
		Spock3	4.36E-121	0.353394437	0.561	0.316	1.35E-116
		Cck	1.67E-116	0.972816664	0.287	0.082	5.20E-112
		Gsto1	1.03E-76	0.337369557	0.945	0.908	3.21E-72
		Mgst1	3.20E-84	0.320640407	0.497	0.275	9.93E-80
	Oxidative stress response	Mt2	1.02E-42	0.519243575	0.993	0.978	3.16E-38
		Mt1	1.23E-27	0.466937985	0.988	0.979	3.81E-23
		Sod3	2.93E-52	0.334917499	0.448	0.291	9.10E-48
	Signal transduction	Penk	1.53E-37	0.381636347	0.394	0.264	4.75E-33
		Gem	2.14E-78	0.30050944	0.775	0.621	6.66E-74
		Npy	7.84E-30	0.277473644	0.168	0.083	2.44E-25
		Ier3	7.68E-28	0.268883101	0.958	0.915	2.39E-23
	Cellular components and organization	Map1lc3b	1.28E-32	0.276468923	0.976	0.954	3.96E-28
		Aqp1	9.22E-57	0.511951574	0.402	0.238	2.86E-52
		Tmsb10	1.48E-73	0.257390528	0.993	0.982	4.59E-69
	Unclear functions	H19	1.91E-37	0.373636687	0.332	0.201	5.93E-33
		Dnajb9	1.40E-70	0.26900284	0.906	0.827	4.36E-66
		Fam213a	2.37E-20	0.259641865	0.515	0.444	7.35E-16
Downregulated genes	Cell cycle processes	Cdk1	3.56E-37	-0.315051886	0.442	0.553	1.11E-32
		Lmna	1.37E-76	-0.262429542	0.975	0.961	4.24E-72
		Cks1b	3.09E-85	-0.45096794	0.858	0.895	9.59E-81
		Cdc20	2.05E-39	-0.255850426	0.455	0.575	6.38E-35
		Krt18	5.52E-29	-0.265460326	0.587	0.731	1.71E-24
		Ranbp1	3.08E-108	-0.332735283	0.958	0.953	9.57E-104
		Phgdh	3.15E-58	-0.287831173	0.746	0.825	9.77E-54
		Birc5	1.60E-38	-0.253568243	0.337	0.472	4.98E-34
		Tubb6	9.11E-50	-0.319966288	0.913	0.925	2.83E-45
		Cenpa	8.15E-30	-0.266233415	0.526	0.621	2.53E-25
		Hmga2	6.24E-82	-0.362800901	0.841	0.864	1.94E-77

	Cell differentiation	Nefl	5.60E-41	-0.356507134	0.58	0.713	1.74E-36
		Plet1	2.35E-56	-0.331509144	0.239	0.403	7.31E-52
		Kras	2.70E-74	-0.387899739	0.796	0.849	8.39E-70
		Krt8	2.50E-37	-0.713663418	0.125	0.235	7.77E-33
		Acta1	1.91E-07	-0.607910918	0.108	0.149	0.005940658
		Col18a1	2.74E-09	-0.388881146	0.548	0.588	8.52E-05
		Il33	1.37E-43	-0.297219692	0.563	0.702	4.25E-39
		Dkk2	1.57E-30	-0.283823426	0.828	0.845	4.88E-26
		Crip2	1.20E-50	-0.283220315	0.601	0.762	3.72E-46
		Hmgn1	2.53E-105	-0.28474691	0.974	0.968	7.86E-101
	Developmental process	Xist	1.51E-35	-0.301406774	0.912	0.876	4.68E-31
		Ybx1	2.88E-127	-0.305708773	0.95	0.939	8.93E-123
	Cellular component and organization	Cnih4	1.01E-77	-0.252828709	0.885	0.889	3.15E-73
		Tmsb4x	9.64E-126	-0.355772866	0.999	1	2.99E-121
		Kcnn4	1.36E-75	-0.285261367	0.489	0.655	4.22E-71
		Slc14a1	8.74E-23	-0.262081531	0.582	0.666	2.71E-18
		Msln	6.67E-48	-0.341753904	0.416	0.568	2.07E-43
	Metbaolic process	mt-Nd1	1.78E-13	-0.260984422	0.991	0.976	5.52E-09
		Cst6	3.59E-32	-0.299757445	0.686	0.787	1.11E-27
		Psip1	2.90E-105	-0.272304785	0.655	0.785	9.00E-101
		Mgst3	7.01E-49	-0.268261678	0.563	0.679	2.18E-44
		Chrna1	4.81E-54	-0.368119956	0.578	0.707	1.49E-49
		Sec11c	3.85E-66	-0.395647061	0.818	0.847	1.19E-61
	Unclear functions	Zfp706	1.92E-92	-0.261626589	0.912	0.911	5.98E-88
		Gm26917	5.48E-15	-0.266614998	0.703	0.768	1.70E-10
		Dynap	2.48E-79	-0.2695101	0.323	0.525	7.69E-75
		Rgs5	5.48E-11	-0.51173814	0.151	0.199	1.70E-06

Supplementary Table 3. Differential Gene Expression Analysis from scRNA-seq of SP relative to non-SP cells in tumor KPCC-00

	Function Group	Gene	p value	average logFC	pct.1	pct.2	adusted p value
Upregulated genes	Negative regulation of muscle differentiation	Tmem119	8.66E-09	0.317572326	0.282	0.172	1.37E-04
		Id2	7.12E-07	0.289624189	0.484	0.404	0.011264573
		Rgs2	4.01E-26	0.482285344	0.791	0.601	6.34E-22
		Pi16	2.73E-08	0.385405219	0.141	0.056	4.32E-04
	Negative regulation of developmental processes	Ccl2	3.80E-07	0.29073635	0.95	0.817	0.006006033
		Aspn	4.08E-19	0.527930532	0.468	0.254	6.46E-15
		Rgcc	9.37E-10	0.320295324	0.687	0.562	1.48E-05
		Tspo	8.85E-08	0.252019463	0.998	1	0.001399052
		Mmp9	9.42E-07	0.327013827	0.22	0.128	0.014903006
		Ptn	5.52E-21	0.522610945	0.666	0.452	8.73E-17
		Klf4	3.60E-12	0.319621514	0.705	0.576	5.70E-08
		Id1	7.18E-12	0.342065744	0.739	0.591	1.13E-07
		Strap	5.57E-19	0.251410456	0.973	0.936	8.80E-15
		Fos	2.55E-19	0.465490955	0.87	0.716	4.03E-15
		Dap	4.10E-07	0.256928241	0.998	0.994	0.006477308
		Serpinf1	2.05E-16	0.319796212	0.989	0.97	3.24E-12
		Dlk1	1.94E-22	0.317624608	0.819	0.529	3.08E-18
		Myc	3.10E-12	0.346888413	0.929	0.824	4.91E-08
	Response to wounding	Hbegf	4.16E-13	0.281128258	0.929	0.827	6.59E-09
		Cd9	2.62E-18	0.260737069	0.998	0.991	4.14E-14
		Serping1	2.35E-12	0.406998845	0.567	0.385	3.71E-08
		Lrp1	1.85E-12	0.262542974	0.905	0.81	2.93E-08
	Response to dexamethasone	Ddit4	2.23E-07	0.280472477	0.514	0.406	0.00352809
		Aqp1	2.89E-39	0.854375654	0.664	0.349	4.57E-35
		Fibin	2.69E-08	0.323721644	0.257	0.145	4.25E-04
	Regulation of hematopoeitic stem cell proliferation	Cxcl1	9.18E-19	0.545602835	0.953	0.778	1.45E-14
		Ace	1.08E-15	0.329599966	0.499	0.309	1.71E-11
		Pim1	3.11E-21	0.383095761	0.794	0.587	4.92E-17
	Response to corticotropin-releasing hormone stimulus	Nr4a2	8.36E-19	0.498062029	0.731	0.538	1.32E-14
		Nr4a1	2.98E-26	0.536553228	0.828	0.601	4.71E-22
	Chemokine mediated signaling pathway	Ccl7	5.81E-26	0.59732522	0.867	0.59	9.19E-22
		Cxcl2	3.57E-22	0.773229791	0.85	0.593	5.65E-18
	Extracellular matrix organization	Col1a1	1.78E-21	0.47349159	0.861	0.663	2.81E-17
		Efemp2	6.41E-13	0.263687033	0.731	0.621	1.01E-08
		Olfml3	1.14E-08	0.305428125	0.568	0.446	1.81E-04
		Fbln2	1.36E-11	0.284824944	0.846	0.707	2.15E-07
		Col1a2	2.51E-14	0.424689073	0.97	0.927	3.97E-10
	Regulation of insulin-like growth factor receptor signaling pathway	Mfap4	3.86E-16	0.443274965	0.372	0.183	6.11E-12
		Igfbp4	1.49E-16	0.516081653	0.955	0.916	2.36E-12
	Cell cycle regulation	Igfbp6	6.04E-43	0.976333449	0.959	0.877	9.55E-39
		Btg2	3.34E-13	0.278539327	0.787	0.616	5.28E-09
		Junb	5.85E-12	0.332008912	0.97	0.913	9.25E-08
		Gadd45g	7.53E-17	0.412939139	0.909	0.771	1.19E-12
		Gadd45b	4.74E-25	0.536993459	0.905	0.741	7.50E-21
		Nupr1	3.47E-08	0.411140207	0.898	0.83	5.49E-04
		Timp2	3.86E-09	0.259385184	0.949	0.908	6.11E-05
		Pim3	7.58E-12	0.282052772	0.76	0.635	1.20E-07
		Ier3	2.23E-10	0.307427974	0.931	0.838	3.53E-06
		Malat1	2.33E-23	0.431964213	1	1	3.69E-19
	Metabolic processes	Ccnl1	4.72E-13	0.259061277	0.93	0.853	7.47E-09
		Enpp2	6.04E-17	0.300015314	0.408	0.209	9.55E-13
		Cst6	6.50E-07	0.482798098	0.812	0.705	0.010286298
		Gstm5	4.21E-22	0.301742331	0.91	0.813	6.65E-18
		Sepp1	3.15E-19	0.371592596	0.714	0.527	4.99E-15
		Gstm1	1.70E-09	0.259127411	0.713	0.63	2.70E-05
		Cyb5a	5.61E-13	0.251067594	0.967	0.936	8.87E-09
		Gfpt2	1.07E-11	0.32921936	0.512	0.379	1.70E-07
		Cyb5r3	5.04E-10	0.267086102	0.997	0.992	7.98E-06
		Cst3	1.37E-14	0.302153108	1	0.998	2.16E-10
		Ppp1r15a	2.68E-15	0.309183629	0.914	0.802	4.24E-11
		Prg4	2.24E-10	0.457267479	0.243	0.12	3.55E-06
		Bhlhe40	9.18E-08	0.297504532	0.396	0.293	0.00145179
		Tsc22d1	9.51E-17	0.364455422	0.98	0.944	1.50E-12
		Ndufa11	3.49E-22	0.25956286	0.994	0.994	5.53E-18
		Ndufb2	2.72E-21	0.250125243	0.968	0.938	4.30E-17
		Aebp1	2.06E-16	0.260905567	0.998	0.984	3.26E-12
		Aldh2	7.24E-19	0.287547986	0.943	0.844	1.15E-14
		Gsn	4.96E-30	0.589437299	0.977	0.917	7.84E-26
		Sqstm1	8.68E-15	0.268839802	0.992	0.97	1.37E-10
		Clu	4.11E-11	0.308101482	0.537	0.378	6.51E-07
		Dusp1	1.46E-20	0.541418254	0.799	0.571	2.31E-16
		Gchfr	1.94E-07	0.336855101	0.442	0.353	0.003060609
		Guca1a	5.21E-09	0.262744239	0.292	0.176	8.24E-05
		S100a1	1.03E-06	0.310586576	0.847	0.782	0.016310317
		Hexa	1.26E-11	0.265642631	0.89	0.838	1.99E-07
		Penk	1.00E-12	0.613707772	0.417	0.259	1.58E-08
		Ly6a	1.09E-13	0.343714295	0.92	0.771	1.73E-09

	Signal transduction	Il11ra1	4.61E-14	0.297407902	0.78	0.672	7.29E-10
		Cryab	2.94E-15	0.318919976	0.694	0.448	4.65E-11
		Baiap2	2.30E-12	0.294017397	0.733	0.622	3.63E-08
		Sparcl1	1.89E-26	0.494634512	0.748	0.482	3.00E-22
		Lsp1	3.08E-11	0.252913277	0.863	0.744	4.87E-07
		Rbp1	1.06E-09	0.326871956	0.967	0.944	1.68E-05
		Gem	2.23E-10	0.290843937	0.626	0.485	3.53E-06
		Fcgrt	2.20E-15	0.300714105	0.694	0.529	3.48E-11
		Ogn	2.53E-16	0.345316301	0.347	0.168	4.01E-12
	Cellular component organization or biogenesis	P4ha3	6.30E-18	0.410924489	0.415	0.232	9.97E-14
		S100a4	8.74E-20	0.650280615	0.857	0.7	1.38E-15
		Aqp5	3.86E-14	0.518416887	0.125	0.019	6.11E-10
		Emp1	1.62E-26	0.359337292	0.99	0.986	2.57E-22
		Rps28	8.15E-37	0.298752504	1	1	1.29E-32
		Nbl1	1.37E-18	0.504893064	0.83	0.677	2.17E-14
		Map1lc3a	2.37E-28	0.393788687	0.979	0.925	3.75E-24
		Gabarapl1	1.83E-30	0.35713619	0.918	0.697	2.90E-26
		Mgp	4.53E-46	0.983930935	0.755	0.382	7.17E-42
		Neat1	5.40E-11	0.276395876	0.825	0.704	8.54E-07
		Crabp2	7.40E-45	1.009357775	0.618	0.293	1.17E-40
		Ugdh	2.65E-13	0.302066653	0.909	0.856	4.19E-09
		Stmn2	9.15E-25	0.652221798	0.496	0.253	1.45E-20
	Unclear function	500015O10Ri	6.50E-15	0.377602073	0.183	0.051	1.03E-10
		410015M20R	1.42E-33	0.30918419	0.996	0.969	2.24E-29
		AI413582	2.23E-14	0.303536539	0.735	0.602	3.52E-10
		Gm8292	3.14E-19	0.270838801	0.854	0.708	4.96E-15
		C1s1	1.14E-11	0.323343163	0.223	0.095	1.80E-07
		Hrsp12	5.95E-21	0.33068432	0.732	0.552	9.41E-17
		Cpxm1	6.32E-13	0.426638432	0.45	0.303	9.99E-09
		Klhdc8a	1.75E-10	0.338100544	0.401	0.262	2.77E-06
		Ypel3	5.52E-19	0.330226488	0.819	0.652	8.74E-15
		Ptms	7.98E-23	0.275619264	0.939	0.816	1.26E-18
		Hcfc1r1	4.28E-15	0.258969802	0.967	0.903	6.76E-11
Downregulated genes	Antigen processing and presentation	Psmb9	3.85E-17	-0.371628517	0.442	0.604	6.09E-13
		H2-K1	5.54E-27	-0.410756897	0.996	0.994	8.76E-23
		H2-Q4	1.50E-24	-0.359493188	0.39	0.632	2.37E-20
		H2-Q7	5.44E-20	-0.367793053	0.661	0.797	8.60E-16
		B2m	9.13E-27	-0.351245393	1	1	1.44E-22
		H2-Q6	1.61E-28	-0.454657864	0.306	0.563	2.54E-24
		H2-D1	3.57E-16	-0.267977104	0.999	0.995	5.65E-12
		Alcam	1.44E-23	-0.336910745	0.704	0.841	2.28E-19
		Psmb8	8.39E-13	-0.314291379	0.784	0.807	1.33E-08
		Ctsl	7.78E-25	-0.515623938	1	1	1.23E-20
	Mitosis	Aurkb	7.95E-17	-0.300561863	0.155	0.323	1.26E-12
		Prc1	5.82E-11	-0.321277864	0.311	0.448	9.21E-07
		Racgap1	9.82E-13	-0.25239538	0.34	0.505	1.55E-08
		Birc5	8.42E-08	-0.348846092	0.478	0.559	0.001331781
		Cenpe	1.24E-15	-0.320652496	0.217	0.396	1.96E-11
		Cdc20	1.28E-08	-0.368813462	0.486	0.576	2.03E-04
		Tpx2	1.54E-14	-0.32479475	0.292	0.468	2.44E-10
		Cfl1	1.33E-29	-0.253239297	1	1	2.10E-25
		Tk1	3.82E-10	-0.291282427	0.31	0.456	6.04E-06
		Orc6	2.13E-10	-0.265387589	0.759	0.836	3.36E-06
		Aurka	1.63E-16	-0.366831069	0.219	0.398	2.58E-12
		Smc4	1.42E-07	-0.307091459	0.791	0.785	0.002252096
		Cenpa	1.09E-07	-0.363165637	0.602	0.647	0.001717158
		Cdca8	9.58E-15	-0.434787434	0.396	0.534	1.52E-10
		Anln	1.80E-24	-0.316938076	0.218	0.449	2.85E-20
		Spc25	5.81E-11	-0.305005718	0.276	0.417	9.18E-07
		Cenpf	3.38E-09	-0.257812022	0.209	0.329	5.35E-05
		Tuba1b	4.76E-10	-0.268702634	0.992	0.989	7.53E-06
		Tuba1a	4.21E-13	-0.319059813	0.991	0.992	6.66E-09
		Tubb6	9.81E-17	-0.418231594	0.951	0.966	1.55E-12
		Nusap1	1.19E-14	-0.299312434	0.139	0.292	1.89E-10
		Tubb5	2.15E-13	-0.300297808	0.993	0.992	3.40E-09
	Cell cycle	Tipin	7.09E-12	-0.283561412	0.831	0.869	1.12E-07
		Ccna2	5.01E-11	-0.315414139	0.343	0.493	7.92E-07
		Plk1	1.43E-12	-0.267858239	0.143	0.281	2.26E-08
		Ccnb2	9.98E-13	-0.289666447	0.321	0.498	1.58E-08
		Calm1	3.38E-20	-0.274750425	1	1	5.35E-16
		Cks1b	1.81E-11	-0.26710165	0.986	0.991	2.86E-07
		Cdkn1c	6.24E-13	-0.583440813	0.442	0.585	9.86E-09
		Cdk1	7.80E-10	-0.466637775	0.507	0.591	1.23E-05
		Anp32b	6.22E-21	-0.26657313	0.995	0.998	9.84E-17
	Cytoskeleton organization	Tpm1	4.45E-18	-0.303151692	0.999	1	7.03E-14
		Tmsb4x	1.45E-21	-0.470840763	1	1	2.29E-17
		Pfn1	6.71E-26	-0.286967937	1	1	1.06E-21
		Sema7a	7.80E-16	-0.386607268	0.473	0.616	1.23E-11
		Nefl	1.81E-25	-0.725365072	0.27	0.504	2.86E-21

		Nrp1	3.22E-19	-0.354906772	0.339	0.554	5.09E-15
		Tgfb1i1	9.18E-12	-0.37964223	0.56	0.668	1.45E-07
		Lhx8	4.28E-19	-0.280684641	0.366	0.593	6.77E-15
		Gap43	4.73E-41	-0.929956991	0.509	0.711	7.47E-37
		Wnt5a	3.38E-15	-0.267901576	0.428	0.612	5.35E-11
	Developmental processes	Nrep	2.05E-12	-0.374807595	0.164	0.307	3.24E-08
		Grem1	1.72E-14	-0.378360091	0.636	0.713	2.73E-10
		Il33	1.73E-08	-0.392965239	0.787	0.811	2.74E-04
		Igfbp3	2.96E-06	-0.437286203	0.398	0.479	0.046826161
		Xist	2.76E-18	-0.303674159	0.967	0.973	4.37E-14
		Wdr63	1.45E-26	-0.401722535	0.127	0.342	2.30E-22
		Hmga2	2.09E-18	-0.31826487	0.917	0.959	3.31E-14
		Upp1	9.96E-07	-0.322040396	0.756	0.75	0.015754453
		Kras	1.25E-41	-0.54784007	0.722	0.871	1.98E-37
		Rpl13	5.05E-47	-0.256497581	1	1	7.99E-43
		Gins2	7.46E-13	-0.256523802	0.509	0.661	1.18E-08
		Csgalnact1	2.51E-23	-0.43285333	0.438	0.637	3.98E-19
		Crip2	2.53E-07	-0.329525669	0.686	0.693	0.003994357
		Mageh1	1.88E-34	-0.413915164	0.187	0.473	2.98E-30
		Ggct	1.13E-11	-0.383833269	0.353	0.504	1.79E-07
	Cell differentiation	Crhbp	3.86E-13	-0.339152701	0.141	0.289	6.11E-09
		Tnfrsf11b	2.04E-13	-0.324356923	0.176	0.331	3.23E-09
		Ankrd1	1.22E-18	-0.63809921	0.191	0.379	1.93E-14
		Dkk2	5.65E-52	-0.857189377	0.894	0.956	8.93E-48
		Vdac3	6.09E-17	-0.279085214	0.971	0.969	9.63E-13
		Ctsl	7.78E-25	-0.515623938	1	1	1.23E-20
		Ybx1	2.36E-31	-0.301696019	0.999	1	3.73E-27
		Sprr1a	7.03E-08	-0.289659406	0.148	0.259	0.001112353
		Gsta4	7.59E-16	-0.269554339	0.099	0.246	1.20E-11
		Gsr	5.80E-16	-0.295092129	0.444	0.629	9.17E-12
		Cndp2	6.69E-23	-0.34512908	0.371	0.593	1.06E-18
		Ankrd11	4.03E-19	-0.302082436	0.7	0.839	6.38E-15
		Ywhaq	4.61E-23	-0.268282428	0.967	0.983	7.29E-19
		Psip1	3.55E-20	-0.301016113	0.831	0.905	5.61E-16
		Top2a	4.73E-12	-0.401203338	0.341	0.482	7.49E-08
		Hmgb2	2.31E-13	-0.510701849	0.822	0.836	3.66E-09
		Eif5	1.50E-25	-0.292528602	0.99	1	2.37E-21
		Lum	3.14E-08	-0.333126333	0.721	0.755	4.97E-04
		Cox5a	8.25E-28	-0.251341186	1	1	1.30E-23
	Metabolic processes	Nucks1	3.97E-15	-0.275567922	0.816	0.872	6.28E-11
		Ppia	3.94E-27	-0.262609919	0.994	0.995	6.24E-23
		Gnai2	1.11E-18	-0.31658649	0.942	0.938	1.76E-14
		Tnc	7.16E-15	-0.49874128	0.678	0.758	1.13E-10
		Acpp	1.53E-17	-0.646407432	0.578	0.66	2.43E-13
		Srsf3	6.42E-14	-0.259121549	0.97	0.969	1.02E-09
		Tfpi2	1.32E-08	-0.440723846	0.375	0.499	2.08E-04
		Rassf1	1.18E-09	-0.263498216	0.704	0.772	1.87E-05
		Spp1	7.39E-16	-0.462224337	0.997	1	1.17E-11
		Thbs4	7.36E-18	-0.2609139	0.225	0.44	1.16E-13
		Celf2	3.74E-18	-0.27912419	0.262	0.463	5.91E-14
		Acsbg1	1.18E-19	-0.262783931	0.108	0.285	1.86E-15
		Mmp10	3.30E-15	-0.539203353	0.303	0.465	5.23E-11
		Asf1b	1.26E-14	-0.30812851	0.251	0.424	2.00E-10
		Cotl1	5.83E-31	-0.788398555	0.382	0.598	9.22E-27
		Tpm4	2.02E-27	-0.420722621	1	1	3.19E-23
		Antxr2	5.70E-19	-0.26951013	0.337	0.552	9.01E-15
		Fblim1	3.11E-12	-0.269982737	0.49	0.626	4.91E-08
		Ckap2l	5.48E-19	-0.274809677	0.137	0.318	8.67E-15
		H1f0	5.19E-08	-0.261312927	0.64	0.708	8.21E-04
	Cellular component organization	Lgals7	1.82E-09	-0.635065092	0.597	0.668	2.88E-05
		Hmmr	7.07E-12	-0.283813391	0.233	0.382	1.12E-07
		Lyz2	2.58E-26	-0.280986619	0.102	0.317	4.07E-22
		Lpp	9.31E-15	-0.294426822	0.556	0.693	1.47E-10
		Ntm	1.09E-29	-0.314754605	0.066	0.265	1.73E-25
		Neto2	9.93E-08	-0.256264813	0.611	0.696	0.001571393
		Slc14a1	1.50E-36	-0.603256149	0.269	0.555	2.37E-32
		Psmd7	2.54E-24	-0.331615948	0.993	0.992	4.02E-20
		Rps4l	9.72E-16	-0.33691324	0.854	0.883	1.54E-11
		H2afx	7.70E-13	-0.35539123	0.608	0.746	1.22E-08
		930579C12Ri	1.51E-21	-0.389888406	0.117	0.303	2.39E-17
		10039M20Ril	2.02E-54	-0.730553362	0.141	0.48	3.20E-50
		Gm12603	3.88E-36	-0.520171572	0.149	0.42	6.13E-32
		Gm23935	1.31E-22	-0.582356182	0.941	0.966	2.07E-18
	Unclear function	Ctxn1	7.26E-12	-0.269597047	0.473	0.608	1.15E-07
		Tagln2	2.75E-18	-0.309825798	0.994	0.994	4.34E-14
		Trp53i11	5.20E-42	-0.992852206	0.079	0.339	8.23E-38
		Fam64a	7.67E-16	-0.340768284	0.221	0.396	1.21E-11
		Filip1l	4.38E-15	-0.344063628	0.354	0.535	6.92E-11

Supplemental Table 4. Enriched biological processes in sarcoma SP cells relative to non-SP cells

	Upregulated Gene Ontology Process	Raw P-value	FDR	Downregulated Gene Ontology Process	Raw P-value	FDR
KPCC-840	Negative regulation of developmental process (GO:0051093)	2.66E-05	0.0031	Cell cycle (GO:0007049)	1.55E-05	4.88E-02
	Skeletal muscle cell differentiation (GO:0035914)	0.000554	0.0334	Cell cycle process (GO:0022402)	2.66E-05	4.65E-02
KPCC-00	Negative regulation of developmental process (GO:0051093)	2.22E-05	2.95E-03	Cell cycle (GO:0007049)	4.86E-16	1.91E-12
	Negative regulation of cell differentiation (GO:0045596)	6.54E-04	4.61E-02	Cell cycle process (GO:0022402)	5.73E-15	1.81E-11
	Muscle organ development (GO:0007517)	5.59E-04	4.19E-02			
KPCC-844	Negative regulation of developmental process (GO:0051093)	3.66E-19	9.16E-17	Cell cycle (GO:0007049)	3.41E-14	2.83E-11
	Regulation of stem cell proliferation (GO:0072091)	2.20E-04	5.40E-03	Cell cycle process (GO:0022402)	1.29E-09	4.61E-07
	Mesenchyme development (GO:0060485)	1.16E-07	6.20E-06			
	Muscle organ development (GO:0007517)	4.71E-08	2.69E-06			

Supplemental Table 5. Enriched biological processes in SP-RPF cells relative to SP-YFP cells

	Gene Otology Processes	Fold Enrichment	Raw P-value	FDR
**Upregulated Processes**	Regulation of establishment of protein localization to telomere (GO:0070203)	70.76	8.47E-07	6.36E-04
	Positive regulation of cellular senescence (GO:2000774)	53.07	4.55E-05	9.43E-03
	Nucleobase-containing small molecule biosynthetic process (GO:0034404)	13.33	5.01E-05	9.88E-03
	Mitotic cell cycle phase transition (GO:0044772)	10.38	1.55E-06	8.14E-04
	Regulation of mRNA splicing, via spliceosome (GO:0048024)	9.18	2.66E-04	3.23E-02
	Ribosome biogenesis (GO:0042254)	6.85	2.77E-06	1.18E-03
**Downregulated Processes**	Peptidyl-lysine oxidation (GO:0018057)	100	1.68E-07	3.31E-05
	Collagen fibril organization (GO:0030199)	44.38	2.59E-18	8.17E-15
	Regulation of plasminogen activation (GO:0010755)	40.42	9.79E-05	6.54E-03
	Regulation of transforming growth factor beta production (GO:0071634)	20.21	8.18E-06	8.71E-04
	Regulation of cell migration involved in sprouting angiogenesis (GO:0090049)	20.73	7.30E-06	7.89E-04
	Regulation of insulin-like growth factor receptor signaling pathway (GO:0043567)	19.4	6.65E-04	3.04E-02
	Regulation of establishment of protein localization to telomere (GO:0070203)	70.76	8.47E-07	6.36E-04
	Positive regulation of cellular senescence (GO:2000774)	53.07	4.55E-05	9.43E-03

Supplemental Table 6. Enriched biological processes in sarcoma non-SP-RFP cells relative to non-SP-YFP cells

	Gene Ontology Processes	Fold Enrichment	Raw P-value	FDR
**Upregulated Processes**	Electron transport coupled proton transport (GO:0015990)	> 100	4.09E-07	1.17E-04
	Cellular response to interleukin-4 (GO:0071353)	25.87	3.06E-06	7.53E-04
	Ribosome assembly (GO:0042255)	25.44	3.45E-13	1.51E-10
	Positive regulation of signal transduction by p53 class mediator (GO:1901798)	17.84	1.15E-04	1.74E-02
	Negative regulation of RNA splicing (GO:0033119)	17.84	1.15E-04	1.73E-02
	Peptide metabolic process (GO:0006518)	12.56	3.59E-34	1.88E-30
	Positive regulation of cell cycle phase transition (GO:1901989)	7.25	2.40E-04	3.20E-02
	Peptidyl-lysine oxidation (GO:0018057)	47.97	9.97E-05	5.91E-03
	Negative regulation of plasminogen activation (GO:0010757)	39.98	1.48E-04	8.26E-03
	Collagen biosynthetic process (GO:0032964)	35.54	1.49E-05	1.18E-03
	Positive regulation of transforming growth factor beta production (GO:0071636)	19.04	1.47E-05	1.17E-03
**Downregulated Processes**	Collagen-activated signaling pathway (GO:0038065)	17.13	1.12E-03	4.22E-02
	Regulation of platelet-derived growth factor receptor signaling pathway (GO:0010640)	12.79	4.24E-04	1.97E-02
	Positive regulation of epithelial to mesenchymal transition (GO:0010718)	11.42	5.62E-06	5.07E-04
	Negative regulation of wound healing (GO:0061045)	10.28	5.68E-07	7.00E-05
	Positive regulation of cell migration involved in sprouting angiogenesis (GO:0090050)	15.23	2.35E-04	1.22E-02
	Electron transport coupled proton transport (GO:0015990)	> 100	4.09E-07	1.17E-04
	Cellular response to interleukin-4 (GO:0071353)	25.87	3.06E-06	7.53E-04
	Ribosome assembly (GO:0042255)	25.44	3.45E-13	1.51E-10
	Positive regulation of signal transduction by p53 class mediator (GO:1901798)	17.84	1.15E-04	1.74E-02
	Negative regulation of RNA splicing (GO:0033119)	17.84	1.15E-04	1.73E-02
	Peptide metabolic process (GO:0006518)	12.56	3.59E-34	1.88E-30

Supplementary Table 7. Differenetial Gene Expression Analysis of RFP expressing SP cells relative to YFP expressing SP cells

	Function Group	Gene	p value	average logFC	pct.1	pct.2	adusted p value
Upregulated Genes	Biosynthetic process	Tpi1	3.70E-62	0.887355127	0.996	0.987	1.77E-57
		Fabp5	5.26E-16	0.528604944	0.855	0.786	2.51E-11
		Idh2	9.10E-30	0.520913229	0.901	0.789	4.35E-25
		Dctpp1	1.30E-26	0.511419671	0.933	0.882	6.22E-22
		Pold2	5.16E-20	0.348316614	0.855	0.783	2.46E-15
		Tkt	1.11E-15	0.336447751	0.94	0.942	5.29E-11
	Cell adhesion	Msln	2.09E-17	0.303101774	0.286	0.038	1.00E-12
	Cell cycle	Ccnd2	6.82E-30	0.617162735	0.951	0.914	3.25E-25
		Birc5	2.60E-17	0.43149614	0.749	0.473	1.24E-12
		Cdca3	1.82E-31	0.657430982	0.717	0.358	8.70E-27
	Cellular component biogenesis	Mgp	2.94E-27	2.194319221	0.661	0.329	1.40E-22
		Cd9	2.87E-25	0.608304564	0.993	0.99	1.37E-20
		Emp1	4.61E-32	0.563236984	0.989	0.997	2.20E-27
		Tubb5	1.83E-18	0.534190863	0.989	0.997	8.74E-14
		Csrp2	1.94E-15	0.496541826	0.961	0.958	9.25E-11
	Chromosome organization	Myc	5.80E-38	1.453775216	0.947	0.92	2.77E-33
		Cdk1	1.85E-21	0.586972041	0.792	0.556	8.84E-17
		Nap1l1	3.84E-41	0.506691367	0.986	0.949	1.83E-36
		Dpy30	1.07E-25	0.495236529	0.887	0.783	5.12E-21
		Cenpa	1.47E-13	0.443480933	0.784	0.597	7.03E-09
		Hmgb1	2.03E-18	0.41405129	0.979	0.981	9.67E-14
		Cenpw	1.40E-25	0.405871544	0.784	0.505	6.68E-21
		Top2a	5.00E-17	0.356604405	0.678	0.371	2.39E-12
		Cdca8	7.27E-16	0.327986521	0.753	0.457	3.47E-11
		Chd4	4.70E-17	0.31276913	0.894	0.837	2.24E-12
		Ccna2	1.36E-16	0.312152036	0.696	0.358	6.47E-12
		Cdc20	5.81E-13	0.311357128	0.77	0.562	2.77E-08
		Smc2	3.24E-13	0.308459649	0.795	0.658	1.54546E-08
		Ran	3.68E-16	0.300941212	1	0.997	1.76E-11
	Cytoskeleton organization	Stmn2	4.43E-07	0.505781701	0.406	0.259	2.11E-02
		Tuba4a	2.02E-19	0.315405159	0.58	0.31	9.64E-15
	Immune function	Ptms	2.25E-23	0.408916035	0.94	0.911	1.07E-18
	Iron maturation	Bola2	2.37E-26	0.34953816	0.954	0.914	1.13E-21
	Lipid metabolism	Slc16a11	3.98E-42	0.807627993	0.569	0.073	1.90E-37
		Crabp1	3.83E-11	0.435269158	0.996	0.994	1.83E-06
		Crabp2	1.35E-13	0.37693554	0.495	0.259	6.45528E-09
	Macromolecules transport	Nup37	8.62E-16	0.316726621	0.703	0.575	4.11E-11
	Metabolic regulation	Dlk1	1.38E-60	2.310964894	0.866	0.38	6.59E-56
		Gapdh	6.13E-65	0.836492802	0.996	1	2.93E-60
		Hbegf	2.74E-24	0.611710195	0.968	0.802	1.31E-19
		Dynap	3.65E-07	0.576686768	0.604	0.518	1.74E-02
		Aqp1	8.14E-10	0.501534757	0.519	0.335	3.89E-05
		Aldh2	1.83E-34	0.486612541	0.933	0.901	8.74E-30
		Mlf2	2.46E-35	0.41424935	0.993	0.978	1.18E-30
		Klf10	7.34E-12	0.403181139	0.675	0.537	3.50E-07
		Ramp3	2.82E-14	0.403151566	0.622	0.438	1.34E-09
		Enpp2	1.71E-11	0.372574992	0.357	0.144	8.18E-07
		Arl6ip1	1.40E-08	0.35291939	0.859	0.888	6.68E-04
		Meox2	3.95E-30	0.339248504	0.466	0.064	1.88E-25
		Atp2b1	1.58E-16	0.332441883	0.965	0.949	7.53E-12
		Ndufa4	7.11E-09	0.30769672	0.975	0.994	3.39E-04
		Naca	2.36E-26	0.307500607	1	1	1.13E-21
	Mitochondria respiratory chain	Uqcr11	3.19E-26	0.480209692	0.982	0.987	1.52E-21
	Mitochondrial assembly	mt-Nd4	9.30E-48	0.6761033	1	0.994	4.44E-43
		mt-Nd2	2.09E-42	0.655977733	0.986	0.974	9.99E-38
		mt-Nd1	2.25E-29	0.529485822	1	1	1.08E-24
		Gabarapl1	2.37E-28	0.500915314	0.922	0.773	1.13E-23
		mt-Nd5	2.87E-29	0.495948712	0.975	0.933	1.37E-24
		Ndufa9	1.34E-26	0.435790758	0.841	0.757	6.37E-22
		Timm13	5.51E-17	0.386651266	0.975	0.99	2.63E-12
		Nptx1	6.19E-18	0.379225337	0.565	0.281	2.95E-13
	p53 pathway	Mif	1.72E-45	0.721394691	1	1	8.23E-41
		Npm1	8.80E-21	0.359279656	1	1	4.20E-16
		Cdkn2a	1.35E-23	0.339274091	1	0.997	6.43E-19
		Rps26	7.75E-50	0.687066605	1	1	3.70E-45
		Rps18	8.80E-64	0.635842239	1	1	4.20E-59
		Rpl22l1	8.17E-22	0.585280373	0.993	0.994	3.90E-17
		Rps12	4.87E-40	0.558316592	1	1	2.32E-35
		Eef2	1.57E-31	0.535258514	1	1	7.49E-27
		Rpl10a	8.67E-47	0.515845675	1	1	4.14E-42
		Rpl36	2.62E-50	0.507223505	1	1	1.25E-45
		Rpl21	2.23E-56	0.498816172	1	1	1.06E-51
		Rps4x	7.77E-47	0.483345772	1	1	3.71E-42
		Rps20	2.50E-35	0.469010331	1	1	1.19E-30
		Rpl41	8.97E-34	0.467113289	1	1	4.28E-29
		Mrpl51	1.48E-31	0.456117147	0.922	0.882	7.09E-27
		Eif3k	2.13E-39	0.431615479	1	1	1.02E-34
		Thop1	8.25E-27	0.411060224	0.788	0.623	3.94E-22
		Rps9	9.24E-43	0.408956633	1	1	4.41E-38

		Eif3f	4.55E-33	0.406748498	1	1	2.17E-28
	Peptide metabolism	Rps27a	1.61E-42	0.406539917	1	1	7.69E-38
		Rps24	4.98E-34	0.391403576	1	1	2.38E-29
		Rpl28	1.80E-30	0.389683751	1	1	8.59E-26
		Fau	4.29E-31	0.378240305	1	1	2.05E-26
		Rps13	4.85E-38	0.3667497	1	1	2.32E-33
		Rpl18	9.01E-36	0.363273278	1	1	4.30E-31
		Rps16	2.36E-30	0.350965614	1	1	1.12E-25
		Rps11	2.70E-38	0.347600162	1	1	1.29E-33
		Vars	2.42E-21	0.34658193	0.859	0.776	1.15E-16
		Rpl19	6.14E-32	0.334697268	1	1	2.93E-27
		Rpl26	8.06E-30	0.330341331	1	1	3.85E-25
		Rpl27a	2.47E-26	0.325693369	0.993	0.997	1.18E-21
		Rps23	8.86E-26	0.320612849	1	1	4.23E-21
		Rps29	4.78E-21	0.3187118	1	1	2.28E-16
		Eef1g	4.47E-24	0.317308314	1	1	2.13E-19
		Eif3i	6.85E-21	0.306731857	0.989	0.997	3.27E-16
		Rps8	4.73E-34	0.304894671	1	1	2.26E-29
	Phosphate metabolism	Ppa1	4.72E-13	0.368288704	0.926	0.939	2.25E-08
	Phosphate tranport	Slc25a3	2.25E-24	0.359187477	1	1	1.08E-19
	Protein folding	Fkbp4	1.00E-60	1.008399273	0.975	0.888	4.79449E-56
		Pfdn6	8.65E-23	0.336626292	0.954	0.942	4.13E-18
		Clu	4.72E-33	0.910841809	0.689	0.259	2.25E-28
		Phb2	2.83E-66	0.845700457	0.996	0.981	1.35E-61
		Tomm7	4.11E-32	0.423996269	0.982	0.99	1.96E-27
	Protein stablization	Cct2	1.42E-20	0.363385829	0.972	0.971	6.80E-16
		Hspd1	1.10E-13	0.335301412	0.989	0.981	5.26E-09
		Cct7	9.86E-14	0.314997246	0.986	0.981	4.71E-09
		Cryab	7.91E-07	0.309878032	0.583	0.438	3.78E-02
	Protein synthesis	Mrpl42	9.62E-16	0.315024401	0.926	0.946	4.59E-11
		mt-Co1	1.57E-36	0.578752905	0.986	0.981	7.49E-32
	Proton transmembrane transport	mt-Cytb	2.28E-38	0.575737384	1	1	1.09E-33
		Atp5d	6.76E-48	0.548777382	1	1	3.22E-43
		Atp6v1e1	7.49E-24	0.34741514	0.912	0.917	3.57E-19
		Krt18	1.52E-39	1.146974661	0.65	0.173	7.23E-35
		Krt8	7.95E-18	0.884753843	0.307	0.051	3.80E-13
		Eps8	2.75E-46	0.648956246	0.922	0.767	1.31E-41
	Cytokine response	Ybx3	1.43E-39	0.547999597	0.951	0.907	6.84E-35
		Pcolce2	2.38E-38	0.429242162	0.615	0.131	1.14E-33
		Shmt2	2.29E-18	0.399958435	0.845	0.792	1.09E-13
		Wnk1	8.43E-30	0.385043877	0.83	0.604	4.02E-25
		Ncl	1.53E-12	0.34116572	1	0.997	7.31E-08
		Rpl32	1.30E-73	0.83281986	1	1	6.19E-69
		Cdk4	4.35E-32	0.472582511	0.993	0.994	2.07E-27
	Response to IL-4	Rplp0	4.97E-28	0.409764863	1	1	2.37E-23
		Rps2	6.28E-39	0.385848338	1	1	3.00E-34
		Tuba1b	3.11E-09	0.371336984	0.986	0.987	1.49E-04
		Rps15	5.15E-50	0.553529288	1	1	2.46E-45
		Rpl3	2.59E-37	0.483315837	1	1	1.24E-32
		Rps5	5.18E-42	0.479179877	1	1	2.47E-37
		Rps28	5.30E-36	0.448004263	0.996	1	2.53E-31
		Rpl23a	1.03E-37	0.440024802	1	1	4.91E-33
	Ribosome assembly	Rpl6	1.01E-46	0.416420611	1	1	4.83E-42
		Rpl5	1.15E-35	0.384970508	1	1	5.50E-31
		Nop2	2.12E-21	0.333353445	0.746	0.55	1.01E-16
		Rps19	1.08E-21	0.329932801	1	1	5.14E-17
		C1qbp	4.51E-16	0.318593596	0.993	0.981	2.15E-11
		Rpl36a	6.66E-46	0.464816568	1	1	3.18E-41
		Rps10	4.00E-49	0.434178806	1	1	1.91E-44
		Strap	7.35E-45	0.555149942	0.972	0.923	3.51E-40
		Snrpg	5.16E-43	0.516579039	0.996	0.978	2.46E-38
		Hnrnpf	1.24E-35	0.506631638	0.968	0.907	5.93E-31
		Snrpf	5.37E-32	0.452173887	0.986	0.981	2.56E-27
	Rna processing	Lsm2	1.03E-25	0.442990941	0.954	0.927	4.92E-21
		Magohb	4.46E-23	0.407327592	0.816	0.703	2.13E-18
		Pabpc1	1.90E-27	0.39154108	1	1	9.07E-23
		Snrpd3	2.21E-19	0.354533438	0.975	0.965	1.06E-14
		Snrpd2	5.80E-27	0.320626653	0.993	1	2.77E-22
		Sf3b5	1.52E-17	0.315294484	0.905	0.946	7.23E-13
		Emg1	3.75E-54	0.700536993	0.975	0.939	1.79E-49
		Lyar	7.35E-35	0.539339432	0.982	0.952	3.51E-30
		Pa2g4	1.26E-14	0.428251762	0.919	0.904	6.03E-10
	rRNA processing	Wbp11	2.26E-29	0.422608785	0.813	0.578	1.08E-24
		Nhp2	3.88E-21	0.387143745	0.986	0.981	1.85E-16
		Ddx47	1.03E-26	0.361474987	0.781	0.594	4.93E-22
		Nop10	4.00E-13	0.33299562	0.996	0.997	1.91E-08
		Fbl	1.43E-15	0.301970031	0.954	0.958	6.82E-11
		Sparcl1	1.97E-23	1.074242205	0.71	0.473	9.40E-19
		Ptn	3.33E-35	0.848734138	0.707	0.268	1.59E-30
		Trbc2	3.31E-36	0.561139395	0.643	0.16	1.58E-31
		Penk	2.55E-20	0.560531247	0.463	0.144	1.22E-15
	Signal transduction						

	Signal transduction	Rgs17	6.16E-25	0.435046031	0.731	0.46	2.94E-20
		Brk1	6.98E-35	0.40962585	0.972	0.974	3.33E-30
		Tnfrsf12a	7.15E-11	0.358884942	0.979	0.971	3.41E-06
		Ociad2	1.52E-31	0.645430548	0.707	0.39	7.26E-27
	Unclear function	H2afj	1.66E-30	0.630170542	0.965	0.984	7.92E-26
		mt-Rnr1	1.90E-27	0.521649892	1	1	9.05E-23
		Gm3511	4.53E-33	0.493902576	0.989	0.997	2.16E-28
		Gltscr2	7.07E-26	0.414427871	0.954	0.952	3.37E-21
		mt-Rnr2	2.11E-10	0.403891816	1	1	1.01E-05
		Atp5b	1.97E-18	0.377583801	1	0.997	9.41E-14
		H2afv	2.99E-14	0.332613421	0.989	0.971	1.43E-09
		Lockd	1.91E-13	0.324584795	0.608	0.406	9.11E-09
		2410015M20Rik	1.87E-23	0.324429695	0.975	0.974	8.91E-19
		Gpi1	8.67E-20	0.322775451	0.979	0.958	4.14E-15
		Sssca1	1.26E-11	0.317755518	0.876	0.895	6.00E-07
		2810417H13Rik	9.22E-12	0.300352749	0.714	0.431	4.40E-07
		M6pr	3.76E-17	0.311073322	0.777	0.661	1.80E-12
		Gm10736	8.35E-22	0.341092636	0.972	0.971	3.99E-17
		Bri3bp	2.16E-22	0.306091236	0.88	0.754	1.03E-17
Downregulated Genes	Antigen processing	H2-D1	9.82E-35	-0.5515953	0.996	1	4.69E-30
		H2-K1	4.02E-42	-0.675446798	0.993	1	1.92E-37
	Apoptosis	Mageh1	2.61E-65	-0.556247088	0.145	0.866	1.25E-60
	Cell cycle	S100a11	3.40E-24	-0.460212582	0.993	1	1.62E-19
	Cell death	Tm2d2	1.55E-46	-0.540300801	0.696	0.962	7.37E-42
	Cell membrane protein	Gng11	6.14E-30	-0.662371434	0.905	0.99	2.93E-25
	Cell metabolism	Carkd	1.15E-24	-0.300738374	0.799	0.952	5.50E-20
		Gng5	2.72E-24	-0.302052013	0.982	1	1.30E-19
		Med10	9.75E-25	-0.30616139	0.837	0.974	4.65E-20
		a	1.68E-34	-0.307188894	0.138	0.636	8.02E-30
		Commd6	3.16E-29	-0.31048931	0.618	0.93	1.51E-24
		Spock3	2.29E-29	-0.314585857	0.099	0.527	1.09E-24
		Fnta	9.29E-26	-0.316647611	0.731	0.939	4.43E-21
		Celf2	6.99E-35	-0.324690391	0.283	0.78	3.34E-30
		Sec22b	1.80E-25	-0.324977844	0.848	0.968	8.58E-21
		Psmd7	2.70E-15	-0.325747982	0.94	0.994	1.29E-10
		Pla1a	2.23E-31	-0.328611185	0.049	0.476	1.06E-26
		Vcan	1.66E-26	-0.328731292	0.375	0.812	7.95E-22
		Gsr	2.23E-17	-0.328766669	0.544	0.834	1.06E-12
		Pld3	1.89E-16	-0.33156564	0.615	0.872	9.02E-12
		Casp12	7.44E-28	-0.339091772	0.265	0.703	3.55E-23
		Cmc2	8.13E-24	-0.34031722	0.693	0.93	3.88E-19
		Sin3b	2.61E-23	-0.343041589	0.862	0.974	1.25E-18
		Mthfsl	9.34E-30	-0.346755022	0.463	0.831	4.46E-25
		Rassf1	9.93E-17	-0.34689817	0.686	0.904	4.74E-12
		Ppib	1.65E-32	-0.350289202	1	1	7.86E-28
		Morf4l2	2.89E-24	-0.350539858	0.972	0.99	1.38E-19
		Serpinb6a	4.05E-19	-0.353213678	0.972	1	1.93E-14
		S100a1	2.53E-25	-0.357406216	0.693	0.949	1.21E-20
		Nnmt	5.50E-29	-0.359078649	0.272	0.738	2.62E-24
		Arl2bp	4.62E-28	-0.36025568	0.629	0.917	2.20E-23
		Lama4	9.45E-23	-0.361461702	0.244	0.655	4.51E-18
		Mmd	1.24E-18	-0.365440404	0.636	0.879	5.91E-14
		Ethe1	4.66E-30	-0.367041246	0.65	0.923	2.23E-25
		Emb	1.42E-21	-0.368821978	0.664	0.904	6.76E-17
		Ppic	1.11E-23	-0.377923043	0.986	1	5.29E-19
		Sec61b	3.72E-28	-0.381451358	0.996	1	1.78E-23
		Ly6e	4.51E-24	-0.390307587	1	1	2.15E-19
		Psmb9	4.47E-28	-0.404086807	0.484	0.866	2.14E-23
		Akr1b8	1.34E-18	-0.406559235	0.756	0.917	6.42E-14
		Pdlim1	9.71E-25	-0.416882634	0.693	0.927	4.63E-20
		Hspa1b	5.84E-14	-0.420319498	0.594	0.843	2.79E-09
		Mbnl2	3.62E-33	-0.423573745	0.753	0.984	1.73E-28
		Comt	1.91E-35	-0.423799468	0.671	0.974	9.13E-31
		Rexo2	5.37E-30	-0.432635901	0.961	0.997	2.56E-25
		Itm2b	1.56E-17	-0.432693856	0.993	1	7.45E-13
		Tcf25	2.21E-42	-0.458705413	0.876	0.981	1.06E-37
		Plac8	7.29E-12	-0.459928456	0.141	0.39	3.48E-07
		Tmsb4x	1.13E-18	-0.461445549	1	1	5.40E-14
		Vdac3	1.87E-32	-0.46784641	0.922	0.978	8.94E-28
		Cndp2	2.30E-44	-0.475469557	0.406	0.879	1.10E-39
		Ostc	1.52E-33	-0.480596606	0.954	0.997	7.27E-29
		Angptl4	2.90E-29	-0.490624364	0.35	0.799	1.39E-24
		Sat1	1.11E-08	-0.499583586	0.866	0.962	5.30E-04
		Atf5	7.24E-20	-0.505722423	0.852	0.987	3.46E-15
		Psmb8	3.97E-32	-0.533380749	0.664	0.987	1.90E-27
		Slc25a4	1.12E-47	-0.53346074	0.996	1	5.36E-43
		Ifi27	5.97E-35	-0.59422932	0.813	0.99	2.85E-30
		Crip1	7.56E-25	-0.628180366	0.834	0.978	3.61E-20
		Upp1	2.61E-34	-0.758028692	0.604	0.933	1.24E-29
		Cdkn1c	1.55E-41	-0.80677269	0.389	0.907	7.42E-37
		Rnase4	6.23E-43	-0.835339472	0.519	0.927	2.97E-38

		Dnajb1	3.35E-13	-0.898805617	0.919	0.965	1.60E-08
		Plat	2.93E-56	-0.943710169	0.686	0.994	1.40E-51
		Lgals7	1.90E-30	-0.946903614	0.569	0.869	9.05E-26
		Ass1	4.05E-53	-0.977412751	0.392	0.936	1.94E-48
		Cebpd	4.74E-41	-1.081110619	0.604	0.946	2.26E-36
		Tnc	4.52E-42	-1.235920373	0.636	0.955	2.16E-37
	Cell migration	Ada	8.83E-41	-0.303827621	0.152	0.69	4.22E-36
		Pfn2	1.39E-19	-0.306734325	0.463	0.783	6.62E-15
		Wdr63	1.07E-33	-0.358070537	0.155	0.636	5.13E-29
		Dnaja4	4.21E-13	-0.401740613	0.254	0.524	2.01E-08
		Tpm1	4.59E-22	-0.502277495	0.996	0.997	2.19E-17
		Calr	7.98E-33	-0.548677753	1	1	3.81E-28
		Igfbp3	2.49E-25	-0.565025447	0.35	0.776	1.19E-20
		Il33	7.43E-24	-0.747165755	0.717	0.895	3.55E-19
		Thbs4	4.36E-54	-0.891051432	0.145	0.818	2.08E-49
		Thy1	9.08E-26	-0.940721434	0.887	0.965	4.33E-21
		Col3a1	8.00E-17	-1.04636346	0.922	0.974	3.82E-12
		Dcn	2.85E-26	-1.199874841	0.887	0.971	1.36E-21
	Cell surface protein	Steap1	2.09E-26	-0.316028941	0.473	0.879	9.98E-22
		H2-Q6	3.82E-29	-0.345331489	0.413	0.866	1.83E-24
		Tm4sf1	3.56E-20	-0.409323721	0.986	0.997	1.70E-15
	Cell-cell adhesion	Lpp	4.12E-31	-0.396317988	0.565	0.914	1.96E-26
	Cell-cell Interaction	Thbs2	4.30E-18	-0.590019997	0.551	0.837	2.05E-13
	Cellular component organization/ biogenesis	Itgb5	3.05E-15	-0.300122388	0.346	0.649	1.46E-10
		Chrna1	1.13E-08	-0.30023774	0.636	0.802	5.40E-04
		Dynlt3	2.84E-20	-0.305476546	0.784	0.949	1.36E-15
		Ltbp2	1.16E-21	-0.309995297	0.272	0.681	5.55E-17
		Col15a1	8.21E-16	-0.315811369	0.042	0.297	3.92E-11
		Gfod2	8.18E-08	-0.316144293	0.258	0.492	0.003906242
		Tex264	8.93E-25	-0.317413487	0.71	0.962	4.26E-20
		Pdlim5	8.84E-21	-0.325550038	0.555	0.879	4.22E-16
		Vmp1	4.17E-21	-0.327950923	0.919	0.987	1.99E-16
		Ciapin1	1.04E-21	-0.353895151	0.767	0.949	4.95E-17
		Ggct	9.18E-17	-0.357633089	0.417	0.735	4.38E-12
		Sept7	5.19E-23	-0.377750698	0.975	1	2.48E-18
		Tnfrsf11b	6.34E-33	-0.380381969	0.127	0.623	3.02E-28
		Tuba1a	2.14E-09	-0.385587594	0.975	0.997	1.02E-04
		Cnn3	2.40E-28	-0.387078994	0.894	0.981	1.15E-23
		Lamb1	1.39E-25	-0.398412037	0.816	0.978	6.63E-21
		Trappc4	1.87E-27	-0.406252652	0.905	0.978	8.95E-23
		Palld	5.60E-35	-0.409338462	0.498	0.895	2.67E-30
		Nid1	1.21E-30	-0.429915468	0.484	0.914	5.78E-26
		Atp6v0d1	1.83E-38	-0.434921924	0.689	0.942	8.72E-34
		Col5a3	1.61E-38	-0.449103437	0.155	0.706	7.68E-34
		Surf4	2.15E-35	-0.451471787	0.89	0.981	1.03E-30
		Pdlim2	2.19E-26	-0.475131338	0.731	0.965	1.04E-21
		Csrp1	5.11E-27	-0.475227849	0.742	0.949	2.44E-22
		Rhoc	4.78E-38	-0.493584543	0.975	1	2.28E-33
		Anxa6	2.02E-40	-0.513638575	0.615	0.942	9.63E-36
		Tmed3	4.67E-38	-0.562726845	0.954	0.987	2.23E-33
		Col4a1	4.96E-08	-0.681971992	0.604	0.725	0.002366068
		Csgalnact1	9.82E-62	-0.69336247	0.42	0.942	4.69E-57
		Col4a2	1.20E-08	-0.74227226	0.64	0.741	5.72E-04
		Tpm4	5.69E-52	-0.761964393	0.989	1	2.71E-47
		Cotl1	2.62E-67	-1.013606074	0.325	0.927	1.25E-62
		Hist1h2bc	4.86E-48	-1.081056864	0.622	0.965	2.32E-43
		Gap43	4.18E-59	-1.087290155	0.484	0.946	2.00E-54
	Collagen metabolic process	Ctsb	2.59E-25	-0.402622549	0.996	1	1.24E-20
		Col5a1	1.17E-28	-0.407921831	0.417	0.863	5.56E-24
		Col1a2	7.69E-10	-0.425070417	0.908	0.981	3.67E-05
		Col6a2	8.60E-22	-0.449644656	0.452	0.821	4.11E-17
		Col1a1	1.08E-07	-0.483064805	0.587	0.84	0.005159085
		Serpinh1	1.47E-33	-0.521114309	0.996	1	7.01E-29
		Rcn3	1.07E-27	-0.631540004	0.887	0.978	5.10E-23
		Mmp2	9.76E-24	-0.737600362	0.802	0.981	4.66E-19
		Ctsl	1.25E-45	-0.925176754	1	1	5.94E-41
		Mmp3	1.96E-26	-1.762605536	0.406	0.776	9.37E-22
		Mmp10	8.61E-55	-1.877828348	0.201	0.796	4.11E-50
	Developmental process	Tnfaip2	1.77E-21	-0.311328307	0.597	0.911	8.43E-17
		Mxra8	3.66E-16	-0.313214361	0.908	0.987	1.75E-11
		Fndc3b	2.26E-25	-0.367399264	0.572	0.898	1.08E-20
		Plet1	7.71E-14	-0.38924227	0.194	0.489	3.68E-09
		Tagln2	9.69E-20	-0.408378876	0.961	1	4.63E-15
		Cd151	8.29E-33	-0.420533086	0.544	0.917	3.96E-28
		Tagln	7.29E-10	-0.423951552	0.286	0.585	3.48E-05
		Hsbp1	7.62E-55	-0.536210884	0.986	0.997	3.64E-50
		Sprr1a	2.62E-15	-0.641419277	0.148	0.441	1.25E-10
	Epithelial to Mesenchymal transition	Tgfbr2	4.23E-19	-0.331148368	0.562	0.866	2.02E-14
		Mdk	1.02E-16	-0.435876617	0.686	0.901	4.85E-12
		Pdpn	9.74E-24	-0.519534277	0.933	0.984	4.65E-19
		Tgfb1i1	1.45E-33	-0.526200812	0.473	0.901	6.92E-29

	Glipr2	7.14E-42	-0.533679323	0.562	0.933	3.41E-37
	Grem1	1.59E-47	-0.862831865	0.505	0.917	7.57E-43
Extracellular matrix organization	Timp1	2.29E-19	-0.560935786	0.982	1	1.09E-14
	Tnfaip6	6.00E-43	-0.768043642	0.399	0.885	2.86E-38
	Sparc	6.11E-40	-0.941314564	0.982	1	2.92E-35
Collagen fibril organization	Col6a1	8.21E-12	-0.355804254	0.7	0.898	3.92E-07
Collagen fibril organization	Ext1	1.13E-25	-0.369204318	0.428	0.84	5.37E-21
	Col5a2	5.16E-30	-0.70825983	0.519	0.888	2.46E-25
Golgi transport	Copb2	2.51E-16	-0.302633857	0.724	0.917	1.20E-11
	Trappc2l	1.49E-31	-0.379693297	0.845	0.984	7.09E-27
	Copz2	1.25E-32	-0.386020547	0.537	0.942	5.98E-28
	Lamp1	5.10E-35	-0.533485592	0.926	0.994	2.44E-30
	Cope	7.10E-44	-0.561536495	0.954	0.994	3.39E-39
	Kdelr3	3.77E-52	-0.660237649	0.495	0.946	1.80E-47
Ion binding	Calm1	6.80E-15	-0.301819574	1	1	3.25E-10
	S100a16	3.67E-12	-0.337207782	0.661	0.895	1.75E-07
	Fth1	8.15E-21	-0.522329697	1	1	3.89E-16
	Crip2	7.62E-43	-0.769904727	0.449	0.936	3.64E-38
Metal binding protein	Mt2	9.86E-43	-1.491888277	0.972	1	4.71E-38
Negative plasminogen activation	Serpine1	3.10E-12	-0.361348001	0.311	0.597	1.48E-07
	Thbs1	5.62E-31	-0.96096525	0.473	0.872	2.68E-26
	Serpine2	6.17E-26	-0.995479778	0.311	0.725	2.94E-21
Organelle acidification	Atp6v1b2	5.33E-27	-0.327441301	0.445	0.859	2.54E-22
Osteoclast formation	Ostf1	4.85E-18	-0.320795483	0.954	0.984	2.32E-13
Oxidative stress response	Sod3	8.05E-42	-0.986328322	0.102	0.645	3.84E-37
p53 pathway	Shisa5	3.27E-20	-0.318139314	0.926	1	1.56E-15
	Tbrg1	1.25E-39	-0.493936245	0.915	0.99	5.95E-35
	Trp53i11	3.39E-39	-0.949693027	0.12	0.633	1.62E-34
Pdfgr Signaling	Ift20	1.01E-27	-0.401492004	0.852	0.99	4.82E-23
	Glrx	2.19E-29	-0.478895256	0.611	0.911	1.04E-24
	Apod	8.76E-11	-0.749304414	0.258	0.546	4.18E-06
Peptidyl-lysine oxidation	Lox	5.93E-16	-0.403459298	0.367	0.703	2.83E-11
	Loxl2	9.50E-43	-0.800847748	0.502	0.93	4.53E-38
	Loxl1	4.26E-60	-0.897069276	0.558	0.974	2.03E-55
Phosphoprotein	Tceal8	3.18E-23	-0.376273876	0.915	0.994	1.52E-18
Protein binding	Cald1	1.53E-17	-0.334875853	0.996	1	7.32E-13
	Ppp1r2	7.84E-22	-0.361654018	0.82	0.958	3.74E-17
	Wnt5a	7.07E-30	-0.430439575	0.516	0.866	3.38E-25
	Ngf	2.18E-19	-0.430505501	0.466	0.802	1.04E-14
	Bdnf	1.83E-29	-0.504496811	0.587	0.917	8.74E-25
	Map1lc3b	3.26E-41	-0.551305873	0.993	1	1.55E-36
	B2m	8.66E-42	-0.591335948	1	1	4.13E-37
	App	3.93E-39	-0.643799513	0.756	0.978	1.87E-34
Protein-protein interaction	Wbp5	6.05E-25	-0.373638965	0.979	1	2.89E-20
Ras signaling	Apbb1ip	2.76E-22	-0.301453048	0.583	0.891	1.32E-17
Regulation of cell communication	Fgfr1	4.79E-21	-0.305265823	0.929	0.99	2.29E-16
	Nupr1	1.49E-15	-0.305500905	0.728	0.981	7.10E-11
	P4hb	2.45E-21	-0.307016572	0.958	0.997	1.17E-16
	Ctsh	7.97E-27	-0.31296703	0.18	0.613	3.80E-22
	Fkbp8	1.49E-26	-0.315736468	0.961	0.994	7.13E-22
	Gabarap	7.01E-18	-0.32133139	0.993	1	3.34E-13
	Hmgn3	2.96E-16	-0.322311967	0.912	0.971	1.41E-11
	Fez1	9.96E-13	-0.340718545	0.806	0.917	4.75E-08
	Rbms3	1.51E-30	-0.344693451	0.417	0.843	7.23E-26
	Pdgfrb	1.36E-30	-0.346587736	0.173	0.649	6.47E-26
	Crhbp	2.59E-22	-0.350987234	0.163	0.543	1.23E-17
	Igfbp7	2.47E-22	-0.351386311	0.145	0.537	1.18E-17
	Herpud1	1.63E-23	-0.352399872	0.555	0.904	7.80E-19
	Casp4	9.20E-37	-0.360500803	0.159	0.674	4.39E-32
	Itm2c	4.49E-22	-0.363199137	0.954	0.994	2.14E-17
	Cd81	7.23E-25	-0.369977183	0.986	1	3.45E-20
	Adam9	1.20E-31	-0.372744064	0.502	0.904	5.73E-27
	Ptgis	3.85E-26	-0.376300927	0.541	0.869	1.84E-21
	Dap	1.49E-23	-0.387225508	0.975	0.997	7.13E-19
	Uba5	2.21E-31	-0.390270749	0.587	0.904	1.05E-26
	Higd1a	1.48E-24	-0.39846225	0.859	0.965	7.08E-20
	Rbpms	1.02E-54	-0.412235377	0.226	0.853	4.85E-50
	Adm	8.00E-16	-0.413055252	0.208	0.524	3.82E-11
	Cyba	1.10E-34	-0.43165441	0.993	1	5.24E-30
	Ninj1	3.54E-26	-0.434883036	0.615	0.92	1.69E-21
	Hspb1	3.13E-12	-0.454089415	0.735	0.942	1.49E-07
	Serp1	2.06E-34	-0.470957564	0.986	0.994	9.83E-30
	Htra1	2.76E-35	-0.485934575	0.7	0.997	1.32E-30
	Fstl1	4.86E-09	-0.489761338	0.965	0.997	2.32E-04
	Nsg1	7.20E-30	-0.494830828	0.519	0.904	3.44E-25
	Nrep	6.39E-34	-0.506471569	0.102	0.581	3.05E-29
	S100a13	2.24E-33	-0.522497215	0.837	0.997	1.07E-28
	Sema7a	1.35E-33	-0.524403971	0.452	0.885	6.43E-29
	Rgs16	4.52E-15	-0.524610735	0.753	0.92	2.16E-10
	Cmtm3	2.54E-42	-0.532103199	0.841	0.984	1.21E-37
	Stra6	5.78E-27	-0.559264285	0.201	0.613	2.76E-22

		Litaf	5.46E-40	-0.580747506	0.901	0.99	2.60E-35
		Gnai2	5.55E-53	-0.590678002	0.887	0.994	2.65E-48
		Ecm1	3.86E-26	-0.591964204	0.894	0.994	1.84E-21
		Il1rl1	1.90E-16	-0.655387122	0.297	0.604	9.06E-12
		Kras	2.29E-50	-0.690730752	0.763	0.987	1.09E-45
		Fbn1	1.18E-33	-0.76856588	0.569	0.927	5.64E-29
		Ankrd1	1.24E-35	-0.793271956	0.159	0.661	5.92E-31
		Dkk2	4.89E-34	-0.845912456	0.862	0.971	2.33E-29
		Acpp	5.65E-74	-1.266999749	0.36	0.968	2.70E-69
		Ccl8	1.91E-13	-1.38508022	0.092	0.332	9.11E-09
		Spp1	1.60E-52	-1.577491034	1	1	7.65E-48
	Regulation of cell migration involvedlin sprouting angiogenesis	Rhoj	2.13E-54	-0.460447862	0.205	0.856	1.02E-49
		Nrp1	7.04E-57	-0.60785177	0.276	0.911	3.36E-52
		Hmox1	1.03E-13	-0.615749352	0.562	0.859	4.94E-09
	RNA Splicing	Ccdc12	1.13E-26	-0.382601183	0.82	0.978	5.41E-22
	Secretory pathway	Rrbp1	4.42E-40	-0.52230489	0.993	1	2.11E-35
	Transforming growth factor beta production	Cd34	8.63E-20	-0.385468741	0.88	0.962	4.12E-15
		Cd200	4.71E-30	-0.442569597	0.36	0.815	2.25E-25
		Ptgs2	4.63E-21	-0.7440148	0.544	0.869	2.21E-16
		Lum	7.60E-41	-1.022382813	0.601	0.923	3.63E-36
	Urea transport	Slc14a1	1.82E-12	-0.321910056	0.452	0.741	8.70E-08
	Wound healing	Tfpi	2.58E-49	-0.302000939	0.11	0.748	1.23E-44
		Prdx2	1.21E-23	-0.325048095	0.996	1	5.76E-19
		Anxa2	7.33E-16	-0.339721984	0.989	1	3.50E-11
		Pros1	8.27E-32	-0.354734345	0.428	0.866	3.95E-27
		Gja1	1.56E-25	-0.409570426	0.837	0.978	7.44E-21
		Serping1	6.08E-08	-0.427985637	0.346	0.562	0.0029011
		Anxa5	3.44E-28	-0.462529832	1	1	1.64E-23
		Itgb1	8.07E-47	-0.609047076	0.94	0.997	3.85E-42
		Tfpi2	8.86E-46	-0.776689112	0.286	0.843	4.23E-41
		Ccl2	6.02E-20	-0.995921585	0.83	0.971	2.87E-15
	Unclear function	Plekho2	2.54E-27	-0.31292971	0.413	0.824	1.21E-22
		Igsf10	1.78E-15	-0.318183899	0.431	0.754	8.48E-11
		Sept15	2.29E-30	-0.327929604	1	1	1.09E-25
		Cdk2ap2	1.52E-18	-0.340455386	0.852	0.962	7.27E-14
		Ctxn1	4.66E-26	-0.349333141	0.53	0.853	2.23E-21
		Ccdc124	1.71E-25	-0.353947436	0.827	0.971	8.17E-21
		D8Ertd738e	1.03E-33	-0.357403141	1	1	4.90E-29
		Hist1h1c	5.05E-14	-0.35802769	0.392	0.684	2.41E-09
		Srxn1	1.16E-16	-0.371366031	0.604	0.859	5.56E-12
		Gm6634	7.26E-29	-0.374835725	0.357	0.815	3.46E-24
		Leprotl1	3.00E-31	-0.375568086	0.799	0.981	1.43E-26
		Fam96b	1.45E-32	-0.38763667	0.883	0.971	6.90E-28
		2810428I15Rik	1.58E-33	-0.407516227	0.908	0.987	7.53E-29
		Filip1l	2.85E-23	-0.409516019	0.417	0.764	1.36E-18
		4930579C12Rik	3.27E-30	-0.411903628	0.113	0.55	1.56E-25
		Saraf	9.10E-32	-0.441198755	0.876	0.981	4.34E-27
		Vimp	4.85E-30	-0.447605062	0.859	0.994	2.32E-25
		C1qtnf6	7.74E-42	-0.519737472	0.223	0.786	3.69E-37
		Cyr61	2.47E-11	-0.53089678	0.848	0.965	1.18E-06
		Lhfp	2.25E-41	-0.584602359	0.703	0.984	1.08E-36
		Npdc1	4.20E-55	-0.598864776	0.562	0.981	2.00E-50
		Selm	1.41E-46	-0.698678927	0.551	0.965	6.75E-42
		Rps4l	2.64E-50	-0.720371637	0.728	0.978	1.26E-45
		3110039M20Rik.1	1.22E-75	-0.780983267	0.117	0.904	5.80E-71

Supplementary Table 8. Differenetial Gene Expression Analysis of RFP expressing non-SP cells relative to YFP expressing non-SP cells

	Function Group	Gene	p value	average logFC	pct.1	pct.2	adusted p value
Upregulated Genes	Acetylation inhibitor	Set	9.31E-25	0.490056741	0.97	0.893	4.45E-20
	Actin filament organization	Fam49b	4.68E-27	0.842016887	0.773	0.488	2.23E-22
		Tmsb4x	3.46E-15	0.44008872	0.999	0.997	1.65E-10
		Pfn1	1.99E-27	0.380536325	1	1	9.51E-23
	Cell adhesion	Alcam	1.74E-24	0.413532635	0.625	0.308	8.32E-20
	Cell cycle	Ccnd1	1.18E-17	0.61867465	0.801	0.595	5.62E-13
		Cks1b	1.02E-23	0.564486885	0.927	0.799	4.88E-19
		Eps8	1.09E-29	0.474467947	0.798	0.526	5.21E-25
		Ran	8.53E-24	0.417090749	0.99	0.972	4.07E-19
		Cdc20	2.32E-23	0.411904715	0.553	0.204	1.11E-18
		Cenpa	2.61E-17	0.356554368	0.594	0.294	1.24E-12
		S100a10	1.03E-11	0.349756479	0.996	0.99	4.90E-07
		Birc5	3.09E-16	0.348899713	0.508	0.225	1.48E-11
		Calm1	2.16E-19	0.345324587	1	1	1.03E-14
		Nudc	2.13E-22	0.338575752	0.948	0.855	1.02E-17
		Phb2	3.23E-22	0.320546115	0.987	0.958	1.54E-17
		Cdk4	5.56E-25	0.318180929	0.992	0.969	2.65E-20
		Apex1	4.84E-21	0.308416439	0.887	0.713	2.31E-16
		Cdca8	4.56E-17	0.303097783	0.485	0.19	2.18E-12
	Cell growth	Gap43	1.93E-24	0.783101851	0.478	0.166	9.23E-20
		Areg	3.29E-08	0.561609913	0.56	0.401	0.001571185
	Cell metabolism	Ctsl	2.33E-28	1.410467851	1	1	1.11E-23
		Odc1	6.12E-24	0.656439798	0.627	0.332	2.92E-19
		Dtx2	1.37E-22	0.627119556	0.556	0.26	6.54E-18
		Cdkn1c	6.36E-12	0.583862382	0.512	0.291	3.04E-07
		Ranbp1	1.44E-34	0.574377345	0.98	0.955	6.87E-30
		Hmgn1	1.13E-33	0.475997961	0.996	0.976	5.40E-29
		Hmgb2	2.41E-18	0.43495853	0.697	0.405	1.15E-13
		Fabp5	3.91E-28	0.434943962	0.744	0.415	1.87E-23
		Eno3	2.48E-08	0.432456467	0.461	0.27	0.001184561
		Prkg2	8.03E-32	0.426296871	0.656	0.246	3.83E-27
		Phgdh	4.57E-16	0.424743059	0.774	0.54	2.18E-11
		Pla2g7	1.05E-14	0.414111493	0.576	0.308	5.00E-10
		Eif3h	5.12E-08	0.395063239	0.999	1	0.002445124
		Mrpl13	8.57E-17	0.389519944	0.93	0.813	4.09E-12
		Uchl3	1.45E-23	0.382466227	0.86	0.664	6.94E-19
		Cnbp	7.94E-27	0.362667108	0.996	0.993	3.79E-22
		Nme1	3.60E-24	0.362653306	0.943	0.869	1.72E-19
		Eif4ebp1	3.68E-22	0.362345458	0.976	0.927	1.75E-17
		Fkbp4	1.58E-17	0.361980511	0.928	0.862	7.53E-13
		Ppid	3.63E-18	0.358938939	0.857	0.699	1.73E-13
		Txn1	2.61E-20	0.351132561	1	1	1.25E-15
		Cycs	1.82E-20	0.349610816	0.897	0.727	8.69E-16
		Mrps6	7.78E-18	0.348644645	0.91	0.768	3.71E-13
		Psma7	7.97E-24	0.341463464	0.992	0.993	3.81E-19
		Prmt1	9.58E-18	0.339886478	0.967	0.903	4.57E-13
		Orc6	3.94E-23	0.339738446	0.769	0.471	1.88E-18
		Dnajc2	9.77E-23	0.338218307	0.842	0.619	4.67E-18
		Polr2f	1.12E-21	0.335873024	0.975	0.934	5.34E-17
		Hmgn5	1.09E-14	0.331210085	0.739	0.529	5.20E-10
		Pxn	1.02E-19	0.329079809	0.692	0.436	4.88E-15
		Atp5f1	1.28E-19	0.324258025	0.998	0.986	6.11E-15
		Cpe	1.64E-07	0.308362215	0.754	0.557	0.007843043
		Polr2c	3.07E-24	0.307552708	0.941	0.803	1.47E-19
		Ak2	1.48E-17	0.307172802	0.926	0.82	7.05E-13
		Mrpl12	3.38E-19	0.305699209	0.93	0.858	1.61E-14
		Galnt1	1.76E-13	0.302803514	0.636	0.408	8.40E-09
		Sec11c	9.31E-12	0.302718453	0.792	0.64	4.45E-07
	Cell response to peptide hormone	Crhbp	2.76E-19	0.638968945	0.317	0.059	1.32E-14
	Cell-cell interaction	Lgals7	2.55E-16	0.963653361	0.51	0.277	1.22E-11
	Cytoskeleton organization	Krt18	1.11E-33	1.120925537	0.553	0.156	5.30E-29
		Nefl	2.72E-21	0.755833608	0.426	0.128	1.30E-16
		Krt8	1.35E-11	0.706310393	0.252	0.069	6.46E-07
		Tubb6	7.67E-10	0.409586657	0.946	0.91	3.66E-05
		Tuba1b	6.35E-11	0.361268423	0.98	0.955	3.03E-06
		Hn1	2.20E-20	0.341989551	0.915	0.827	1.05E-15
		Marcksl1	3.11E-17	0.320180947	0.941	0.848	1.48E-12
		Nes	7.16E-27	0.312746323	0.574	0.18	3.42E-22
	Developmental process	Twist2	2.42E-09	0.513141307	0.584	0.453	1.16E-04

	Developmental process	Crip2	9.81E-08	0.329924605	0.562	0.363	0.004681248
	DNA binding	Hmg20a	1.07E-17	0.439877097	0.421	0.156	5.13E-13
		H2afz	2.13E-22	0.418386222	0.867	0.664	1.01E-17
		Hmga1-rs1	1.02E-23	0.340351976	0.619	0.318	4.89E-19
	Enhancesome	Hmga2	1.54E-14	0.440553966	0.817	0.657	7.34E-10
	Ion transport	Kcnn4	1.41E-29	0.384651613	0.486	0.107	6.71E-25
	Nucleobase biosynthesis	Upp1	1.04E-38	0.755611546	0.682	0.273	4.99E-34
		Aprt	1.26E-20	0.389520524	0.993	0.99	6.01E-16
		Mtap	5.62E-22	0.3358484	0.829	0.564	2.68E-17
		Dctpp1	1.73E-17	0.315603921	0.796	0.557	8.27E-13
		Hint1	4.38E-22	0.306094266	0.998	1	2.09E-17
	Osmotic regulation	Gal	8.84E-12	1.569670638	0.437	0.208	4.22E-07
	Oxidation	Chrna1	3.28E-27	0.538306556	0.692	0.318	1.57E-22
		Cisd1	4.41E-19	0.308968304	0.867	0.73	2.11E-14
	Phosphoprotein	Anp32e	6.82E-24	0.415102461	0.808	0.574	3.26E-19
	Regulation of cellular senescence	Kras	5.39E-41	1.048500918	0.806	0.536	2.57E-36
		Cdkn2a	5.83E-23	0.548947788	0.942	0.803	2.78E-18
		Lsm6	1.16E-18	0.336335294	0.882	0.744	5.54E-14
		Eef1e1	2.05E-19	0.307706812	0.821	0.612	9.78E-15
	Regultion of translation	Ncl	7.27E-23	0.460701475	1	0.997	3.47E-18
		Eif5a	2.59E-22	0.366258525	1	1	1.24E-17
	RNA binding	Serbp1	3.14E-29	0.395661274	0.995	0.962	1.50E-24
	RNA processing	C1qbp	2.53E-32	0.432520736	0.979	0.941	1.21E-27
		Nhp2	1.97E-22	0.415305816	0.962	0.907	9.42E-18
		Lyar	1.83E-18	0.388998772	0.882	0.74	8.73E-14
		Npm1	1.77E-25	0.387129265	1	1	8.46E-21
		Rsl1d1	2.70E-27	0.374911373	0.929	0.782	1.29E-22
		Pa2g4	2.23E-17	0.355855329	0.915	0.83	1.07E-12
		Rbm8a	8.19E-18	0.353844898	0.975	0.945	3.91E-13
		Srsf3	2.75E-16	0.352008906	0.95	0.886	1.31E-11
		Ddx39	2.92E-18	0.328830018	0.789	0.578	1.40E-13
		Snrpe	1.86E-25	0.324067916	0.992	0.983	8.86E-21
		Hnrnpab	9.01E-20	0.320354554	0.978	0.938	4.30E-15
		Psip1	3.91E-22	0.312280906	0.895	0.727	1.86E-17
		Gar1	5.41E-16	0.30707939	0.85	0.692	2.58E-11
		Lsm2	3.59E-18	0.303587264	0.818	0.606	1.72E-13
	Signal transduction	Mpp6	2.21E-33	0.395117694	0.678	0.298	1.06E-28
	Telomere localization	Cct3	1.24E-25	0.427658251	0.966	0.896	5.90E-21
		Cct8	3.83E-27	0.414303293	0.982	0.931	1.83E-22
		Cct6a	1.07E-17	0.335735791	0.958	0.91	5.11E-13
		Cct7	2.90E-18	0.303402972	0.989	0.962	1.38E-13
	Urea transport	Slc14a1	9.58E-23	0.305972519	0.298	0.017	4.57E-18
	Unclear function	Cst6	6.46E-32	0.840908962	0.619	0.225	3.08E-27
		Esm1	1.35E-11	0.389527287	0.39	0.176	6.45E-07
		Pxdc1	3.41E-11	0.356619506	0.741	0.567	1.63E-06
		AA467197	2.36E-11	0.343679046	0.637	0.384	1.13E-06
		Ptma	2.79E-21	0.330013367	1	1	1.33E-16
		Dynap	2.54E-19	0.329825574	0.272	0.024	1.21E-14
		810417H13Ri	3.00E-17	0.303323263	0.484	0.176	1.43E-12
Downregulated Genes	Adapter complex	Copz2	8.13E-25	-0.361174898	0.747	0.855	3.88E-20
	Cell commuication	Col4a1	2.13E-08	-0.33213287	0.62	0.706	0.001017273
		Col4a2	3.67E-13	-0.429324193	0.623	0.734	1.75E-08
		Colec12	8.57E-20	-0.319471466	0.638	0.785	4.09E-15
		Fibin	1.35E-38	-0.621850655	0.296	0.689	6.43E-34
		Gem	1.47E-07	-0.30581284	0.715	0.744	0.007031856
		Gng11	8.59E-13	-0.462720045	0.942	0.934	4.10E-08
		Il1rl1	2.38E-08	-0.315811769	0.336	0.481	0.001134531
		Itm2b	3.35E-24	-0.349032163	0.999	1	1.60E-19
		Lsp1	8.47E-26	-0.490091543	0.706	0.865	4.04E-21
		Ly6a	9.28E-09	-0.33223259	0.904	0.903	4.43E-04
		Rnase4	2.19E-31	-0.557339984	0.714	0.9	1.04E-26
	Cell growth	Igfbp7	4.93E-52	-0.999312186	0.522	0.886	2.35E-47
		Wisp2	1.91E-13	-0.476211121	0.227	0.429	9.10E-09
		Aebp1	4.10E-25	-0.427017335	0.951	0.965	1.96E-20
		Crip1	1.32E-15	-0.315703777	0.83	0.938	6.31E-11
		Crlf1	3.86E-33	-0.747653408	0.422	0.754	1.84E-28
		Cst3	1.60E-27	-0.385502195	1	1	7.62E-23
		Ctla2a	6.59E-13	-0.615953403	0.155	0.329	3.15E-08
		Ctsc	7.88E-12	-0.419237006	0.489	0.623	3.76E-07
		Cxcl1	2.28E-08	-0.697522109	0.968	0.941	0.001086723
		Cxcl5	2.85E-14	-0.477013143	0.333	0.529	1.36E-09

	Cell metabolism	Cygb	5.04E-21	-0.423204665	0.313	0.585	2.41E-16
		Dcn	2.55E-24	-0.675041558	0.812	0.903	1.22E-19
		Ecscr	4.85E-18	-0.374026853	0.449	0.682	2.31E-13
		Egr1	5.69E-11	-0.35779974	0.883	0.903	2.72E-06
		Enpp1	1.29E-12	-0.313884205	0.455	0.585	6.15E-08
		Fbln5	9.30E-24	-0.380884813	0.329	0.585	4.44E-19
		Fbn1	2.63E-34	-0.61818172	0.7	0.875	1.25E-29
		Gadd45b	2.90E-16	-0.500362008	0.904	0.952	1.38E-11
		Hspg2	9.95E-21	-0.325009969	0.814	0.882	4.75E-16
		Ifrd1	9.21E-08	-0.337542183	0.974	0.972	0.004396848
		Lrp1	8.52E-32	-0.501359025	0.859	0.924	4.06E-27
		Mfap2	2.52E-27	-0.434493105	0.541	0.806	1.20E-22
		Mfap4	4.59E-34	-0.769085872	0.375	0.699	2.19E-29
		Nsg1	3.61E-32	-0.457578248	0.473	0.779	1.72E-27
		Pim1	1.71E-09	-0.313238043	0.7	0.744	8.17E-05
		Plac8	2.13E-16	-0.65957221	0.453	0.64	1.02E-11
		Pmepa1	3.13E-32	-0.496451786	0.79	0.907	1.49E-27
		Postn	1.13E-24	-0.714431203	0.742	0.848	5.41E-20
		Prg4	2.76E-19	-0.483491845	0.187	0.422	1.32E-14
		Rarres2	7.77E-10	-0.579299018	0.176	0.325	3.71E-05
		Rdh10	1.99E-10	-0.313510627	0.623	0.696	9.50E-06
		Rgcc	4.21E-17	-0.533271939	0.672	0.782	2.01E-12
		Serpinf1	6.19E-25	-0.41087675	0.984	0.993	2.95E-20
		Smoc2	1.98E-10	-0.454739279	0.385	0.561	9.45E-06
		Spock3	6.05E-09	-0.316726928	0.281	0.405	2.89E-04
		Spon1	1.51E-13	-0.433034119	0.285	0.495	7.19E-09
		Thbs4	7.89E-33	-0.719024638	0.433	0.747	3.76E-28
		Thy1	5.46E-32	-0.701003697	0.837	0.92	2.61E-27
		Timp1	2.19E-14	-0.405275349	0.999	0.997	1.04E-09
		Tnc	3.89E-23	-0.599978742	0.839	0.917	1.85E-18
		Wisp1	9.58E-18	-0.309087002	0.521	0.709	4.57E-13
		Cpxm1	1.10E-20	-0.364419857	0.36	0.619	5.24E-16
		Ctsk	5.05E-14	-0.314937229	0.704	0.792	2.41E-09
		Mgst1	3.72E-31	-0.446862769	0.399	0.775	1.78E-26
		Mmp23	1.64E-23	-0.306005551	0.402	0.675	7.81E-19
		Ndufa4l2	1.34E-14	-0.924454646	0.22	0.412	6.41E-10
		Pcolce	5.44E-22	-0.462410681	0.96	0.962	2.60E-17
		Pla1a	2.80E-09	-0.333745698	0.329	0.478	1.34E-04
		Rcn3	2.08E-42	-0.636029546	0.789	0.955	9.94E-38
	Cell migration involved in sprouting angiogenesis	Hmox1	5.50E-11	-0.439994169	0.922	0.927	2.63E-06
		Nrp1	2.05E-31	-0.477871339	0.627	0.858	9.80E-27
		Srpx2	7.86E-29	-0.400442507	0.54	0.824	3.75E-24
	Cell-substrate adhesion	Col8a1	2.60E-17	-0.440917008	0.405	0.64	1.24E-12
		Nid1	4.26E-17	-0.315783745	0.71	0.806	2.03E-12
	Developmental process	Angptl2	1.24E-14	-0.330137394	0.819	0.855	5.91E-10
		Aspn	7.63E-31	-0.529562677	0.249	0.581	3.64E-26
		Bgn	4.95E-33	-0.459657344	0.69	0.858	2.36E-28
		Cdh11	3.56E-26	-0.314013627	0.33	0.626	1.70E-21
		Col15a1	2.22E-13	-0.429291419	0.336	0.54	1.06E-08
		Ctgf	1.67E-09	-0.443589215	0.37	0.512	7.98E-05
		Cthrc1	2.51E-31	-0.804160561	0.69	0.834	1.20E-26
		Cxcl14	3.93E-12	-0.876911536	0.849	0.862	1.88E-07
		Fstl1	5.56E-29	-0.657832847	0.858	0.91	2.66E-24
		Ifi27	9.34E-20	-0.333622792	0.808	0.958	4.46E-15
		Itm2a	1.83E-14	-0.441732087	0.838	0.886	8.71E-10
		Mfap5	3.49E-24	-0.628843585	0.409	0.696	1.67E-19
		Mmp2	1.45E-23	-0.507474325	0.864	0.92	6.93E-19
		Mxra8	6.89E-22	-0.366228623	0.792	0.889	3.29E-17
		Nrep	1.95E-16	-0.33198318	0.363	0.581	9.29E-12
		Sdc2	8.33E-17	-0.381579717	0.955	0.952	3.98E-12
		Selm	6.00E-40	-0.538166868	0.84	0.958	2.87E-35
		Serping1	2.63E-34	-0.72984766	0.553	0.827	1.25E-29
		Sparc	1.15E-41	-0.732636584	0.998	1	5.48E-37
		Sparcl1	2.32E-14	-0.613794955	0.474	0.657	1.11E-09
		Tagln	4.19E-13	-0.634087456	0.225	0.408	2.00E-08
		Tmem176b	1.97E-26	-0.439244928	0.591	0.907	9.39E-22
		Fbln2	1.22E-38	-0.654073046	0.65	0.893	5.83E-34
	Extracellular matrix organization	Col6a1	2.21E-42	-0.765066843	0.652	0.893	1.06E-37
		Col6a2	4.14E-42	-0.761987227	0.552	0.858	1.98E-37
		Tnfaip6	1.59E-18	-0.564666609	0.682	0.799	7.58E-14
		Adamts2	1.06E-23	-0.33667953	0.383	0.668	5.06E-19

	Collagen fibril organization	Col1a1	8.18E-41	-0.820793173	0.721	0.907	3.91E-36
		Col1a2	9.09E-40	-0.765879576	0.905	0.972	4.34E-35
		Col3a1	7.54E-36	-0.796920715	0.961	0.969	3.60E-31
		Col5a1	6.62E-32	-0.517674323	0.622	0.834	3.16E-27
		Col5a2	2.87E-46	-0.749935636	0.788	0.941	1.37E-41
		Dpt	1.29E-08	-0.319930869	0.342	0.474	6.14E-04
		Lum	2.44E-08	-0.414402901	0.492	0.637	0.001163942
		Serpinh1	3.27E-32	-0.484589429	0.998	0.997	1.56E-27
		Sfrp2	1.82E-07	-0.442252421	0.245	0.377	0.008684482
		Col5a3	1.66E-29	-0.485173765	0.48	0.747	7.93E-25
	Golgi-transport	Kdelr3	2.31E-19	-0.32798162	0.818	0.865	1.10E-14
		Tmed3	2.05E-19	-0.313563837	0.975	0.986	9.81E-15
	Insulin-like growth factor receptor signaling	Cilp	4.45E-11	-0.380190983	0.247	0.433	2.12E-06
		Igf1	1.21E-16	-0.36914651	0.259	0.512	5.79E-12
		Igfbp6	5.48E-08	-0.564787441	0.71	0.716	0.002615474
	Molecular chaperone	Fkbp7	8.65E-19	-0.332976137	0.769	0.813	4.13E-14
	Molecular scaffold	Malat1	7.57E-19	-0.397615372	0.998	1	3.61E-14
	Oxidative damage response	Gpx3	3.04E-09	-0.491695306	0.473	0.574	1.45E-04
		Sod3	5.31E-25	-0.645709566	0.399	0.682	2.53E-20
	Peptidyl-lysine oxidation	Lox	9.99E-51	-0.773971603	0.604	0.886	4.77E-46
		Loxl1	6.80E-30	-0.531649412	0.718	0.889	3.25E-25
		Loxl2	5.75E-21	-0.447223239	0.802	0.858	2.74E-16
		Loxl3	2.73E-20	-0.419008975	0.632	0.817	1.30E-15
	Plasminogen activation	Serpine1	5.12E-07	-0.429032409	0.599	0.651	0.02444679
		Serpine2	1.40E-13	-0.500266136	0.63	0.758	6.66E-09
		Thbs1	1.34E-09	-0.392808366	0.742	0.785	6.39E-05
	Plate derived growth factor receptor signaling	Pdgfrl	1.27E-28	-0.409998063	0.353	0.682	6.06E-24
	Protein binding	Fcgrt	4.01E-29	-0.375820126	0.59	0.858	1.91E-24
	Response to IL-1	Ccl11	1.20E-08	-0.565451608	0.355	0.491	5.73E-04
		Ccl2	7.91E-09	-0.523712466	0.897	0.893	3.78E-04
		Ccl7	1.01E-14	-0.531229809	0.828	0.893	4.82E-10
		Il6	6.33E-10	-0.48391598	0.247	0.405	3.02E-05
	Transform growth factor beta production	Fbln1	1.65E-13	-0.300522949	0.492	0.623	7.85E-09
		Fn1	6.06E-32	-0.57760367	0.989	1	2.89E-27
		Ptgs2	1.54E-07	-0.322168365	0.614	0.685	0.007352173
	Unclear function	C1qtnf6	5.89E-26	-0.432613055	0.658	0.82	2.81E-21
		Laptm4a	2.91E-33	-0.386782233	0.998	1	1.39E-28
		Lhfp	5.05E-28	-0.445702902	0.747	0.872	2.41E-23
		Maged2	9.53E-17	-0.308171885	0.81	0.889	4.55E-12
		Medag	1.13E-21	-0.40358075	0.367	0.633	5.39E-17
		Npdc1	8.13E-21	-0.330455393	0.728	0.824	3.88E-16
		Snhg18	9.91E-27	-0.405214461	0.831	0.931	4.73E-22
		Tmem176a	4.62E-22	-0.43642125	0.561	0.813	2.21E-17
